# Safety, health improvement and well-being during a 4 to 21-day fasting period in an observational study including 1422 subjects

**DOI:** 10.1371/journal.pone.0209353

**Published:** 2019-01-02

**Authors:** Françoise Wilhelmi de Toledo, Franziska Grundler, Audrey Bergouignan, Stefan Drinda, Andreas Michalsen

**Affiliations:** 1 Buchinger Wilhelmi Clinic, Überlingen, Germany; 2 Charité–Universitätsmedizin Berlin, corporate member of Freie Universität Berlin, Humboldt-Universität zu Berlin, and Berlin Institute of Health, Berlin, Germany; 3 Division of Endocrinology, Metabolism, and Diabetes and Anschutz Health and Wellness Center, University of Colorado, School of Medicine, Aurora, Colorado, United States of America; 4 Division of Geriatric Medicine, University of Colorado, School of Medicine, Aurora, Colorado, United States of America; 5 Institut Pluridisciplinaire Hubert Curien, Université de Strasbourg, CNRS, Strasbourg, France; 6 UMR 7178 Centre National de la Recherche Scientifique (CNRS), Strasbourg, France; 7 Institute of Social Medicine, Epidemiology and Health Economics, Charité–Universitätsmedizin Berlin, corporate member of Freie Universität Berlin, Humboldt-Universität zu Berlin, and Berlin Institute of Health, Berlin, Germany; 8 Department of Internal and Integrative Medicine, Immanuel Krankenhaus Berlin, Berlin, Germany; Università degli Studi di Milano, ITALY

## Abstract

Only few studies document longer periods of fasting in large cohorts including non-obese participants. The aim of this study was to document prospectively the safety and any changes in basic health and well-being indicators during Buchinger periodic fasting within a specialised clinic. In a one-year observational study 1422 subjects participated in a fasting program consisting of fasting periods of between 4 and 21 days. Subjects were grouped in fasting period lengths of 5, 10, 15 and 20±2 days. The participants fasted according to the Buchinger guidelines with a daily caloric intake of 200–250 kcal accompanied by a moderate-intensity lifestyle program. Clinical parameters as well as adverse effects and well-being were documented daily. Blood examinations before and at the end of the fasting period complemented the pre-post analysis using mixed-effects linear models. Significant reductions in weight, abdominal circumference and blood pressure were observed in the whole group (each p<0.001). A beneficial modulating effect of fasting on blood lipids, glucoregulation and further general health-related blood parameters was shown. In all groups, fasting led to a decrease in blood glucose levels to low norm range and to an increase in ketone bodies levels (each p<0.001), documenting the metabolic switch. An increase in physical and emotional well-being (each p<0.001) and an absence of hunger feeling in 93.2% of the subjects supported the feasibility of prolonged fasting. Among the 404 subjects with pre-existing health-complaints, 341 (84.4%) reported an improvement. Adverse effects were reported in less than 1% of the participants. The results from 1422 subjects showed for the first time that Buchinger periodic fasting lasting from 4 to 21 days is safe and well tolerated. It led to enhancement of emotional and physical well-being and improvements in relevant cardiovascular and general risk factors, as well as subjective health complaints.

## Introduction

There are a growing number of recent publications on intermittent fasting (IF), generally lasting 16 to 48 hours, and calorie restriction (CR). Periodic fasting (PF), recently defined as lasting from 2 to as many as 21 or more days, is less studied in humans, especially for periods longer than 4 days [1, 2]. Results show that lifespan and healthspan are prolonged in several animal models by fasting and CR [2–7] and that parameters of age-related diseases are improved in humans [5, 8, 9]. Fasting leads to pronounced metabolic changes: The shift from carbohydrates and glucose to fatty acids and ketones as the major cellular fuel source for body and brain seems to play a key role. It has recently been referred to as intermittent metabolic switching (IMS) and glucose-to-ketone (G-to-K) switch. The reverse step–ketone-to-glucose (K-to-G) switch–happens upon refeeding [2]. The G-to-K switch includes reduction in blood glucose, insulin and IGF-1 levels, depletion or reduction of glycogen stores, and an increase in lipolysis and ketogenesis [2, 5, 10]. Fasting has been shown to induce differential cellular stress resistance [11] and autophagy [12, 13], as well as triggering the synthesis of detoxification enzymes [9, 14]. Fasting seems to modify the intestinal microbiome [9, 15]. It also leads to changes in the intestinal mucosal walls in rats [16] and to pronounced neuroendocrine adaptation processes [2, 17]. Finally, in the K-to-G switch, fasting has been found to activate stem cells and multiple system regeneration in the refeeding period [4, 18, 19] and to increase the mitochondrial biogenesis in neurons and other body cells [2, 9].

Long periods of fasting, lasting several days to several weeks, are physiologic, e.g. during seasons of low sun exposure, and are still part of the life of most animals [20, 21] as well as of humans living without food conservation technologies [22].

Fasting periods with various patterns are found in most religions [23]. For instance, Ramadan intermittent fasting was linked with improvements in cardiometabolic risk factors [24]. Furthermore, morbid obesity and associated diseases were treated in the 1960s with long periods of fasting that were termed the “zero calorie diet” [25, 26]. In exceptional circumstances these periods could last up to 249 days or more [27, 28]. A medical program of periodic fasting developed by the German physician Otto Buchinger to treat obesity and metabolic and inflammatory pathologies is well-known in central Europe [29–31]. The therapeutic effects of Buchinger periodic fasting are documented in small studies on overweight [32], blood pressure [33], metabolic syndrome [34], fibromyalgia [35], chronic pain syndromes [36] and the enhancement of quality of life [37]. The effects of repeated cycles of Buchinger fasting have also been reported [38, 39].

We are not aware of large studies on PF including normal weight or moderately obese subjects and focused on safety and tolerability. In the present observational study, we documented prospectively the safety, general health-related outcomes and well-being of 1422 subjects. They fasted for periods between 4 to 21 days under medical supervision according to the Buchinger fasting program, as described in peer-reviewed guidelines [31]. The fasting took place in a facility specialized in therapeutic fasting, the Buchinger Wilhelmi clinic (BWC) in Germany. The protocol involved daily clinical monitoring, intake of 2–3 L of water per day and 250 kcal of food, as well as a multi-disciplinary program including health education and physical activity.

The aim of this study was to assess for the first time prospectively the safety, therapeutic efficiency and effects on well-being of Buchinger periodic fasting in a large cohort.

## Materials and methods

### Ethics

This observational and prospective study was approved by the medical council of Baden-Württemberg and the Ethics Committee of the Charité-University Medical Center, Berlin (application number: EA4/054/15) on 5 May 2015. The study protocol was registered in the German Clinical Trials Register (DRKS-ID: DRKS00010111). The authors confirm that all ongoing and related trials for this intervention are registered. At the time of obtaining the ethical approval by the German authorities it was not mandatory to register an observational study. Only randomized clinical trials were clearly recommended to register. Nevertheless, we decided to register this observational study (on 3 June 2016).

Participants were enrolled after giving their written informed consent between 1 January and 31 December 2016. The study was conducted in the BWC in Überlingen (Germany) in accordance with the principles of the Declaration of Helsinki. The follow-up was completed between 26 January 2016 and 18 December 2017.

### Participants

**Fig 1:**
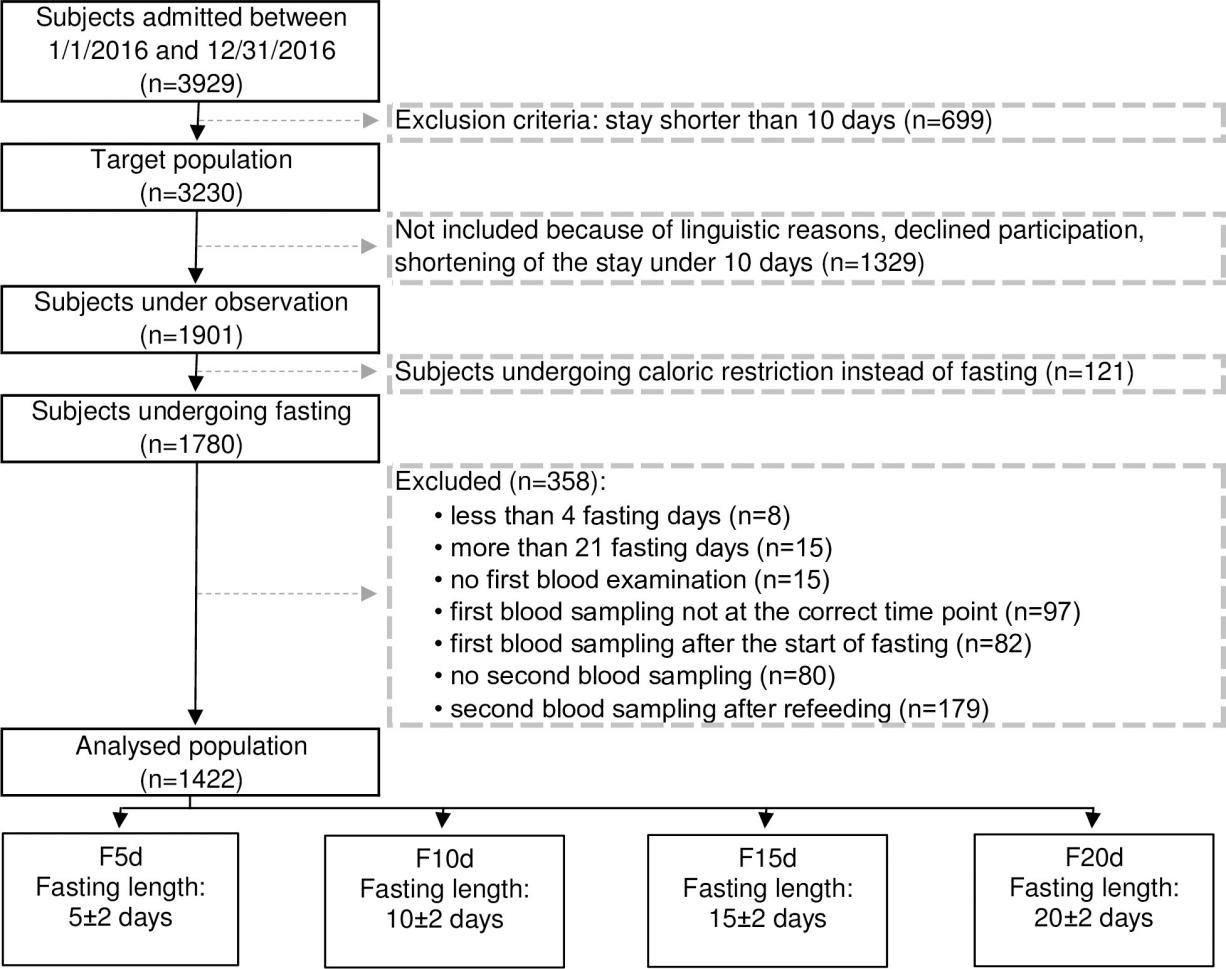
Flow chart of the selection procedure of study participants.

Our study on Buchinger fasting during periods of 4 to 21 days included 1422 subjects. They were selected out of a total of 3929 subjects who were admitted to the BWC and fulfilled the following criteria: they had a clinic stay of at least 10 nights and signed the informed written consent at the beginning of the inpatient stay after confirming that they would not participate in another study. Subjects were aged between 18–99 years and had no predefined contraindication to Buchinger fasting (e.g. cachexia, anorexia nervosa, advanced kidney, liver or cerebrovascular insufficiency, dementia or other severely debilitating cognitive disease and no existing pregnancy or lactation period) [31]. Furthermore, blood must have been collected on the precise days defined in the protocol (S2 and S3 Text). We excluded subjects who were prescribed other diets than fasting according to predefined criteria as well as those who could not follow the study procedures due to an inability to speak German, English or French. A flow chart reflecting the selection procedure is given in Fig 1.

The subjects came voluntarily to the BWC for preventive or therapeutic reasons. They selected established programs of 5, 10, 15 and 20 fasting days, which are reflected in the 4 groups (F5d, F10d, F15d, F20d). The main personal intentions for the fasting intervention were reduction of cardiovascular risk factors, weight loss in case of obesity and relief of general health problems such as inflammatory diseases, distress and exhaustion. In case of prescribed drug intake, the dosage was adapted during the stay by the 8 physicians of the clinic, who examined all participants 2 to 3 times per week. The subjects had a wide diversity of national and cultural backgrounds. The majority of them came from upper social classes and had high education levels.

### Fasting program

All subjects fasted according to the guidelines of the Buchinger fasting therapy [31] under daily supervision of nurses and specialized physicians. On the day before the beginning of the fast, the participants were given a 600 kcal vegetarian diet divided into 3 meals of either rice and vegetables or fruits, according to individual preference. To initiate the fasting period, the intestinal tract was emptied through the intake of a laxative (20–40 g NaSO4 in 500 ml water). During fasting all subjects were asked to drink 3 L of water or non-caloric herbal teas daily with an optional portion of 20 g honey. Additionally, an organic freshly squeezed fruit or vegetable juice (250 ml) was served at noon and a vegetable soup (250 ml) in the evening, leading to an average total calorie intake of 200–250 kcal and 25–35 g of carbohydrates per day. At the beginning of the fasting period the subjects entered a program of light physical exercise alternating with rest and individual mild non-physical treatments like hydrotherapy or physiotherapy. The exercise program consisted of light to moderate intensity outdoor walks and group gymnastics. The whole program was led by certified trainers. During the fasting period an enema or, if preferred by the patient, a mild laxative was applied every second day in order to remove intestinal remnants and desquamated mucosal cells. On the last day of fasting, food was stepwise reintroduced during an average of 4 days, with an ovo-lacto-vegetarian organic diet progressively increasing from 800 to 1600 kcal/day.

### Measurements

To document safety as well as health benefits and well-being during a prolonged periodic fasting program, we performed the predefined following measurements at baseline (pre-) and at the completion of fasting (post-). Before starting the fast all subjects went through a thorough physical examination and their medical history was documented.

Well-being, ketone bodies, mild symptoms and any changes in major health complaints were self-reported under supervision. The results were daily noted in a questionnaire (S4, S5, S6 Text). A total of 1311 subjects out of the 1422 returned the completed questionnaire.

#### Weight, abdominal circumference, blood pressure and pulse

Clinical data were collected by the physicians. Trained nurses documented every morning according to a standardized protocol the body weight of the participants wearing standard clothing (Seca 704, Seca, Hamburg, Germany). Blood pressure and pulse were measured after a pause, once at the non-dominant arm in sitting position (upper arm blood pressure monitor, boso Carat professional, BOSCH + SOHN GmbH u. Co. KG, Jungingen, Germany). Height was assessed with seca 285 (Seca, Hamburg, Germany) and abdominal circumference was determined with a measuring tape mid-way between the lowest rib and the iliac crest (openmindz GmbH, Heidelberg, Germany).

#### Well-being

To evaluate well-being, the participants self-reported daily their physical (PWB) and emotional well-being (EWB) on numeric rating scales from 0 (very bad) to 10 (excellent), under nurses’ supervision. The aim was to document the tolerability of the fasting program.

In a pre-study sample, we evaluated the acceptance of validated questionnaires to assess well-being within the patient population of the BWC, but found that they were regarded as too time-consuming in comparison with the numeric rating scales. To avoid drop-out and missing data, we therefore decided to use numeric rating scales.

#### Ketone bodies

The subjects self-measured the semi-quantitative concentration of ketone bodies in the first morning urine using Ketostix (Bayer AG, Leverkusen, Germany), which reacts according to the concentration of acetoacetic acid.

#### Mild symptoms and adverse effects

The Buchinger periodic fasting program was continuously monitored for safety and supervised by the medical staff: mild symptoms were reported daily by means of a multiple choice questionnaire, completed by the subjects under the supervision of nurses. This questionnaire listed the 19 most frequent mild symptoms that are observed in BWC and mentioned in the guidelines of the Buchinger fasting therapy [31]. We considered a mild symptom as being relevant when it was mentioned at least 3 times. In addition to the listed symptoms, the medical staff reported further mild symptoms that we categorized as “observed symptoms”. Furthermore, occasional adverse effects (AE) were documented by the physicians.

#### Major health complaint

A self-evaluation of health status was undertaken at the end of the stay: the subjects were asked to self-rate any changes in their major health complaint (in cases in which they indicated one at the begin of the fast) during the fasting intervention on a visual numeric scale from 0 (much worse) to 7 (much better).

#### Blood analysis

A blood analysis was taken according to international methods (see below): (lipid parameters: total cholesterol [TC], triglycerides [TG], high-density lipoprotein [HDL-C], low-density cholesterol [LDL], LDL-C/HDL-C ratio [LDL/HDL-ratio]; glycaemia: blood glucose and glycated haemoglobin [HbA1c]; blood count: leukocytes, erythrocytes, haemoglobin, haematocrit, mean cell volume [MCV], mean corpuscular haemoglobin [MCH], mean corpuscular haemoglobin concentration [MCHC], thrombocytes; coagulation: international normalized ratio [INR], Quick, partial thromboplastin time [PTT]; liver function: serum glutamic oxaloacetic transaminase [GOT], serum glutamate pyruvate transaminase [GPT], serum gamma-glutamyl transferase [GGT], alkaline phosphatase [AP]; inflammatory biomarkers: C-reactive protein [CRP], erythrocyte sedimentation rate [ESR] after 1 and 2 hours; renal function: uric acid, urea and creatinine and electrolytes: sodium [Na], potassium [K], calcium [Ca], magnesium [Mg]).

### Laboratory examinations

Blood samples were collected twice, namely before the start of the fasting (thereafter referred as baseline values) and at the end of the fasting period. They were collected by trained medical-technical assistants between 7.30 and 9.30 am and drawn into EDTA (S-Monovette 2.7 ml K3 EDTA), citrate (S-Monovette 3 ml 9NC, Citrate 3.2% [1:10]) and blood sedimentation tubes (S-Sedivette 3.5 ml 4NC, ESR/Citrate Buffer [1:5]), that were shaken gently after filling. Additionally, serum tubes including serum gel with clotting activator (S-Monovette 9 ml Z-Gel) were used and stored upright for 30 min until coagulation, with subsequent centrifugation at 3920 g (5000 rpm) for 10 min at room temperature. All tubes were manufactured by Sarsteadt AG & Co. (Nürnbrecht, Germany).

The ESR was assessed within a period of 4 hours after blood collection and determined after 1 and 2 hours of blood sedimentation. All further analyses were performed at MVZ Labor Ravensburg, according to the manufacturer’s instruction, in a fully- automated laboratory. Blood cell count (leukocytes, erythrocytes, haemoglobin, MCV, MCH, MCHC, thrombocytes) was measured using the blood analyser Sysmex XN-9000 (Sysmex Europe GmbH, Norderstedt, Germany). Coagulation parameters (INR, Quick, PTT) were assessed on ACL Top (Werfen, Kirchheim, Germany). The liver enzymes (GOT, GPT, GGT, AP), kidney parameters (urea, creatinine, uric acid), lipid parameters (TC, TG, HDL-C, LDL-C, LDL/HDL ratio), electrolytes (Na, K, Ca), glucose and CRP were analysed with ADVIA 2400 (Siemens Healthcare GmbH, Erlangen, Germany). The HbA1c was assessed with TOSOH^TM^ (Bio-Rad Laboratories GmbH, München, Germany) and Mg with ICP-MS 7700x series (Agilent, Waldbronn, Germany).

### Data and statistical analysis

Participants were divided into four groups according to the duration of their fasting period (Fig 1): F5d underwent a fasting period of 5±2 days, with an average of 5.4 (n = 695), F10d underwent a fasting period of 10±2 days, with an average of 8.6 (n = 530), F15d underwent a fasting period of 15±2 days, with an average of 14.1 (n = 196) and F20d underwent a fasting period of 20±2 days, with an average of 20.1 (n = 37). Between-group differences at baseline were tested using a one-way ANOVA test followed by Tukey’s post-hoc tests.

We tested the effect of fasting, while taking into account the sex and fasting duration group effects, by using a multistep parsimonious statistical approach. First, for each outcome the effect of fasting was assessed by using mixed linear models taking repeated measurements among subjects into account, with fasting intervention, fasting duration group, sex, fasting duration group-by-fasting-intervention, fasting intervention-by-sex, sex-by-fasting duration group and baseline values of the outcome (pre-fasting) as fixed effects. For each outcome, the covariance structures was selected among three (compound symmetry (CS), autoregressive (AR(1)) and variance components (VC)) using the Bayesian information criteria (BIC). In a last step the interaction effects that were not significant, were removed from the model to obtain a more parsimonious model. To simplify the presentation of the results, sex differences are presented in figures only when the fasting-intervention-by-sex effect was significant. To take into account the multiple tests performed on this dataset, significance was set at a conservative level of p<0.01.

Data are shown as mean±standard error of the mean (SEM), if not indicated otherwise. Statistical analyses were performed with SAS version 9.4 (SAS Institute, Cary, USA). Graphs were generated using GraphPad Prism version 6 for Windows (GraphPad Software, La Jolla California USA).

## Results

### General parameters

The baseline characteristics of the 1422 adult participants are shown in Table 1. Mean age was 55.4±0.4 with 59.1% women and 40.9% men. In total 63.4% of the subjects were non-obese (BMI<30). Grade I obesity (30≤BMI<35) was present in 19.5% and grade II or higher (BMI≥35) in 10.3%. Subjects who chose to fast on average for 20 days (F20d) had the highest baseline body mass index (BMI), the highest abdominal circumference (waist), and largest weight reduction (-8.6±0.3 kg) (each p<0.001). Men had a higher mean BMI at baseline (Table 1).

**Table 1:** Baseline characteristics of the subjects.

	All	F5d	F10d	F15d	F20d
Days (d)		5±2	10±2	15±2	20±2
Subjects, n (%)	1422 (100.0)	659 (46.3)	530 (37.3)	196 (13.8)	37 (2.6)
Men (%)	581 (40.9)	278 (42.2)	214 (40.4)	76 (38.8)	13 (35.1)
Women (%)	841 (59.1)	381 (57.8)	316 (59.6)	120 (61.2)	24 (64.9)
Age, years	55.4±0.4	54.2±0.5 ^b^^,^^c^	56.3±0.6 ^a^	56.4±0.9 ^a^	56.4±2.3
Fasting length (days)	8.2±0.1	5.4±0.0 ^b^^,^^c^^,^^d^	8.6±0.0 ^a^^,^^c^^,^^d^	14.1±0.1 ^a^^,^^b^^,^^d^	20.1±0.2 ^a^^,^^b^^,^^c^
Waist, cm	94.0±0.4	91.3±0.6 ^b^^,^^c^^,^^d^	94.8±0.7 ^a^^,^^c^^,^^d^	98.3±1.2 ^a^^,^^b^^,^^d^	106.3±2.8 ^a^^,^^b^^,^^c^
Weight, kg	82.0±0.5	79.3±0.8 ^b^^,^^c^^,^^d^	82.7±0.9 ^a^^,^^c^^,^^d^	86.6±1.6 ^a^^,^^b^^,^^d^	96.7±4.0 ^a^^,^^b^^,^^c^
BMI, kg/m^2^	28.2±0.2	27.2±0.2 ^b^^,^^c^^,^^d^	28.5±0.3 ^a^^,^^c^^,^^d^	29.7±0.4 ^a^^,^^b^^,^^d^	33.6±1.1 ^a^^,^^b^^,^^c^
BMI<25, n (%)	404 (28.4)	227 (56.2)	133 (32.9)	41 (10.1)	3 (0.7)
25≤BMI<30, n (%)	497 (35.0)	232 (46.7)	199 (40.0)	61 (12.3)	5 (1.0)
BMI≥30, n (%)	425 (29.9)	155 (36.5)	160 (37.6)	84 (19.8)	26 (6.1)
BMI men, kg/m^2^	30.0±0.2	29.2±0.3	30.3±0.3	31.3±0.7	34.0±1.5
BMI<25, n (%)	74 (12.7)	46 (62.2)	18 (24.3)	10 (13.5)	0 (0.0)
25≤BMI<30, n (%)	231 (39.8)	117 (50.6)	92 (39.8)	20 (8.7)	2 (0.9)
BMI≥30, n (%)	230 (39.6)	94 (40.9)	87 (37.8)	40 (17.4)	9 (3.9)
BMI women, kg/m^2^	27.0±0.2	25.7±0.2	27.3±0.3	28.7±0.5	33.3±1.4
BMI<25, n (%)	330 (39.2)	181 (54.8)	115 (34.8)	31 (9.4)	3 (0.9)
25≤BMI<30, n (%)	266 (31.6)	115 (43.2)	107 (40.2)	41 (15.4)	3 (1.1)
BMI≥30, n (%)	195 (23.2)	61 (31.3)	73 (37.4)	44 (22.6)	17 (8.7)

Subjects were divided into 4 groups according to the fasting lengths: 5, 10, 15 and 20±2 days. Significant differences between the groups are indicated as

a, p<0.05 versus F5d

b, p<0.05 versus F10d

c, p<0.05 versus F15d

d, p<0.05 versus F20d. BMI, body mass index. Data are presented as mean±SEM.

### Weight, abdominal circumference and blood pressure

**Fig 2:**
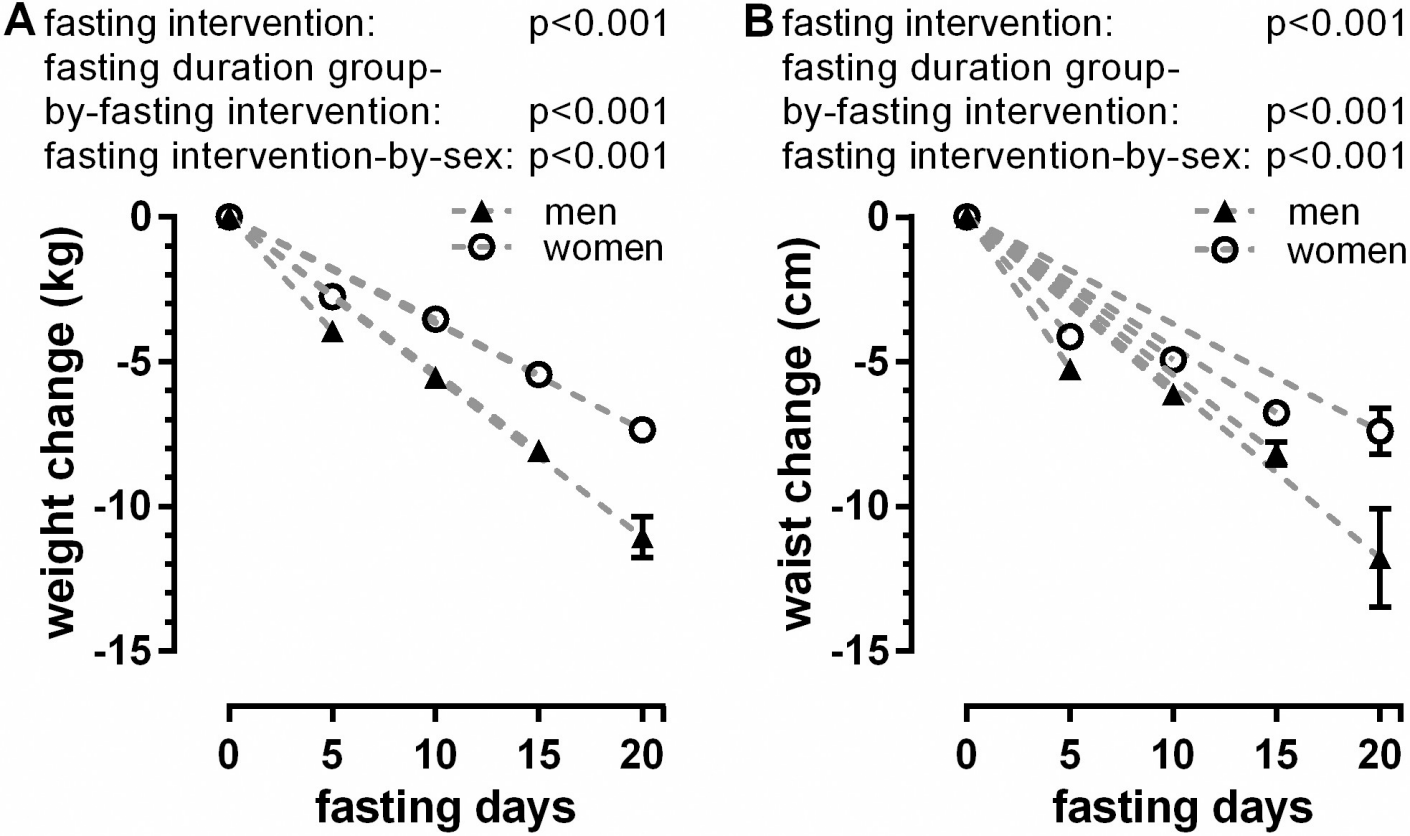
Changes in weight (A) and abdominal circumference (B) according to the length of fast and gender.

As expected, weight and BMI showed a significant decrease (fasting intervention: p<0.001) in all 4 groups (S1 Table). The weight loss increased with the fasting period length and varied between 3.2±0.0 kg for F5d and 8.6±0.3 kg for F20d (fasting duration group-by-fasting intervention: p<0.001). Abdominal circumference also decreased significantly (fasting intervention: p<0.001). The reduction varied between 4.6±0.1 cm for F5d and 8.8±0.8 cm for F20d (fasting duration group-by-fasting intervention: p<0.001). Weight and abdominal circumference reduction were significantly higher (fasting-intervention-by-sex: each p<0.001) in men in all groups (Fig 2A and 2B), compared with women.

**Fig 3:**
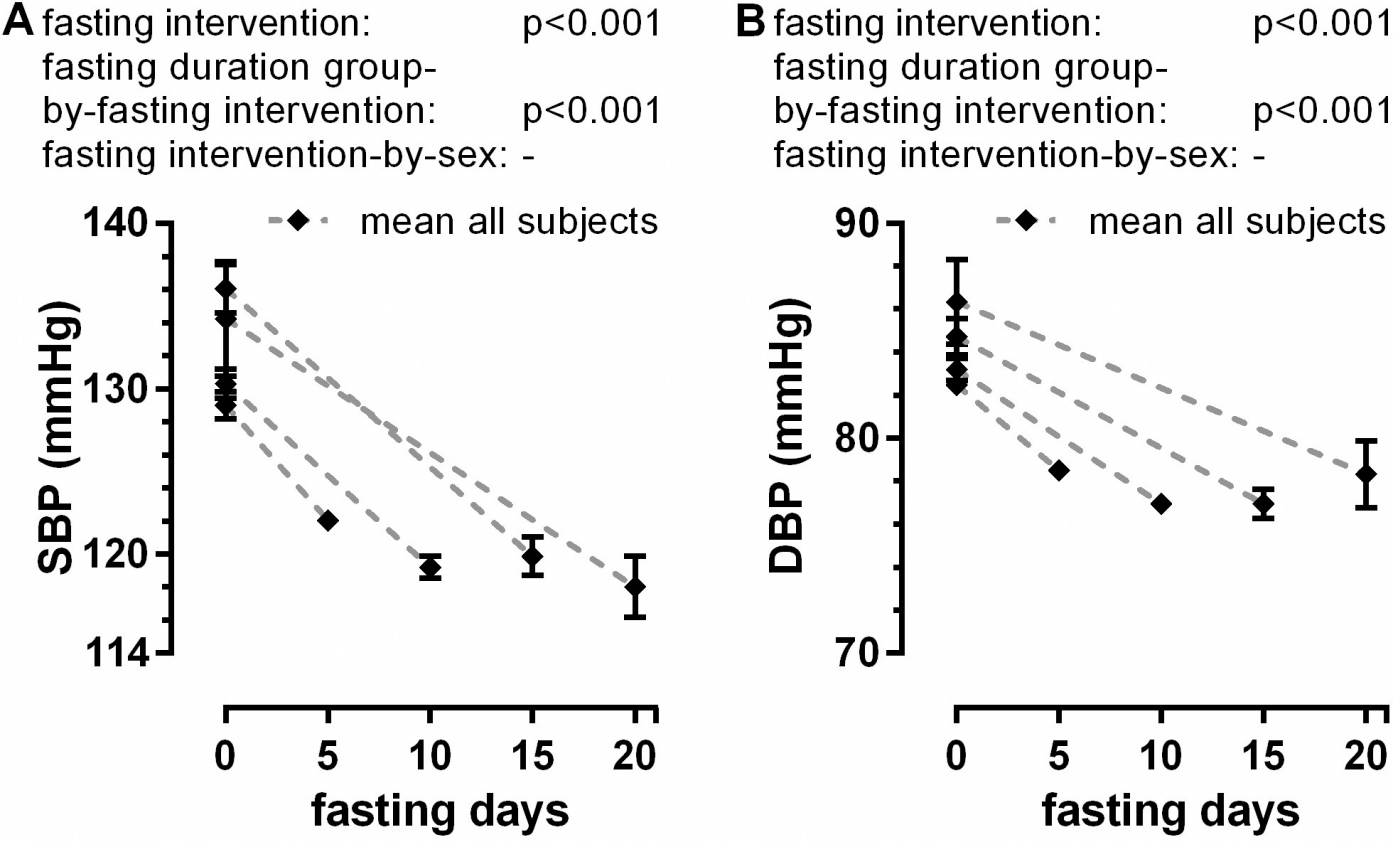
Changes in systolic (A) and diastolic blood pressure (B) according to the fasting length.

Baseline values of systolic blood pressure (SBP) and diastolic blood pressure (DBP) were higher in the groups fasting longer (Fig 3A and 3B). The mean values for the whole cohort decreased significantly from 131.6±0.7 to 120.7±0.4 for SBP (fasting intervention: p<0.001) and from 83.7±0.4 to 77.9±0.3 for DBP (fasting intervention: p<0.001). The reduction of SBP and DBP was greater in the groups who fasted longer (fasting duration group-by-fasting intervention: each p<0.001) without gender difference (Fig 3A and 3B), stabilizing for the whole cohort around 120/78 mm Hg (S1 Table). We did not observe significant changes in heart rate during fasting in the whole group (S1 Table).

### Well-being

**Fig 4:**
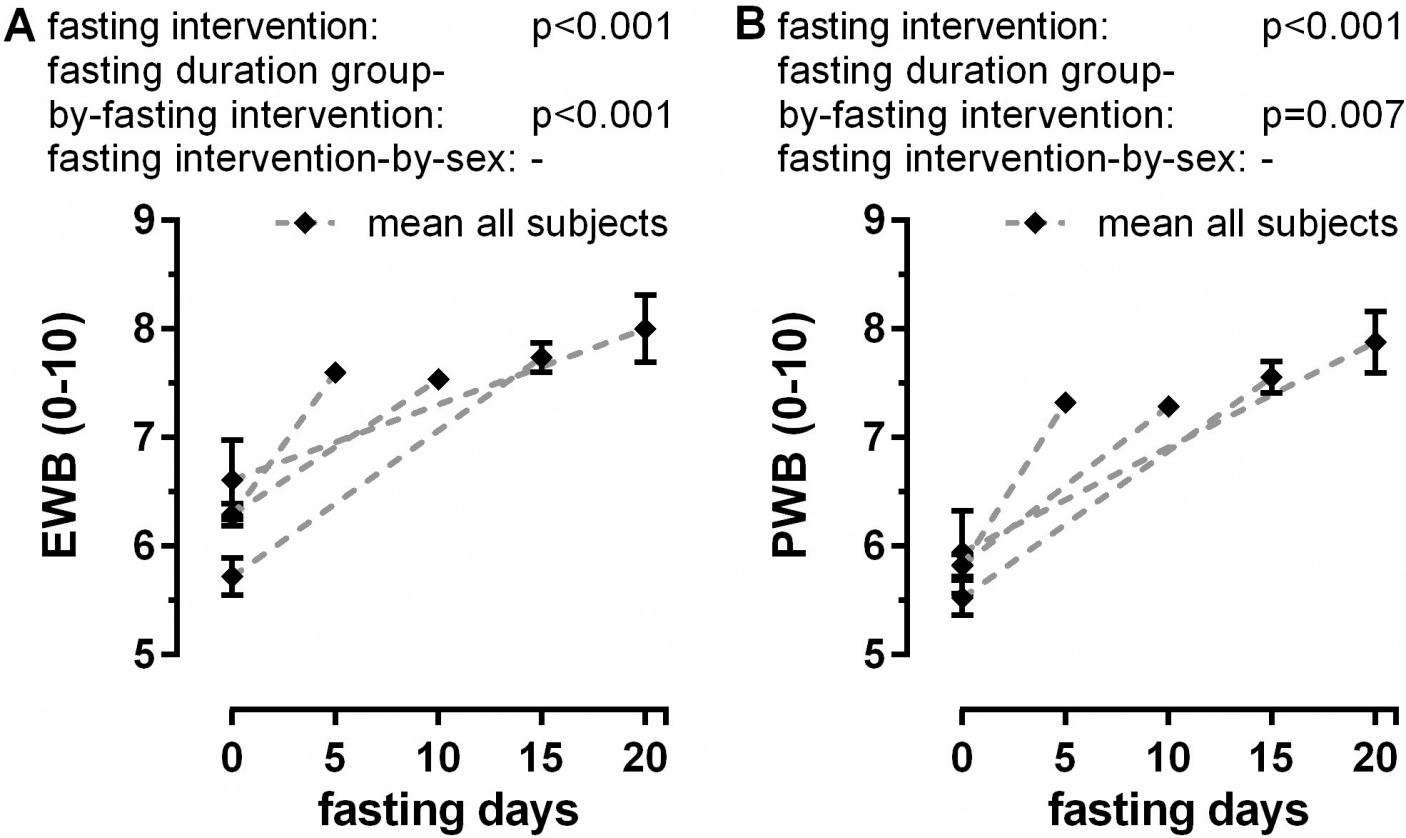
**Changes in emotional (A) and physical well-being (B) during fasting.** Self-recorded data of a 0–10 numeric rating scale for a total of 1074 volunteers are shown.

Baseline values of emotional well-being (EWB) and physical well-being (PWB) were lower in the groups that fasted longer. This suggests that subjects choosing longer fasting periods had lower emotional and physical self-ratings at baseline than the ones who selected shorter periods of fast. EWB as well as PWB were both significantly enhanced in the course of the fast (fasting intervention: each p<0.001) (S1 Table). There is no difference between genders for those parameters (Fig 4A and 4B). All groups reached similar increased values of well-being at the end of their stay.

### Ketone bodies

Acetoacetic acid, reflecting ketosis, increased significantly from baseline to the end of fast (fasting intervention: p<0.001), suggesting a plateau value reached after 5 days. Men had higher scores of acetoacetic acid than women (fasting intervention-by-sex: p<0.001) (S1 Table).

### Mild symptoms and adverse effects

The safety of the Buchinger fasting program was assessed by collecting daily all self-reported and observed mild symptoms (Table 2). Of the 1311 participants who returned the filled questionnaire, 0.35% reported muscular cramp, which was the least frequent mild symptom, and 14.94% sleep disturbances, which was the most frequent mild symptom. As shown in S1 Fig. the incidence of mild symptoms like muscle pain, sleep disturbances, headaches, and hunger occurred mainly in the first days of the fast.

**Table 2:** Mild symptoms and adverse effects (AE).

Mild symptoms (self-reported)	n	%	Mild symptoms (observed)	n	%	
Sleep Disturbance	169	14.94	Dizziness	2	0.14	
Fatigue	155	13.70	Eczema	2	0.14	
Dry Mouth	100	8.84	Bleeding gums	1	0.07	
Back Pain	84	7.43	Hyperventilation	1	0.07	
Hunger	77	6.81	Outbreak of infection	1	0.07	
Bad Breath	61	5.39	Pleuropneumonia	1	0.07	
Headache	61	5.39	Tetany	1	0.07	
Muscle Pain	49	4.33	Visual disorder	1	0.07	
Abdominal Bloating	47	4.16				
Diarrhoea	38	3.36				
Sensitivity to Cold	33	2.92	**AE**	**n**	**%**	
Cravings	29	2.56	Cardiac arrhythmia	3	0.21	
Vertigo	28	2.48	Hyponatremia	3	0.21	
Blurred Vision	23	2.03	Hospitalisation	2	0.14	
Restless Legs	23	2.03	Hypoglycaemia	2	0.14	
Skin Rash	19	1.68	Hypokalaemia	1	0.07	
Nausea	13	1.15	Gout	1	0.07	
Palpitation	13	1.15	Vomiting	1	0.07	
Dyspepsia	12	1.06	Spasmodic abdominal pain	1	0.07	
Muscular cramp	4	0.35				

Out of the total of 1422 a group of 1311 subjects completed and returned the daily questionnaire to self-record mild symptoms. In contrast to the observed symptoms and AE, self-reported mild symptoms were recorded if a particular symptom was experienced by the same person more than 3 times during the fasting period. The observed symptoms and AE were documented during the daily nurse visit and/or the medical visit for all subjects. The same subject could mention more than one symptom or AE.

No fatalities or permanent adverse effects were observed. Two subjects had to be admitted to hospital. A 75-year old man with known coronary artery disease had on the 9^th^ fasting day a non-ST segment elevation myocardial infarction and received uncomplicated percutaneous coronary intervention. After 3 days in the hospital he returned to BWC. The second case was a 67-year old woman who had a one-day hospitalisation because of vomiting with dizziness and diarrhoea on the 4^th^ fasting day. After returning to BWC she received an 800 kcal/d diet. The other AE were transitory and did not lead to an interruption of the fasting therapy. AE (Table 2) such as cardiac arrhythmia were low-grade, transitory and could be treated uncomplicatedly without stopping the fasting. The same applied to transitory hypoglycaemia. We also observed one case of gout attack in a patient treated previous to the fasting with allopurinol for hyperuricemia and frequent gout attacks. He was able to be symptomatically treated and went on fasting.

### Major health complaint

To document the effects of the Buchinger fasting program on their health we asked the participants to self-report if they had a major health complaint before the fasting and how this condition had been influenced by the fast. A group of 404 subjects out of the 1311 who returned the self-report (S2 Fig) mentioned having a major health complaint previous to the fasting. They were asked to evaluate the changes after the fasting. In 84.4% of the 404 subjects the major health complaint had much improved, 8.7% reported that it remained unchanged and 6.9% reported a worsening.

### Blood lipids and glycaemia

We assessed the impact of the Buchinger fasting program on metabolism by analysing several blood parameters (S2 Table).

**Fig 5:**
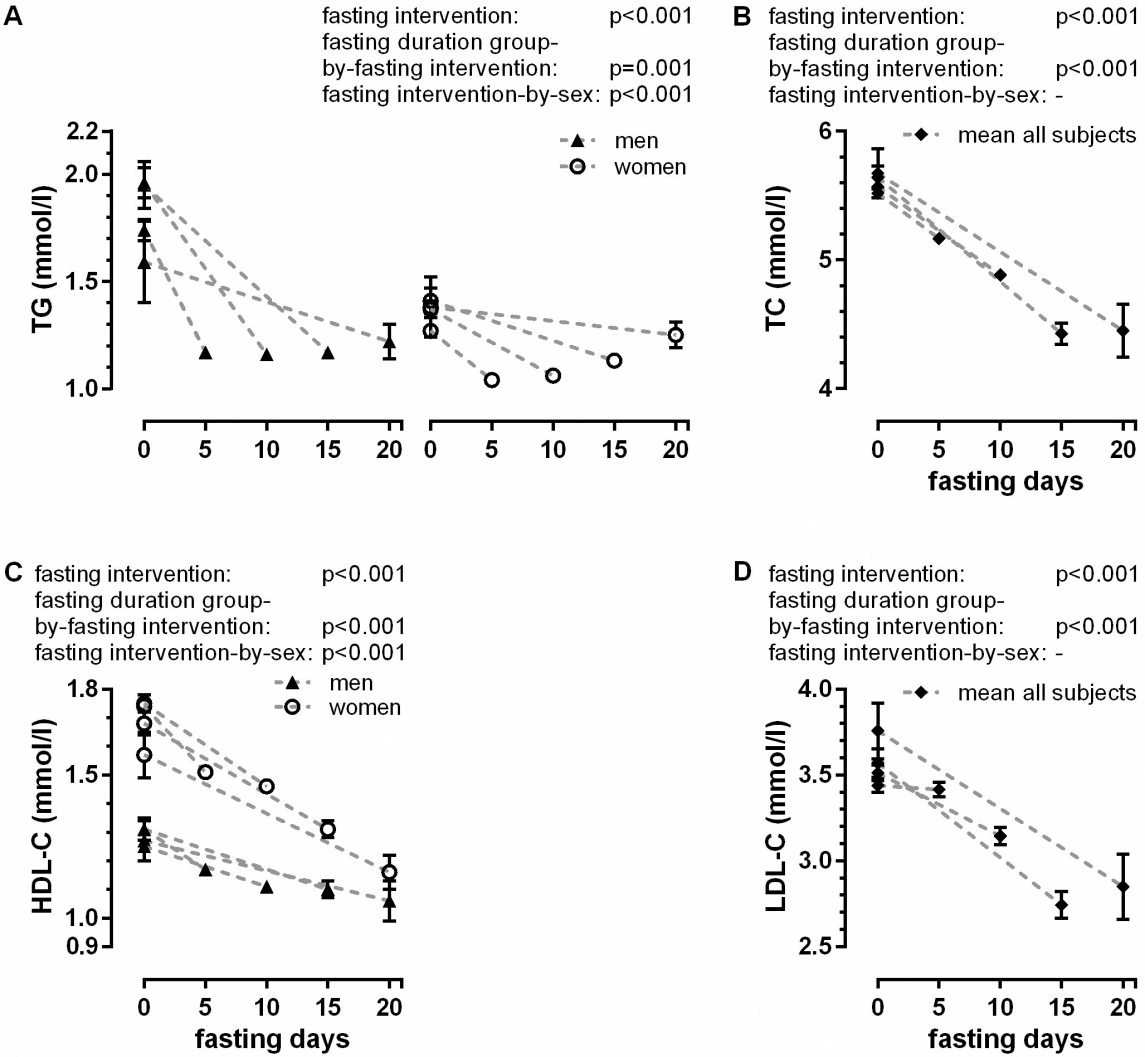
Fasting-induced changes in lipid metabolism.

The lipid values are indicated in S2 Table and Fig 5. At baseline, TG values of women were lower than the values of men. Fasting reduced TG levels by 0.44 mmol/L on average (fasting intervention: p<0.001) (S2 Table). TG levels at the end of the fasting were similar in all groups, suggesting a floor effect (Fig 5A). The decrease in TC was significant (fasting intervention: p<0.001) and higher in the groups who fasted for longer (fasting duration group-by-fasting intervention: p<0.001). Fig 5B indicates that F15d and F20d had similar post-values. There was no difference in TC changes during fasting between men and women. Baseline HDL-C values were higher in women (Fig 5C). HDL-C decreased significantly (fasting intervention: p<0.001). The reduction was higher in the groups that fasted longer (fasting duration group-by-fasting intervention: p<0.001) and in women compared to men (fasting intervention-by-sex: p<0.001). LDL-C decreased significantly (fasting intervention: p<0.001) (Fig 5D) and again the decrease was higher in the groups that fasted longer (fasting duration group-by-fasting intervention: p<0.001). Gender differences for LDL-C were not significant. The LDL/HDL ratio was not influenced by fasting.

**Fig 6:**
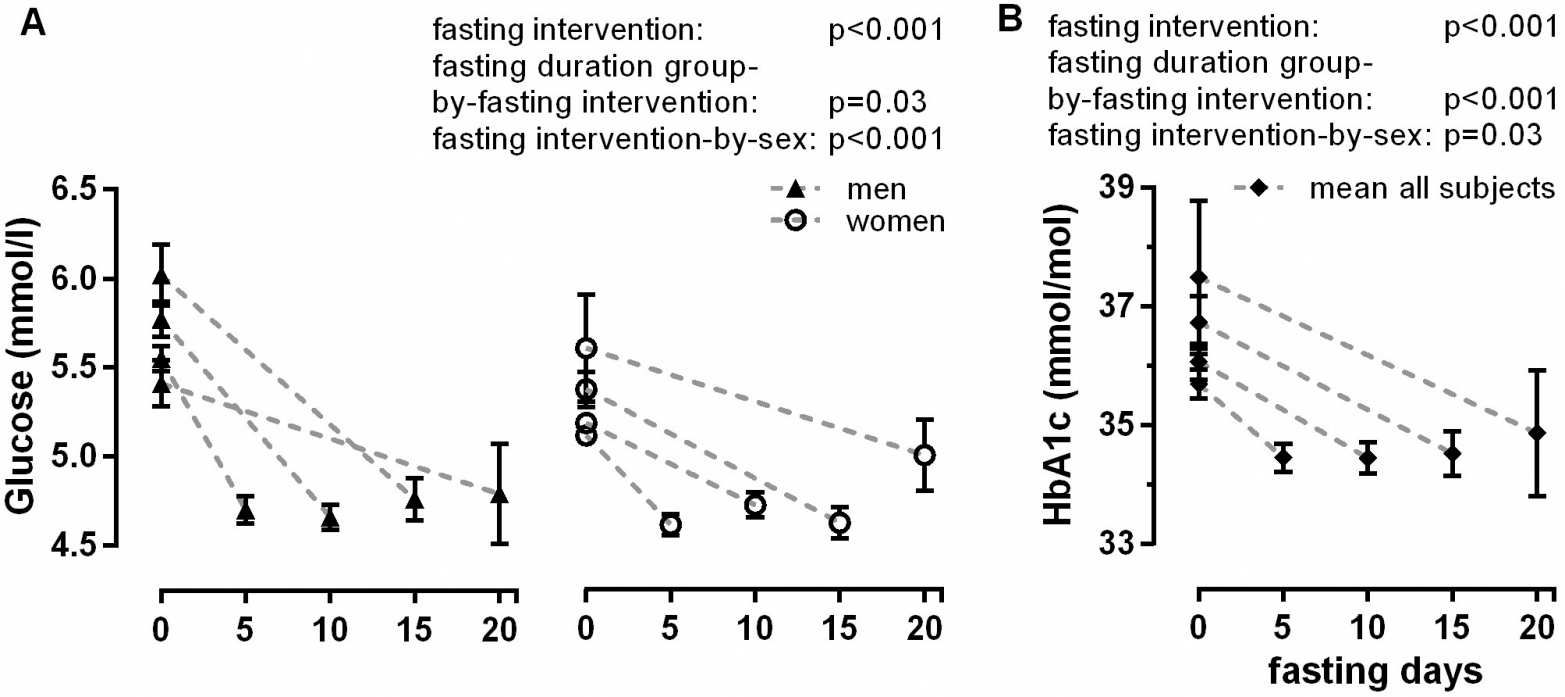
**Changes of blood glucose (A) and glycated haemoglobin (HbA1c) (B).** The panel A was split for gender to increase the readability of the figure.

The blood glucose parameters are given in S2 Table and Fig 6. Baseline values for glucose were higher in men compared to women (Fig 6). The glucose values decreased significantly (fasting intervention: p<0.001) without differences between the fasting period lengths and stabilized at an average of 4.7 mmol/L (S2 Table). Fig 6B shows the significant decrease in HbA1c (fasting intervention: p<0.001), which varied between a decrease of 1.2±0.1 for F5d and 2.6±0.5 mmol/mol for F20d (fasting duration group-by-fasting intervention: p<0.001).

### Blood count

Table 3 shows the impact of fasting on blood count. Leucocytes decreased significantly in all groups (fasting intervention: p<0.001) with stronger reduction in the groups who fasted longer (fasting duration group-by-fasting intervention: p<0.001) and without statistical significance between men and women. Erythrocytes showed an increase (fasting intervention: p<0.001) of an average of 0.06 x10^6^/μl in all groups and steadied at around 4.82x10^6^/μl. Haemoglobin showed also an increase (fasting intervention: p<0.001) of about 0.1 mmol/L that was independent of the fasting length. Haematocrit was not influenced by fasting. Thrombocytes showed a significant reduction (fasting intervention: p<0.001) during fasting by a mean of 6.6±0.7x10^3^/μl, with gender difference (fasting intervention-by-sex: p<0.001) and influence of the fasting length (fasting duration group-by-fasting intervention: p<0.001).

**Table 3:** Blood cells pre- and post-fasting.

	All (n = 1422)		F5d 5±2 d		F10d 10±2 d		F15d 15±2 d		F20d 20±2 d		p-values				
	pre	post	pre	post	pre	post	Pre	post	pre	post	fasting inter- vention	fasting dura- tion group	sex	fasting dura- tion group-by-fasting inter- vention	fasting inter- vention-by-sex
Leukocytes, 10^3^/μl	5.9±0.0	5.4±0.0	5.9±0.1	5.5±0.1	6.0±0.1	5.4±0.1	6.0±0.1	5.0±0.1	5.7±0.3	4.7±0.2	<0.001	<0.001	0.18	<0.001	‒
Erythrocytes, 10^6^/μl	4.76±0.01	4.82±0.01	4.76±0.02	4.82±0.02	4.76±0.02	4.81±0.02	4.74±0.03	4.81±0.03	4.74±0.07	4.86±0.07	<0.001	0.96	<0.001	‒	0.001
Haemoglobin, mmol/L	8.9±0.0	9.0±0.0	8.9±0.0	9.0±0.0	8.9±0.0	9.0±0.0	8.9±0.1	9.0±0.0	8.7±0.1	8.9±0.1	<0.001	0.72	0.33	‒	<0.001
Haematocrit, %	42.2±0.1	42.3±0.1	42.2±0.1	42.3±0.1	42.3±0.1	42.3±0.1	42.2±0.2	42.3±0.2	41.8±0.5	42.2±0.5	0.74	0.83	0.06	‒	0.02
MCV, fl	89.0±0.1	88.0±0.1	88.8±0.2	87.9±0.2	89.1±0.2	88.0±0.2	89.3±0.3	88.0±0.3	88.5±0.9	87.0±0.8	<0.001	0.001	<0.001	0.001	<0.001
MCH, pg	30.1±0.1	30.1±0.1	30.1±0.1	30.1±0.1	30.2±0.1	30.2±0.1	30.2±0.1	30.1±0.1	29.7±0.3	29.7±0.3	0.38	0.12	0.82	0.09	‒
MCHC, g/dl	33.9±0.0	34.2±0.0	33.9±0.0	34.2±0.0	33.9±0.0	34.3±0.0	33.8±0.1	34.2±0.1	33.6±0.2	34.0±0.1	<0.001	0.80	0.11	‒	0.004
Thrombocytes, 10^3^/μl	244.1±1.5	237.5±1.5	242.6±2.2	239.2±2.2	245.9±2.5	238.9±2.6	243.9±3.9	230.6±4.1	245.6±11.4	224.4±11.4	<0.001	<0.001	0.03	<0.001	<0.001

Values are shown as mean±SEM for all of the groups with different fasting lengths. P-values were calculated for the effects of fasting intervention as well as the effects of the fasting length (fasting duration group) and gender (sex). Interactions between fasting intervention by fasting duration group (fasting duration group-by-fasting intervention) and fasting intervention by gender (fasting intervention-by-sex) are shown.

MCV, mean cell volume; MCH, mean corpuscular haemoglobin; MCHC, mean corpuscular haemoglobin concentration.

### Coagulation

Table 4 shows changes in blood coagulation parameters, liver function, inflammatory parameters, kidney function and electrolytes. INR and PTT increased (fasting intervention: each p<0.001) and Quick value decreased (fasting intervention: p<0.001) significantly during fasting. The fasting period length had a significant influence on the coagulation parameters (fasting duration group-by-fasting intervention: each p<0.001) and more pronounced changes were observed in groups of longer fasting periods.

**Table 4:** Blood parameters pre- and post-fasting.

	All (n = 1422)		F5d 5±2 d		F10d 10±2 d		F15d 15±2 d		F20d 20±2 d		p-values				
	pre	post	pre	post	pre	post	Pre	post	pre	post	fasting inter- vention	fasting dura- tion group	sex	fasting dura- tion group-by-fasting inter- vention	fasting inter- vention-by-sex
INR	0.99±0.00	1.08±0.00	0.98±0.00	1.06±0.00	0.99±0.01	1.09±0.01	0.99±0.02	1.11±0.02	0.98±0.01	1.10±0.02	<0.001	<0.001	0.84	<0.001	‒
Quick, %	104.3±0.3	91.4±0.3	104.2±0.5	92.9±0.4	104.0±0.6	90.6±0.5	105.6±1.0	89.1±0.9	104.1±1.2	88.3±1.9	<0.001	<0.001	0.33	<0.001	0.005
PTT, sec	31.1±0.1	32.7±0.1	31.1±0.1	32.4±0.1	31.0±0.1	32.8±0.2	31.2±0.2	33.7±0.3	31.4±0.4	32.9±0.5	<0.001	<0.001	0.88	<0.001	‒
GOT, μkat/L	0.4±0.0	0.6±0.0	0.4±0.0	0.6±0.0	0.4±0.0	0.6±0.0	0.4±0.0	0.6±0.0	0.4±0.0	0.7±0.0	<0.001	0.73	0.007	‒	<0.001
GPT, μkat/L	0.5±0.0	0.7±0.0	0.5±0.0	0.6±0.0	0.5±0.0	0.7±0.0	0.5±0.0	0.7±0.0	0.6±0.0	0.8±0.1	<0.001	0.07	0.65	0.10	‒
GGT, μkat/L	0.6±0.0	0.4±0.0	0.5±0.0	0.4±0.0	0.6±0.1	0.4±0.0	0.6±0.1	0.4±0.0	0.5±0.1	0.4±0.01	<0.001	<0.001	0.18	<0.001	<0.001
AP, μkat/L	1.1±0.0	1.0±0.0	1.0±0.0	1.0±0.0	1.1±0.0	1.1±0.0	1.1±0.0	1.0±0.0	1.2±0.1	1.1±0.1	<0.001	<0.001	0.003	<0.001	0.002
CRP, mg/L	2.85±0.14	4.30±0.20	2.49±0.19	4.11±0.27	3.02±0.28	4.35±0.36	3.37±0.32	4.74±0.63	4.08±0.67	4.67±0.82	0.001	0.97	0.99	0.81	0.53
ESR 1h	11.6±0.2	11.4±0.2	10.9±0.3	11.7±0.3	11.8±0.4	11.5±0.4	12.8±0.7	10.6±0.6	15.1±1.6	10.2±1.4	<0.001	<0.001	0.002	<0.001	<0.001
ESR 2h	21.7±0.4	21.3±0.4	20.4±0.5	21.8±0.5	22.1±0.6	21.4±0.6	23.9±1.1	19.7±1.0	28.2±2.8	20.1±2.4	<0.001	<0.001	<0.001	<0.001	<0.001
Uric acid, μmol/L	338.1±2.3	495.2±4.4	334.0±3.3	481.1±6.0	339.2±3.8	505.5±7.5	345.3±6.4	513.0±13.5	355.9±12.8	505.4±30.6	<0.001	0.01	<0.001	0.01	<0.001
Urea, mmol/L	4.7±0.0	3.1±0.0	4.6±0.1	3.3±0.1	4.7±0.1	3.0±0.1	4.7±0.1	2.7±0.1	5.1±0.3	2.8±0.3	<0.001	<0.001	<0.001	<0.001	<0.001
Creatinine, μmol/L	71.92±0.40	76.43±0.54	72.53±0.58	76.45±0.76	71.88±0.68	76.88±1.00	69.86±0.98	74.98±1.21	72.27±2.53	77.17±3.13	<0.001	0.34	<0.001	‒	<0.001
Na,mmol/L	140.1±0.1	138.7±0.1	140.0±0.1	138.4±0.1	140.1±0.1	138.8±0.1	139.8±0.3	139.2±0.2	141.0±0.33	139.9±0.4	<0.001	<0.001	0.37	0.003	‒
K, mmol/L	4.4±0.0	4.4±0.0	4.4±0.0	4.4±0.0	4.4±0.0	4.4±0.0	4.3±0.0	4.4±0.0	4.4±0.1	4.4±0.1	0.001	0.74	<0.001	‒	0.008
Ca, mmol/L	2.32±0.00	2.38±0.00	2.33±0.00	2.36±0.00	2.32±0.00	2.39±0.00	2.32±0.01	2.39±0.01	2.33±0.02	2.40±0.02	<0.001	<0.001	0.14	<0.001	‒
Mg, mmol/L	0.86±0.00	0.87±0.00	0.87±0.00	0.89±0.00	0.87±0.00	0.87±0.00	0.85±0.00	0.86±0.01	0.86±0.01	0.85±0.01	0.09	<0.001	<0.001	<0.001	<0.001

Values are shown as mean±SEM for all of the groups with different fasting lengths. P-values were calculated for the effects of fasting (fasting intervention) as well as the effects of the fasting length (fasting duration group) and gender (sex). Interactions between fasting intervention by fasting duration group (fasting duration group-by-fasting intervention) and fasting intervention by gender (fasting intervention-by-sex) are shown.

INR, international normalized ratio; PTT, partial thromboplastin time; GOT, serum glutamic oxaloacetic transaminase; GPT, serum glutamate pyruvate transaminase; GGT, serum gamma-glutamyl transferase; AP, alkaline phosphatase; CRP, C-reactive protein; ESR 1h, erythrocyte sedimentation rate after 1 hour; ESR 2h, erythrocyte sedimentation rate after 2 hours; Na, sodium; K, potassium; Ca, calcium, Mg, magnesium

### Liver function

Regarding liver function, GOT and GPT levels rose significantly during the course of the fast (fasting intervention: each p<0.001) without difference between groups. The values at baseline and at the end remained within norm ranges (<0.8 μkat/L) increasing for GOT in average from 0.4 to 0.6 μkat/L and GPT from 0.5 to 0.7 μkat/L. GGT levels decreased significantly from a mean of 0.6 to 0.4 μkat/L (fasting intervention: p<0.001). AP showed a significant decrease (fasting intervention: p<0.001), more pronounced in the groups that fasted longer (fasting duration group-by-fasting intervention: p<0.001), with a slight dependence on gender (fasting intervention-by-sex: p = 0.002). The mean values before and after the fast were all in the norm range.

### Inflammatory biomarkers

The inflammatory biomarkers CRP and ESR were analysed. The mean values at baseline and at the end were all in norm range (<5.0 mg/L). CRP raised significantly during fasting (fasting intervention: p<0.001). There was no difference between groups and gender. ESR after 1 and 2 hours decreased significantly (fasting intervention: each p<0.001) and the groups with longer fasting periods displayed stronger reductions (fasting duration group-by-fasting intervention: each p<0.001).

### Renal function and uric acid

Uric acid levels, as well as renal function as reflected by urea and creatinine values, are given in Table 4. A significant increase of blood uric acid was observed (338.1±2.3 to 495.2±4.4 μmol/L) (fasting intervention: p<0.001). The highest uric acid level was measured in F15d. Urea concentrations decreased significantly in all groups (fasting intervention: p<0.001), but the decrease was stronger in the groups with longer fasting periods (fasting duration group-by-fasting intervention: p<0.001). Creatinine levels increased significantly (fasting intervention: p<0.001) with differences between the sexes (fasting intervention-by-sex: p<0.001) but without differences between groups.

### Electrolytes

Sodium concentrations remained in norm range but showed a significant reduction (fasting intervention: p<0.001) from a mean of 140.1±0.1 to 138.7±0.1 mmol/L. Calcium levels increased significantly (fasting intervention: p<0.001), with an effect of groups (fasting duration group-by-fasting intervention: p<0.001) but not of gender. Potassium showed a reduction during fasting (fasting intervention: p = 0.001) and magnesium levels remained stable. All values pre- and post-fasting remained in norm range.

## Discussion

The present prospective observational study systematically investigated for the first time the effects of long periods of Buchinger fasting within a specialized clinic in a cohort of 1422 subjects. The results showed that this type of fasting from 4 to 21 days is safe and well tolerated. Furthermore, it led to enhancement of emotional and physical well-being and improvement of relevant cardiovascular risk factors and subjective health complaints.

Fasting resulted as expected in marked weight loss with reduction of abdominal circumference, which was more pronounced in the groups who fasted longer. Thus it can be assumed that preferentially visceral adipose tissue was reduced with weight loss [40]. Of note, no particular rebound in weight gain has been shown after repeated cycles of Buchinger fasting in a previous study [38, 39].

Blood pressure showed a significant decrease, whereby mean values did not fall below the lower norm range, indicating a floor effect. Accordingly, serious hypotensive complications were not reported. Blood pressure reduction might be triggered by factors such as the increase of parasympathetic activity due to the release of brain-derived neurotrophic factor (BDNF) [2, 41, 42], increased renal Na excretion [43] and enhanced receptor sensitivity of natriuretic peptides and insulin [44]. Earlier studies on zero calorie diets and very-low-calorie diets (VLCD) [45–47]–and more recently in smaller studies on Buchinger fasting [33, 34] and water fasting [48]–also described this blood pressure-reducing effect.

We further found decreases in blood lipid levels following the fasting periods: TG levels as well as TC and LDL-C decreased significantly in all groups. Glucose levels and HbA1c decreased also significantly which points out to a positive effect of fasting on glucoregulation. It was also found in two previous smaller studies using Buchinger fasting [49, 50].

Altogether, the positive impact of periodic Buchinger fasting on the above mentioned parameters suggests a general cardioprotective effect that has also been shown in IF [51].

The continuous increase in emotional as well as physical well-being was evident across all groups of different fasting period lengths. This is an important component to increase compliance and has been reported in earlier studies based on daily mood ratings [26, 52, 53]. Weight loss, especially in obese subjects, is linked with mood improvement in many studies [54]. The G-to-K switch has been shown in IF to lead to enhanced performance in cognition, mood, motor and autonomic nervous system function [2, 55]. In IF and CR this is linked to the release of BDNF [51, 56]. BDNF, associated with neurogenesis and neuron protection, enhances the growth and survival of serotonin neurons [51, 57]. Furthermore, the reduction of insulin and leptin levels appears to act on the hypothalamic-pituitary-adrenal axis, thereby impacting mood positively [58]. Endogenous opioid (β-endorphin) could also contribute to well-being, as documented in a 10-day fasting trial in men [59]. In our study, the reported improvement of a major health complaint, often accompanied by pain relief, could possibly contribute to the increase of well-being. Moreover, it appears likely that a successful completion of a longer fasting period improves the feeling of self-efficacy, thereby enhancing subjective well-being [37]. It has been reported that short fasting periods of two days, as well as alternate-day fasting, are associated with the feeling of hunger [60–62]. This seems to be an obstacle to patient compliance [61, 62]. In contrast, hunger was not reported by 93% of the subjects in our study, which often surprised them positively. This possibly contributed to enhanced well-being.

As expected, we observed a significant increase in urinary ketone bodies excretion up to a maximum level that was similar in all groups. This suggests that in 5 days a plateau was reached. Ketosis and IMS was achieved, although Buchinger fasting provides small quantities of fruit juices and some honey, providing up to 25–35 g carbohydrates/day. Experimental research points to ketosis as the trigger of beneficial effects on brain and neurological diseases [2]. Daily time-restricted feeding causing IMS ameliorates anxiety-like behaviour in mice [63]. IMS enhances also structural and functional synaptic plasticity, cognition and neuronal stress response [64].

In our cohort, no fatal or life-threatening event occurred. Self-reported mild symptoms were observed mainly in the first days and disappeared either spontaneously or with natural remedies. Adverse effects were observed in 0.7% of subjects. Only two subjects had to interrupt the fasting. Adverse effects have also been mentioned in other studies [34, 65].

A recent publication that analysed retrospectively water-only fasting data found relatively more adverse effects, e. g. nausea, presyncope, dyspepsia, vomiting and palpitations [66], which we observed only in single cases. The use of different methodologies to assess and characterise AE limits the comparison with our study, which was prospective. Nevertheless, it could be possible that the supplementation with juices and soups, which slows down initial protein catabolism [67], enhances tolerability by modulating the onset of ketosis.

As already mentioned, we observed a subjective improvement in 85% of cases of a major health complaint. This documents within the limitations of a non-confirmatory study design the therapeutic effectiveness of Buchinger periodic fasting.

To assess further therapeutic effects of fasting, as well as the safety of this procedure, we performed extended laboratory analysis. Within the blood count leukocytes and thrombocytes decreased significantly but not below norm range. In humans after CR as well as in mice fed with cycles of a low calorie fasting mimicking diet, a drop in leukocytes count was followed by an increase in hematopoietic, mesenchymal stem and progenitor cells in the bone marrow. This was associated with the regeneration of all blood cell types and haemoglobin upon refeeding [4, 19]. We cannot extrapolate from our data whether this regeneration applies also to fasting in humans.

The increase in INR values was significant and more marked in the groups who fasted longer. Bleeding time (PTT) was also increased. The increase in INR is well-known [68, 69] and can be relevant for anticoagulated patients. During fasting they should be monitored and their medication adapted.

GOT and GPT levels showed a significant increase within the norm range. GGT instead, dropped significantly and stronger in the groups who fasted longer. A zero-calorie diet in 88 obese people for a duration of up to 35 days showed an increased activity of the GOT and GPT, with a peak in the 3^rd^ fasting week [25]. This moderate increase was explained by the enhanced transamination processes in the course of fasting. An initial overload of liver detoxifying activity could be postulated and does not seem to have been deleterious effects, since GGT levels decreased and physical well-being increased steadily.

CRP values for all groups showed a significant increase within the norm range. A similar mild CRP increase was explained by the transient increase in circulating levels of catecholamines [34]. In a recent study, the modulation of CRP levels was linked to changes in lipid profiles and associated to cardiovascular outcomes [70]. ESR after 1 h and 2 h decreased significantly. Periodic fasting has been shown to clinically improve symptoms of rheumatoid arthritis, suggesting decreased inflammatory processes [71]. IF boosts levels of antioxidants and reduces levels of pro-inflammatory cytokines TNF-α, IL-1β and IL-6 [72]. Serum markers of oxidative damage and inflammation are reduced in asthma patients maintained on an alternate day fast [62]. Moreover, the reduction of abdominal circumference which was significant in our study is also associated with a decrease in pro-inflammatory activity [73].

We observed an increase of uric acid in all groups, with a lower value in F20d than in F15d, suggesting that the peak of uric acid concentration was overwhelmed after 15 days. This corresponds to previous observations from a zero-calorie diet [45, 74]. The increase of uric acid concentrations that exceeded the norm range, interestingly caused only one incidence of gout attack in a 72-year-old obese man, treated for hyperuricemia and gout previous to fasting.

The increased concentration of blood uric acid is due to a slight initial increase in protein catabolism, but above all is related to retention caused by competitive tubular secretion with ketone bodies. The latter are preferentially secreted during fasting.

Uric acid has a known antioxidant activity and is a potent scavenger of free radicals in blood plasma [75, 76]. Given that fasting is the product of a long evolutionary survival strategy, the antioxidant power of uric acid should be taken into consideration.

A significant reduction in urea as well as a significant increase in creatinine was observed, both remaining in norm range. This has been previously demonstrated in obese persons undergoing prolonged periods of a zero-calorie diet [45].

We did not observe any renal dysfunction in our cohort such as has been described in cases of extracellular hypovolemia [77]. This is probably explained by the fact that the subjects were asked to drink 3 L of water per day.

Sodium, potassium, calcium and magnesium were in norm range at the beginning and the end of the fast. They remained stable despite a slight elevation in calcium and slight decrease in sodium levels. Enhanced natriuresis has been described in association with ketosuria in the first phase of fasting, diminishing when ammonium, a metabolite of kidney gluconeogenesis, replaces sodium as accompanying cation [43, 47]. We registered six cases of mild hyponatremia in the course of the fast, with the lowest sodium level of 127 mmol/L. They were all non-serious and were normalized by the administration of sodium chloride.

There are some limitations related to our study. First, this was an observational cohort study with its well-known restrictions regarding interpretation of efficacy. Second, our findings are specific for BWC and may not be applicable for other institutions specialised in fasting, or for persons fasting without medical supervision, or outside of facilities specialized in fasting treatments. Third, data assessment and data entry was not blinded and was performed by the staff of the BWC.

## Conclusions

In conclusion, this one-year observational study demonstrates the safety of a periodic Buchinger fast of between 4 and 21 days, as well as its beneficial effects on health and well-being. Periodic fasting led to marked weight loss and improvements in several cardiovascular risk factors, such as overweight, abdominal circumference and blood pressure. It was accompanied by normalization of numerous blood parameters and led to pronounced improvement of the major health complaint in most participants. Importantly, periodic Buchinger fasting was not linked to relevant perception of hunger. On the contrary, it was subjectively experienced as enjoyable, which is an important factor for compliance.

Further studies should evaluate the long-term specific health-related preventive and therapeutic effects of periodic fasting.

## Supporting information

S1 Table Effect of fasting on clinical parameters, well-being and ketosis. (PDF)

S2 Table Effect of fasting on lipid parameters and glycaemia. (PDF)

S3 Table Raw data of Fig 2. Changes in weight and abdominal circumference according to the length of fast and gender.
(XLSX)

S4 Table Raw data of Fig 3. Changes in systolic and diastolic blood pressure according to the fasting length.
(XLSX)

S5 Table Raw data of Fig 4. Changes in emotional and physical well-being during fasting.
(XLSX)

S6 Table Raw data of Fig 5. Fasting-induced changes in lipid metabolism.
(XLSX)

S7 Table Raw data of Fig 6. Changes of blood glucose and glycated haemoglobin.
(XLSX)

S8 Table Raw data of Table 1. Baseline characteristics of the subjects.
(XLSX)

S9 Table Raw data of Table 2. Mild symptoms and adverse effects.
(XLSX)

S10 Table Raw data of Table 3. Blood cells pre- and post-fasting.
(XLSX)

S11 Table Raw data of Table 4. Blood parameters pre- and post-fasting.
(XLSX)

S12 Table Raw data of S1 Table. Effect of fasting on clinical parameters, well-being and ketosis.
(XLSX)

S13 Table Raw data of S1 Fig. Occurrence of self-reported mild symptoms during fasting.
(XLSX)

S14 Table Raw data of S2 Fig. Evolution of a pre-existing health complaint.
(XLSX)

S15 Table Raw data of S4 Table. Effect of fasting on lipid parameters and glycaemia.
(XLSX)

S1 Fig Occurrence of self-reported mild symptoms during fasting. (PDF)

S2 Fig Evolution of a pre-existing health complaint. A subgroup of 404 subjects indicated to have a major health complaint previous to the fasting.
(PDF)

S1 Text STROBE checklist. (PDF)

S2 Text Study protocol in German. (PDF)

S3 Text Study protocol translated to English. (PDF)

S4 Text Questionnaires in German. (PDF)

S5 Text Questionnaires in English. (PDF)

S6 Text Questionnaires in French. (PDF)
