# Supplementary material for: Safety, health improvement and well-being during a 4 to 21-day fasting period in an observational study including 1422 subjects
Source: PLoS One. 2019 Jan 2;14(1):e0209353. doi: 10.1371/journal.pone.0209353 (PMC6314618; doi:10.1371/journal.pone.0209353)
Supplement: S1 Table — (PDF) [file pone.0209353.s001.pdf]

S1 Table: Effect of fasting on clinical parameters, well-being and ketosis.

	**All** (n= 1422)		**F5d 5±2 d**		**F10d 10±2 d**		**F15d 15±2 d**		**F20d 20±2 d**		**p-values**				
	pre	post	pre	post	pre	post	pre	post	pre	post	fasting intervention	fasting duration group	sex	fasting duration group-by-fasting intervention	fasting intervention -by-sex
Weight, kg	82.0±0.5	77.9±0.5	79.3±0.8	76.1±0.7	82.7±0.9	78.3±0.8	86.6±1.6	80.5±1.4	96.7±4.0	89.6±3.7	<0.001	<0.001	<0.001	<0.001	<0.001
BMI, kg/m²	28.2±0.2	26.7±0.1	27.2±0.2	26.1±0.2	28.5±0.3	27.0±0.2	29.7±0.4	27.5±0.4	33.6±1.1	31.0±1.1	<0.001	<0.001	0.001	<0.001	<0.001
Waist, cm	94.0±0.4	88.0±0.5	91.3±0.6	86.4±0.7	94.8±0.7	88.6±0.7	98.3±1.2	89.4±1.3	106.3±2.8	96.9±3.1	<0.001	<0.001	0.024	<0.001	<0.001
SBP, mmHg	130.6±0.6	120.6±0.4	129.0±0.8	122.0±0.6	130.3±0.9	119.2±0.7	136.0±1.5	119.9±1.2	134.2±3.4	118.0±1.8	<0.001	<0.001	0.007	<0.001	–
DBP, mmHg	83.2±0.3	77.7±0.3	82.5±0.5	78.5±0.4	83.2±0.5	77.0±0.4	84.7±0.8	77.0±0.7	86.3±1.9	78.3±1.5	<0.001	<0.001	<0.001	<0.001	–
Heart rate, beats/min	69.4±0.3	71.3±0.3	69.3±0.4	72.0±0.4	69.2±0.5	70.6±0.5	70.2±0.7	71.0±0.8	69.6±1.5	68.2±2.0	0.04	0.003	0.003	0.007	–
EWB, score 0-10	6.2±0.1	7.6±0.1	6.3±0.1	7.6±0.1	6.3±0.1	7.5±0.1	5.7±0.2	7.7±0.1	6.6±0.4	8.0±0.3	<0.001	0.02	0.91	<0.001	–
PWB, score 0-10	5.8±0.1	7.4±0.1	5.8±0.1	7.3±0.1	5.8±0.1	7.3±0.1	5.5±0.2	7.6±0.1	5.9±0.4	7.9±0.3	<0.001	0.01	0.34	0.007	–
Acetoacetic acid, mg/dL	2.5±0.3	50.2±1.2	2.6±0.4	50.8±1.7	2.6±0.4	49.6±2.0	2.3±0.7	49.5±3.2	2.6±1.3	51.8±8.6	<0.001	0.81	<0.001	–	<0.001

Values are shown as mean±SEM for all of the groups with different fasting lengths. P-values were calculated for the effects of fasting intervention as well as the effects of the fasting length (fasting duration group) and gender (sex). Interactions between fasting intervention by fasting duration group (fasting duration group-by-fasting intervention) and fasting intervention by gender (fasting intervention-by-sex) are shown.

SBP, systolic blood pressure; DBP, diastolic blood pressure; EWB, emotional well-being; PWB, physical well-being.
