# Supplementary material for: Safety, health improvement and well-being during a 4 to 21-day fasting period in an observational study including 1422 subjects
Source: PLoS One. 2019 Jan 2;14(1):e0209353. doi: 10.1371/journal.pone.0209353 (PMC6314618; doi:10.1371/journal.pone.0209353)
Supplement: S2 Table — (PDF) [file pone.0209353.s002.pdf]

S2 Table: Effect of fasting on lipid parameters and glycaemia.

	**All** (n= 1422)		**F5d 5±2 d**		**F10d 10±2 d**		**F15d 15±2 d**		**F20d 20±2 d**		**p-values**				
	pre	post	pre	post	pre	post	pre	post	pre	post	fasting intervention	fasting duration group	sex	fasting duration group-by-fasting intervention	fasting intervention -by-sex
TG, mmol/l	1.5±0.0	1.1±0.0	1.5±0.0	1.1±0.0	1.6±0.0	1.1±0.0	1.6±0.1	1.1±0.0	1.5±0.1	1.2±0.0	<0.001	0.004	0.72	0.001	<0.001
TC, mmol/l	5.6±0.0	4.9±0.0	5.5±0.0	5.2±0.0	5.6±0.0	4.9±0.0	5.6±0.1	4.4±0.1	5.7±0.2	4.5±0.2	<0.001	<0.001	0.67	<0.001	–
HDL-C, mmol/l	1.5±0.0	1.3±0.0	1.6±0.0	1.4±0.0	1.5±0.0	1.3±0.0	1.5±0.0	1.2±0.0	1.5±0.1	1.1±0.0	<0.001	<0.001	0.36	<0.001	<0.001
LDL-C, mmol/l	3.5±0.0	3.2±0.0	3.4±0.0	3.4±0.0	3.5±0.0	3.1±0.0	3.6±0.1	2.7±0.1	3.8±0.2	2.8±0.2	<0.001	<0.001	0.19	<0.001	–
LDL/HDL ratio	2.6±0.1	2.6±0.0	2.7±0.3	2.7±0.0	2.5±0.0	2.6±0.1	2.5±0.1	2.4±0.1	2.7±0.1	2.6±0.2	0.66	0.003	<0.001	–	–
Glucose, mmol/l	5.4±0.0	4.7±0.0	5.3±0.0	4.6±0.0	5.4±0.1	4.7±0.1	5.6±0.1	4.7±0.1	5.5±0.2	4.9±0.2	<0.001	0.68	0.12	0.03	<0.001
HbA1c, mmol/mol	36.0±0.2	34.3±0.2	35.5±0.3	34.2±0.3	36.1±0.3	34.4±0.3	36.7±0.4	34.5±0.4	37.6±1.3	34.9±1.0	<0.001	<0.001	0.67	<0.001	0.03

Values are shown as mean±SEM for all of the groups with different fasting lengths. P-values were calculated for the effects of fasting intervention as well as the effects of the fasting length (fasting duration group) and gender (sex). Interactions between fasting intervention by fasting duration group (fasting duration group-by-fasting intervention) and fasting intervention by gender (fasting intervention-by-sex) are shown.

TG, triglyceride; TC, total cholesterol; HDL-C, high-density-lipoprotein; LDL-C, low-density-lipoprotein; HbA1c, glycated haemoglobin.
