# Supplementary figures and images for: Safety, health improvement and well-being during a 4 to 21-day fasting period in an observational study including 1422 subjects
Source: PLoS One. 2019 Jan 2;14(1):e0209353. doi: 10.1371/journal.pone.0209353 (PMC6314618; doi:10.1371/journal.pone.0209353)

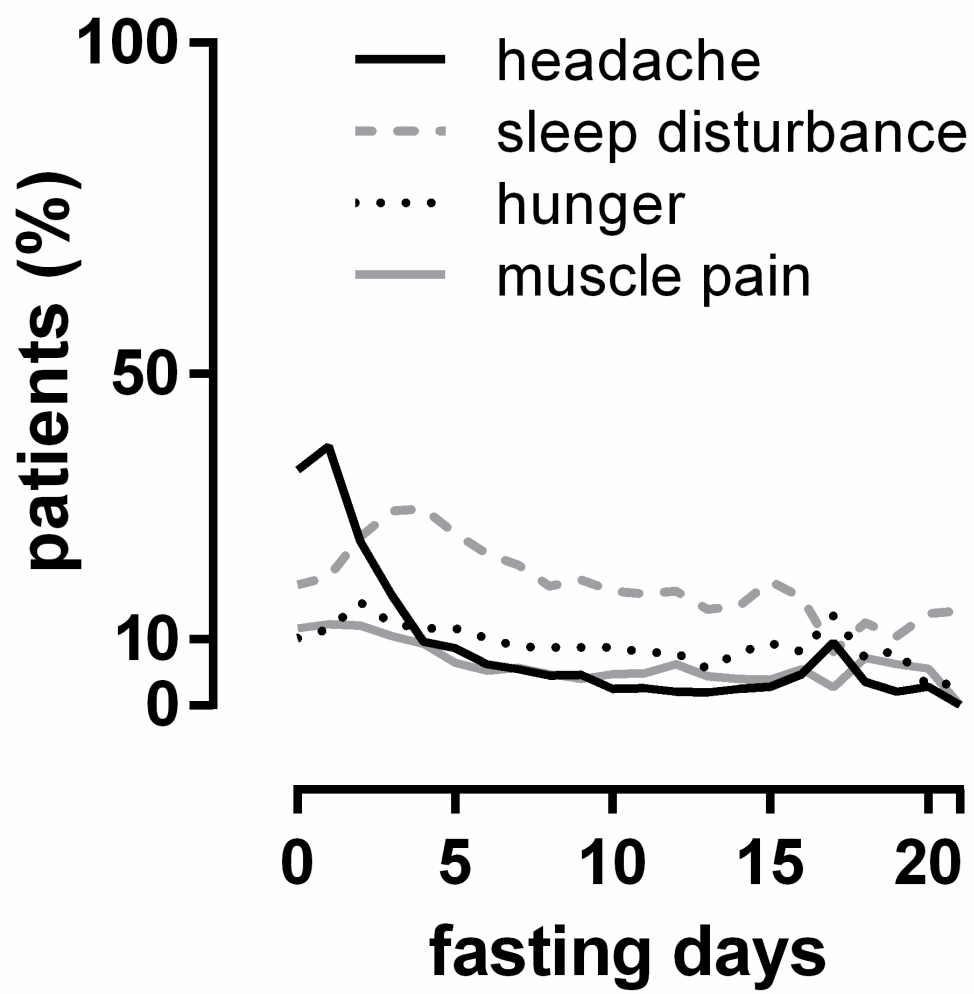

S1\_Fig. Occurrence of self-reported mild symptoms during fasting.

Supplement: S1 Fig — (PDF) [file pone.0209353.s016.pdf]
