# Supplementary material for: Safety, health improvement and well-being during a 4 to 21-day fasting period in an observational study including 1422 subjects
Source: PLoS One. 2019 Jan 2;14(1):e0209353. doi: 10.1371/journal.pone.0209353 (PMC6314618; doi:10.1371/journal.pone.0209353)
Supplement: S2 Fig — A subgroup of 404 subjects indicated to have a major health complaint previous to the fasting. (PDF) [file pone.0209353.s017.pdf]

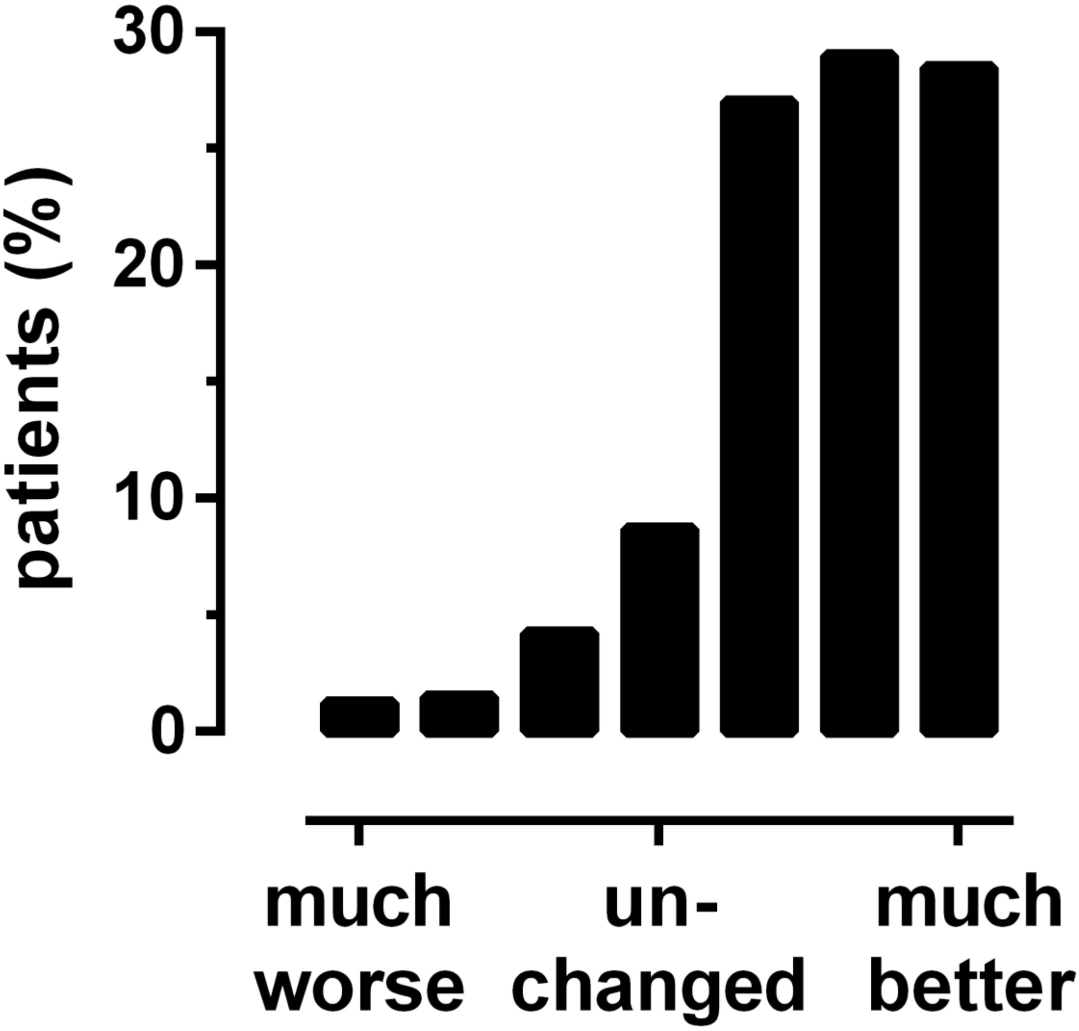

S2_Fig. Evolution of a pre-existing health complaint. A subgroup of 404 subjects indicated to have a major health complaint previous to the fasting.
