# Supplementary material for: Safety, health improvement and well-being during a 4 to 21-day fasting period in an observational study including 1422 subjects
Source: PLoS One. 2019 Jan 2;14(1):e0209353. doi: 10.1371/journal.pone.0209353 (PMC6314618; doi:10.1371/journal.pone.0209353)
Supplement: S1 Text — (PDF) [file pone.0209353.s018.pdf]

S1 Text. STROBE Checklist.

STROBE Statement—checklist of items that should be included in reports of observational studies

	Item No	Recommendation
**Title and abstract**	1	(*a*) Indicate the study's design with a commonly used term in the title or the abstract<br>The title includes the study design: "observational study"
		(*b*) Provide in the abstract an informative and balanced summary of what was done and what was found<br>The abstract describes background, methods, results and conclusion
**Introduction**		
Background/rationale	2	Explain the scientific background and rationale for the investigation being reported<br>This is outlined in paragraph 1 and 2 in the introduction
Objectives	3	State specific objectives, including any prespecified hypotheses<br>This is outlined in paragraph 3 of the introduction
**Methods**		
Study design	4	Present key elements of study design early in the paper<br>The key elements of the study design are presented in the abstract
Setting	5	Describe the setting, locations, and relevant dates, including periods of recruitment, exposure, follow-up, and data collection<br>This is outlined in the sections participants, fasting program and measurements
Participants	6	(*a*) *Cohort study*—Give the eligibility criteria, and the sources and methods of selection of participants. Describe methods of follow-up<br>Information about inclusion and exclusion criteria is provided in the section participants and the flow chart enclosed as supplementary information
		(*b*) *Cohort study*—For matched studies, give matching criteria and number of exposed and unexposed<br>Not applicable
Variables	7	Clearly define all outcomes, exposures, predictors, potential confounders, and effect modifiers. Give diagnostic criteria, if applicable<br>This information is provided in the methods section
Data sources/ measurement	8*	For each variable of interest, give sources of data and details of methods of assessment (measurement). Describe comparability of assessment methods if there is more than one group<br>This information is provided in the methods section
Bias	9	Describe any efforts to address potential sources of bias<br>The participants had different fasting lengths. To address this, we clustered the participants into 4 groups with similar lengths of 5, 10, 15 and 20 days. Other possible bias are documented at the end of the discussion as "limitation"
Study size	10	Explain how the study size was arrived at<br>The flow chart in the supplementary information explains this
Quantitative variables	11	Explain how quantitative variables were handled in the analyses. If applicable, describe which groupings were chosen and why

		This is described in the methods section.
Statistical methods	12	(*a*) Describe all statistical methods, including those used to control for confounding<br>The statistical analysis is described in the methods
		(*b*) Describe any methods used to examine subgroups and interactions<br>The statistical analysis is described in the methods
		(*c*) Explain how missing data were addressed<br>Subjects who did not complete the daily questionnaire were not considered in the data analysis concerning self-reported data, see measurements
		(*d*) *Cohort study*—If applicable, explain how loss to follow-up was addressed<br>Imputations were not performed<br>*Case-control study*—If applicable, explain how matching of cases and controls was addressed<br>*Cross-sectional study*—If applicable, describe analytical methods taking account of sampling strategy
		(*e*) Describe any sensitivity analyses<br>Not applicable
**Results**		
Participants	13*	(a) Report numbers of individuals at each stage of study—eg numbers potentially eligible, examined for eligibility, confirmed eligible, included in the study, completing follow-up, and analysed<br>The flow chart in the supplementary information provides this information
		(b) Give reasons for non-participation at each stage<br>The flow chart in the supplementary information provides this information
		(c) Consider use of a flow diagram<br>The flow chart is given in the supplementary information
Descriptive data	14*	(a) Give characteristics of study participants (eg demographic, clinical, social) and information on exposures and potential confounders<br>Information about the characteristics of the study participants can be found in the section participants and in Table 1
		(b) Indicate number of participants with missing data for each variable of interest<br>Missing self-reported data is indicated in the measurements section
		(c) *Cohort study*—Summarise follow-up time (eg, average and total amount)<br>Follow-ups were defined by the reported lengths of the clinic stay
Outcome data	15*	*Cohort study*—Report numbers of outcome events or summary measures over time<br>The numbers of outcomes are described in the results section
		Case-control study—Report numbers in each exposure category, or summary measures of exposure<br>Not applicable
		Cross-sectional study—Report numbers of outcome events or summary measures<br>Not applicable
Main results	16	(*a*) Give unadjusted estimates and, if applicable, confounder-adjusted estimates and their precision (eg, 95% confidence interval). Make clear which confounders were adjusted for and why they were included<br>The Tables 1, 3, 4 and the supplementary Tables S1 and S2 provide this information
		(*b*) Report category boundaries when continuous variables were categorized<br>Not relevant

		(*c*) If relevant, consider translating estimates of relative risk into absolute risk for a meaningful time period<br>Not relevant
Other analyses	17	Report other analyses done—eg analyses of subgroups and interactions, and sensitivity analyses<br>No further analysis were done
**Discussion**		
Key results	18	Summarise key results with reference to study objectives<br>Key results of the study in relation to the study objectives are summarized in the beginning of the discussion
Limitations	19	Discuss limitations of the study, taking into account sources of potential bias or imprecision. Discuss both direction and magnitude of any potential bias<br>Limitations of the study are discussed at the end of the discussion
Interpretation	20	Give a cautious overall interpretation of results considering objectives, limitations, multiplicity of analyses, results from similar studies, and other relevant evidence<br>The interpretation can be found in the discussion section
Generalisability	21	Discuss the generalisability (external validity) of the study results<br>This is described in the last paragraph of the discussion
**Other information**		
Funding	22	Give the source of funding and the role of the funders for the present study and, if applicable, for the original study on which the present article is based<br>Information about funding and the role of the funders is provided in the online submission system of PLOS ONE as follows:<br>The study was financed by Amplius GmbH, Überlingen, Germany. This company has the task to develop a research department for the Buchinger Wilhelmi Clinics Überlingen and Marbella who are the funders. The funders had no role in study design, data collection and analysis, decision to publish, or preparation of the manuscript. No additional external funding received for this study.

*Give information separately for cases and controls in case-control studies and, if applicable, for exposed and unexposed groups in cohort and cross-sectional studies.

Note: An Explanation and Elaboration article discusses each checklist item and gives methodological background and published examples of transparent reporting. The STROBE checklist is best used in conjunction with this article (freely available on the Web sites of PLoS Medicine at http://www.plosmedicine.org/, Annals of Internal Medicine at http://www.annals.org/, and Epidemiology at http://www.epidem.com/). Information on the STROBE Initiative is available at www.strobe-statement.org.
