# Supplementary material for: Safety, health improvement and well-being during a 4 to 21-day fasting period in an observational study including 1422 subjects
Source: PLoS One. 2019 Jan 2;14(1):e0209353. doi: 10.1371/journal.pone.0209353 (PMC6314618; doi:10.1371/journal.pone.0209353)
Supplement: S2 Text — (PDF) [file pone.0209353.s019.pdf]

S2 Text. Study protocol in German

Antrag auf Beratung durch die Ethikkommission zur Durchführung eines medizinisch-wissenschaftlichen Vorhabens, welches nicht die klinische Prüfung eines Arzneimittels beinhaltet

1. Titel der Studie	Wissenschaftliche Begleitdokumentation der klinischen Ernährungstherapie mit dem Buchinger Wilhelmi Programm® (modifiziertes Fasten, kalorische Restriktion und vegetarische Kost)
2. Ethikkommissions -Antragsnummer	
3. Entscheidungen anderer Ethikkommissionen in derselben Sache	-----
4. Gegenstand der Studie und ihre Ziele; Angabe der Hypothesen, getrennt in Haupt- und Sekundärhypothesen sowie der klinischen Parameter (primäre und sekundäre Endpunkte), anhand derer die Hypothesen geprüft werden	Ziel der Studie ist die prospektive wissenschaftliche Dokumentation der klinischen Effekte einer stationären/klinischen Therapie unter schwerpunktmäßigem Einsatz des modifizierten Fastens und der kalorischen Restriktion durch ein Prä/Post- und Gruppenvergleich sowie ein selektives schriftliches Follow-up bei konsekutiven regulären Patienten (Outcome Forschung)<br><br><u>Explorative Zielparameter:</u><br><br>Zu Beginn, während und am Ende des stationären Aufenthaltes sowie bis zu 12 Monate nachfolgend per Emailing erfolgen die klinikregulären Erhebungen der Zielparameter und die Analyse der Verlaufsänderungen der folgenden Studienendpunkte:<br>- Körpergröße<br>- Ruhe-Blutdruck, sys./ diast.<br>- Klinisches Labor<br>- Gewicht<br>- Bauchumfang<br>- Ruhe-Pulsfrequenz<br>- Stimmung (Profile of Mood States), Befinden (VAS), Stress (Cohen Skala), Lebensqualität (WHO-5)<br>- Subjektives Stresserleben<br>- Quantifizierung (subjektiver Selbstbericht) von Verhaltensfaktoren: Nikotin, Alkohol, Bewegungsmangel, Kaffee, Medienkonsum<br>- Subjektive Stärke der Hauptbeschwerde<br>- Unerwünschte Wirkungen

	Daneben erfolgt eine systematisierte Dokumentation der Medikation, der Haupt- und Nebendiagnosen und der Ernährungsanamnese und Kostverordnung.<br><br>Im Anschluss an den Aufenthalt werden mittels Emailing die Verhaltensfaktoren und die subjektiv empfundenen Wirkungen des Fastens bzw. der kalorischen Restriktion abgefragt zur Erfassung der Nachhaltigkeit.
5. Erläuterung der Bedeutung der Studie	Das Heilfasten bzw. therapeutische Fasten wird seit vielen Jahrzehnten in der Naturheilkunde und Integrativen Medizin klinisch erfolgreich in der Behandlung chronisch internistischer Erkrankungen und von Schmerzsyndromen eingesetzt (1,11,17,18,21). Insbesondere bei chronischen Erkrankungen aus dem Bereich der rheumatischen, entzündlichen und metabolischen Indikationen wird die Fastentherapie bei wachsender Patientennachfrage in spezialisierten klinischen Einrichtungen (Fastenkliniken) angewandt. Darüber hinaus ist die Fastentherapie im Bereich der klassischen Naturheilkunde und der naturheilkundlichen Komplexbehandlung ein fester Bestandteil der Therapie Kernmodule.<br><br>Innerhalb der verschiedenen historisch gewachsenen Formen des Fastens hat sich das Fastenprogramm nach der Methode Buchinger Wilhelmi weltweit als die häufigste angewandte Methode etabliert (25). Hierbei wir über eine zeitlich definierte Periode von mindestens 10 Tagen eine subtotale kalorische Restriktion mit einer täglichen Kalorienzufuhr (200-400kcal/Tag) in Form von flüssigen Bestandteilen durchgeführt und begleitet durch unterstützende Maßnahmen eines gesundheitsfördernden Lebensstilprogrammes mit Elementen wie Bewegungstherapie, Manuelle Verfahren, Stress-Reduktion und Hydro-Balneotherapie (25).<br><br>In frühen randomisierten Studien und einem systematic review konnte die Wirksamkeit der stationären Fastentherapie für das Indikationsgebiet der rheumatoiden Arthritis mit einer 1a- Evidenz belegt werden (8,21). Für die weiteren Indikationen bestehen vorwiegend empirische Belege oder Daten aus observationellen oder prospektiven unkontrollierten Studien (5,6,16-19,22).In den letzten Jahren entwickelte sich eine umfangreiche grundlagenwissenschaftliche Forschungsaktivität im Gebiet der kalorischen

	Restriktion und des intermittierenden Fastens. Hierbei konnte eine Vielzahl von günstigen tierexperimentellen Befunden durch definierte Fastenperioden belegt werden, u.a. Absenkungen von Insulin, IGF-1, Anstiege von Adiponektinen, Insulinsensitivität, neurotrophen Faktoren und in längerer Beobachtungsdauer auch die Abnahme der Inzidenz von kardiovaskulären, inflammatorischen und metabolischen sowie unlängst auch onkologischen Erkrankungen bei verschiedensten Tierspezies (3,4,9,10,12,15,23,24).<br><br>Vor diesem Hintergrund ist es wissenschaftlich sinnvoll und geboten die klinischen Effekte der Fastentherapie systematisch in größeren behandelten Populationen zu dokumentieren, insbesondere um weitere methodische Informationen für zukünftige klinische Studien zu gewinnen sowie Subpopulationen und Responder zu analysieren. Mit der beantragten Studie soll prospektiv erstmals eine größere Population (n>1000) systematisch dokumentiert und analysiert werden.
6. Welche der folgenden Bestimmungen finden Anwendung<br>• Medizinproduktegesetz -gemäß § 20 MPG (Gerät besitzt nicht die Konformitätserklärung oder diese liegt vor und es wird eine andere Indikation geprüft oder es werden zusätzlich invasive oder andere belastende Untersuchungen durchgeführt) **oder** -gemäß § 23 MPG ?<br>• Strahlenschutzverordnung § 23<br>• Röntgenverordnung § 28 a<br>• Gentechnikgesetz<br>• Datenschutzgesetze	Datenschutzgesetze
7. Ggf.: Bezeichnung und Charakterisierung der Prüfprodukte (z.B. Geräte bei MPG-Studien; bitte Anlagen beifügen)	----
8. wesentliche Ergebnisse der vorklinischen Tests oder Gründe für die Nichtdurchführung derselben	Für die physiologischen Wirkungen der kalorischen Restriktion und des intermittierenden Fastens besteht eine umfassende tierexperimentelle Datenlage, (3,12,14) die auf die präventiv und/oder therapeutischen gesundheitsfördernden Effekte für eine Vielzahl von degenerativen, entzündlichen, kardiovaskulären und onkologischen Erkrankungen hinweist.
9. Wesentlicher Inhalt und Ergebnisse der vorangegangenen Studien/ Anwendungen der in der Studie zu prüfenden Produkte	Prolongiertes Fasten und intermittierendes Fasten haben in zahlreichen experimentellen und einigen klinischen Studien günstige Effekte auf die kardiometabolische, gluko-regulatorische, und neuroendokrino-immunologische Funktion gezeigt.

	Hierbei stehen ausgeprägte Absenkungen von Insulin, Leptin, IGF-1 u.a. [3,12,14,15] und Erhöhungen von neurotrophen Faktoren, Adiponektin, natriuretischen Peptiden als Mediatoren der potentiellen günstigen Wirkungen im Vordergrund der aktuellen Diskussion. [13,17,24].<br>In den randomisierten Studien zeigten sich klinisch relevante Effekte auf den Verlauf der rheumatoiden Arthritis (8,21). In kontrollierten nicht-randomisierten sowie unkontrollierten Interventionsstudien fanden sich günstige Wirkungen auf den Blutdruck, die Insulinsensitivität, den Verlauf chronischer Schmerzen des Bewegungsapparates, auf psychische Parameter sowie in einer kürzlich abgeschlossenem RCT (in Publikation) auf die Lebensqualität bei Multipler Sklerose (2,6,11,17,16,22). In den bisherigen Studien mit längerer Beobachtungsdauer fanden sich eine verbesserte Compliance mit Maßgaben eines gesundheitsfördernden Lebensstil und gesunder Ernährung sowie keine ernsthaften unerwünschten Wirkungen des Fastens (18).
10. Beschreibung der vorgesehenen Maßnahmen/Untersuchungsmethoden und eventuelle Abweichungen von den in der med. Praxis üblichen Maßnahmen/Untersuchungen (was ist „Routine“, was wird davon abweichend in der Studie gemacht?)	Es werden reguläre konsekutive stationäre bzw. klinische Patienten der Klinik Buchinger Wilhelmi in Überlingen und der Abteilung für Naturheilkunde am Immanuel Krankenhaus Berlin für die Studienteilnahme angefragt.<br><br>Die Behandlungsdauer wird in der Regel zwischen 10 Tagen bis 4 Wochen umfassen.<br>Hierbei erfolgt kein Einfluss auf die therapeutischen Maßnahmen im Rahmen der Studie. Die Patienten erhalten ernährungstherapeutisch nach Präferenz und/oder Indikation ein therapeutisches Fasten nach der Buchinger Methode oder eine spezifizierte kalorisch reduzierte Diät (800/1200kcal) bzw. bei bestehenden Kontraindikationen vollkalorische Ernährung (Einzelfälle) und ein begleitendes Lebensstil und Naturheilkunde Programm.<br><br>Die Anfrage zur Studienteilnahme erfolgt beim Aufnahmegespräch mit dem Arzt/Ärztin.<br>Für die Erhebungen im Rahmen der wissenschaftlichen Dokumentation werden die im Rahmen des üblichen klinischen Vorgehens vorgesehenen und durchgeführten diagnostischen Maßnahmen und Befunderhebungen verwendet. Diese umfassen die bis zu täglichen Messungen von Blutdruck, Puls, Gewicht, Bauchumfang sowie das reguläre klinische Standardlabor, in der Regel zu Beginn und am Ende des Aufenthaltes. Darüber hinaus erfolgen im Rahmen des regulären Klinikvorgehen die Dokumentationen und Einschätzungen des selbstbewerteten physischen und psychologischen Gesamtzustandes, der Lebensstil und Ernährungsgewohnheiten vorher und während des Aufenthaltes, der Medikation, der allgemeinen

	Wirksamkeitseinschätzung in Bezug auf die Hauptbeschwerde(n) sowie die allgemeine Zufriedenheit. Täglich werden mögliche unerwünschte Wirkungen im Zusammenhang mit der Fastentherapie oder der anderweitigen Ernährungstherapie dokumentiert.<br>Es erfolgen keine weiteren Maßnahmen, die von der Routine abweichen.
11. Bewertung und Abwägung der vorhersehbaren Risiken und Nachteile der Studienteilnahme gegenüber dem erwarteten Nutzen für die Studienteilnehmer und zukünftig erkrankte Personen (Nutzen-Risiko-Abwägung)	Studienteilnehmer können individuell profitieren während ernsthafte Risiken für das zu evaluierende Verfahren nicht bekannt sind. Zusammenfassend ergibt sich eine positive Nutzen-Risiko Bewertung.
a. Voraussehbarer therapeutischer Nutzen für die Studienteilnehmer (**individueller Nutzen** für den einzelnen Patienten)	Alle Teilnehmer erhalten eine reguläre potentiell wirksame Therapie (Klinikaufenthalt, therapeutisches Fasten oder kalorisch restriktive Vollwerternährung). Im Rahmen der vorliegenden Studie erfolgt eine systematisierte sorgfältige klinische Dokumentation. Es ergibt sich somit ein voraussehbarer möglicher Nutzen durch die Studienteilnahme.
b. Voraussehbarer medizinischer Nutzen für zukünftig erkrankte Personen **(Gruppennutzen)**	Die Ergebnisse der Studie können zur Verbesserung der medizinischen Versorgung von Patienten mit der Fastentherapie beitragen. Damit ist ein allgemeiner Nutzen für zukünftige Patienten durch die Studienergebnisse gegeben.
c. **Risiken** und Belastungen für die Studienteilnehmer (alle im Einzelnen auflisten)	Alle Erhebungen gehen nicht über die auch sonst vorgesehene klinische Routine mit Ausnahme weniger einzelner Fragen der Selbsteinschätzung hinaus. Insgesamt ergibt sich kein relevantes gesundheitliches Risiko durch die Studienteilnahme.
12. Maßnahmen zur Risikobeherrschung	Teilnehmer werden angehalten alle möglichen unerwünschten Ereignisse (UE) umgehend zu melden. UE werden vom Studienleiter und Prüfärzten bewertet und der Studienfortgang entsprechend überwacht.
13. Abbruchkriterien	Jedes UE wird vom Prüfarzt hinsichtlich seiner Schwere und des möglichen Zusammenhangs mit der Prüftherapie beurteilt. Schwerwiegende UE und unerwünschte Wirkungen müssen vom Prüfarzt innerhalb von 24 Stunden per Telefon, Telefax oder Telegramm dem Leiter der Klinischen Prüfung mitgeteilt werden.<br>Nimmt der Patient seine Einverständniserklärung zurück, so muss der Prüfarzt für ihn die Studie abbrechen. Dieses wird auf den Dokumentationsbögen vermerkt.<br>Die Studie kann vorzeitig abgebrochen werden, wenn erkennbar ist, dass die Studie nicht die genannten Anforderungen erfüllen kann. Hierzu zählen:<br>- es treten schwerwiegende Protokollverletzungen auf<br>- die Dokumentationsbögen werden mangelhaft oder vorsätzlich falsch ausgefüllt<br>- gesetzliche oder ethische Bestimmungen werden nicht eingehalten<br>- ein Abbruch aus obengenannten Gründen ist nur in

	Übereinstimmung zwischen Studienleiter und Prüfärzten möglich.<br><br>Treten schwerwiegende UE oder gravierende NW auf oder häufen sich nicht-schwerwiegende UE, so kann der Studienleiter in alleiniger Entscheidung die Studie abbrechen. Die Studie muss beendet werden, wenn aufgrund von unerwünschten Ereignissen die Sicherheit der Therapie in Frage zu stellen ist.
14. Anzahl, Alter und Geschlecht der betroffenen Personen	Die Studien wird in zwei Zentren durchgeführt, in denen gemischte Patientengruppen behandelt werden<br>1. Klinik Buchinger Wilhelmi in Überlingen<br>2. Immanuel Krankenhaus Berlin, Abt. für Naturheilkunde /Stiftungsprofessur für Naturheilkunde der Charité Berlin<br>An diesen Zentren werden jährlich (n=2000 und n=1000 Patienten mit Fastentherapie der kalorischer Restriktion stationär behandelt. Zumeist ist der Anteil von Frauen ggü. Männern leicht erhöht, die Altersspanne der fastenden Patienten geht durch alle Altersstufen mit einem Schwerpunkt zwischen 45-65 Jahren und einem Spektrum von 18 -99 Jahren.
15. Statistische Planung und Angabe sowie biometrische Begründung der Fallzahl und Unterschrift des/der Statistikers/Statistikerin	Die statistische Analyse vergleicht explorativ die Prä- zu Post Daten aller klinischen Parameter und Inventare zur Einschätzung des Fastens im Intragruppenvergleich. Weiter erfolgt ein Gruppenvergleich von Patienten, die fasten versus Patienten, die eine moderate kalorische Restriktion erhalten. Die Gruppenvergleiche erfolgen explorativ mit ANCOVA.<br>Schließlich erfolgt bei allen Patienten ein Emailing Follow-up per Mail 7 Tage, 3 und 12 Monate nach Entlassung. Die statistische Aufarbeitung erfolgt an der Stiftungsprofessur für klinische Naturheilkunde, Institut für Sozialmedizin, Epidemiologie und Gesundheitsökonomie.<br><br>Fallzahl: Eine Fallzahlschätzung ist aufgrund des Charakters der Studie i.S. einer Outcome –Forschung mit Prä- zu Post Vergleichen nicht notwendig. Ziel ist der Einschluss von insgesamt mindestens n>1000 Patienten in einem Zeitraum von 2 Jahren.
16.<br>a. Darlegung und ggf. Erläuterung der **Ein- und Ausschlusskriterien**	Einschlusskriterien:<br><br>- Alter 18-99 Jahre<br>- Beginnende (erste 24h) stationäre Behandlung bzw. Klinikaufenthalt an einem der beiden Zentren in Überlingen oder Berlin<br>- Vorliegende schriftliche Einverständniserklärung<br><br>Ausschlusskriterien:<br>-Keine ausreichende sprachliche Verständigung

	möglich<br>-Demenz oder anderweitig kognitiv stark beeinträchtigende Erkrankung<br>- Bestehende Schwangerschaft oder Stillzeit<br>- Teilnahme an anderer Studie
b. **Teilnehmerinformation** (wer diese mündlich erteilt und Angabe, wie viel Zeit zwischen Aufklärung und Einwilligung verbleibt, ansonsten Verweis auf deren Inhalt als Anlage möglich)	- Klinikärzte geben persönlich die Erstinformation und eine schriftliche Teilnehmerinformation wird ausgehändigt. Zwischen erster Aufklärung und Teilnahmeeinwilligung verbleiben 24 h.
c. **Einwilligungserklärung** (Verweis auf deren Inhalt als Anlage möglich)	s. Anlage
d. Ggf. **Information und Einwilligung des gesetzlichen Vertreters** (ggf. auch Beschreibung des Verfahrens zur Einrichtung einer gerichtlichen Betreuung)	---
17. Maßnahmen zur Gewinnung von Studienteilnehmern (Aushang ?, Zeitungsannoncen? Etc.)	Die Patienten werden in den beiden Zentren in Überlingen und Berlin im Rahmen des stationären Aufnahmegespräches über die Möglichkeit der Studienteilnahme informiert.
18. Ggf.: **Grund für die Einbeziehung und Darlegung des therapeutischen Nutzens für Personen, die minderjährig und/oder nicht einwilligungsfähig sind.**	--
19. Beziehung zwischen Studienteilnehmer und Studienarzt/-ärztin (Ist der Studienarzt zugleich der behandelnde Arzt?)	In Einzelfällen möglich an der Klinik Buchinger Wilhelmi.
20. Erklärung zur Einbeziehung möglicherweise vom Sponsor oder Studienarzt abhängiger Personen	--
21. Maßnahmen, die eine Feststellung zulassen, ob ein Studienteilnehmer an mehreren Studien zugleich oder vor Ablauf einer in der vorangegangenen Studie festgelegten Frist teilnimmt.	Alle Patienten werden explizit nach einer anderweitigen Studienteilnahme befragt und darauf hingewiesen, dass eine gleichzeitige Teilnahme an anderen klinischen Studien im Studienzeitraum nicht möglich ist.
22. Ggf.: Honorierung bzw. Kostenerstattung der Studienteilnehmer (Höhe, wofür soll gezahlt werden ?)	---
23. Ggf.: Plan für die Weiterbehandlung und medizinische Betreuung der betroffenen Personen nach dem Ende der Studie	---
24. Ggf.: Versicherung der Studienteilnehmer (Versicherungsbestätigung und Versicherungsbedingungen, Versicherer, Versicherungsumfang, Versicherungsdauer)	Es erfolgt keine studienspezifische Versicherung. Die Patienten sind in der Regelversorgung und die behandelnden Ärzte durch die Klinikhaftpflicht-Versicherung versichert.
25. Ggf.: Dokumentationsverfahren (Verweis auf CRF-Bögen möglich)	CRF werden mit den klinikinternen Dokumentationen, ergänzt durch VAS und NAS erstellt.
26. Ggf.: Beschreibung, wie der Gesundheitszustand gesunder betroffener Personen dokumentiert werden soll	----
27. Ggf.: Methoden, unerwünschte Ereignisse festzustellen, zu dokumentieren und mitzuteilen (wann, von wem und wie ??)	Initiale Meldung erfolgt von Studienteilnehmern an Studienärzte oder Studienleiter. Diese werden schriftlich innerhalb von 24 Stunden an den Studienleiter gemeldet und schriftlich dokumentiert.

28. Vorgehen zum Schutz der Geheimhaltung der gespeicherten Daten, Dokumente und ggf. Proben, Darlegung der Verschlüsselung der Daten von Studienteilnehmern (*bitte nicht Initialen und Geburtsdatum als Codierungsschema verwenden!)*	Im Rahmen der vorliegenden Studie werden alle Patientendaten in den Prüfbögen und der Datenbank nur über eine Patientennummer identifiziert.
29. Erklärung zur Einhaltung des Datenschutzes	Nach dem Bundesdatenschutzgesetz (BDSG) § 4(1)
30. Namen und Anschriften der Einrichtungen, die als Studienzentrum oder Studienlabor in die Studie eingebunden sind, sowie der Studienleiter und die Studienärzte	Studienleiter:<br>Prof. Dr. med. Andreas Michalsen<br>Stiftungsprofessur und Hochschulambulanz für Naturheilkunde der Charité - Universitätsmedizin Berlin. C/o Immanuel Krankenhaus Berlin<br>Abteilung für Naturheilkunde<br>Königstraße 63; 14109 Berlin<br>Tel.: 030/80505 690/-691<br>E-Mail: a.michalsen@immanuel.de<br><br>Studienzentrum Berlin:<br>Stiftungsprofessur und Hochschulambulanz für Naturheilkunde der Charité - Universitätsmedizin Berlin, c/o Immanuel Krankenhaus Berlin<br>Studienärzte: Dr. Christian Kessler, Larissa Meier<br>Study Nurse: Miriam Rösner<br><br>Studienzentrum Überlingen:<br>Klinik Buchinger Wilhelmi<br>Wilhelm- Beck- Straße 27<br>D- 88662 Überlingen<br>Studienärzte:Dr. Francoise Wilhelmi de Toledo, Dr. Stefan Drinda<br>Study Nurse: Franziska Grundler
31. Angaben zur Eignung der Prüfstelle, insbesondere zur Angemessenheit der dort vorhandenen Mittel und Einrichtungen sowie des zur Durchführung der klinischen Prüfung zur Verfügung stehenden Personals und zu Erfahrungen in der Durchführung ähnlicher Studien	Die Einrichtung des Studienleiters verfügt über eine mehrjährige Erfahrung in der Durchführung klinischer Studien und hat bereits zahlreiche klinische Studien durchgeführt. Diese Studien wurden innerhalb des vorgegebenen Zeitplanes erfolgreich durchgeführt. An der Einrichtung stehen in Studien erfahrenes Personal, Prüfärzte mit KKS/GCP Zertifikat sowie angemessene Räumlichkeiten und durchgehend nutzbare, moderne PC- Arbeitsplätze zur Verfügung.<br><br>An der Klinik Buchinger Wilhelmi stehen zwei Prüfarzte, eine Study Nurse mit KKS- Zertifikat sowie ein Ärzteteam mit zwei weiteren studienerfahrenen Ärzten zur Verfügung. Außerdem stehen angemessene Räumlichkeiten und durchgehend nutzbare moderne PC- Arbeitsplätze zur Verfügung.
32. Vereinbarung über den Zugang des Prüfers/Hauptprüfers/Leiter der klinischen Prüfung, zu den Daten und den Grundsätzen über die Publikation	Die Daten sind ausschließlich über den Studienleiter zugänglich.<br>Die Publikation erfolgt unabhängig vom Ergebnis in einer anerkannten Zeitschrift.
33. Angaben zur Finanzierung der Studie	Die Studienaufwendungen werden über hauseigene

(wir weisen auf § 263 StGB hin)	Mittel der beiden klinischen Einrichtungen finanziert.
a. Finanzierungsquelle (Name und Sitz)	Klinik Buchinger Wilhelmi; Überlingen<br>Immanuel Krankenhaus Berlin
b. Höhe der kalkulierten Kosten pro Teilnehmer und insgesamt	Die Kosten der Studie sind unterteilt in die medizinisch-inhaltliche Entwicklungsphase und die eigentliche Studiendurchführungsphase. Für die gesamte Studiendurchführung und wissenschaftliche Aufarbeitung werden Kosten von insgesamt 50.000 € angesetzt
c. Höhe der Kostenerstattung pro Teilnehmer und insgesamt	--------

Name und Unterschrift des/der Antragstellers:

Ich versichere hiermit, dass die in diesem Antrag gegebenen Informationen richtig sind. Ich bin der Auffassung, dass es möglich ist, die o.g. Studie in Übereinstimmung mit dem Protokoll, den nationalen Rechtsvorschriften durchzuführen.

Mir ist bekannt, dass ich gemäß §19 Berliner Datenschutzgesetz (BlnDSG) verpflichtet bin, für automatisierte Verarbeitungen personenbezogener und personenbeziehbarer Daten eine Datei- und Verfahrensbeschreibung zu erstellen und diese gemäß §19a dem behördlichen Datenschutzbeauftragten der Charité zur Verfügung stellen muss. Ich bin darüber informiert, dass wenn es sich um ein Verfahren handelt, mit dem Daten verarbeitet werden, die einem Berufsgeheimnis (z.B. ärztliche Schweigepflicht) unterliegen, ich gemäß §5 BlnDSG vor dem Einsatz dieses Verfahrens eine Vorabkontrolle durch den behördlichen Datenschutzbeauftragten der Charité veranlassen muss und ich das Verfahren erst bei positivem Prüfergebnis anwenden darf.

Name: Prof. Dr. med. Michalsen

Vorname: Andreas

Adresse: Königstrasse 63; 14109 Berlin

Position: Chefarzt, Professor für klinische Naturheilkunde der Charité – Universitätsmedizin Berlin

Datum: 22.02. 2015

Unterschrift:

Prof. Andreas Michalsen

Literatur

1. Fahrner H. Fasten als Therapie. Stuttgart: Hippokrates; 1991.
2. Fond G, Macgregor A, Leboyer M, Michalsen A. Fasting in mood disorders: neurobiology and effectiveness. A review of the literature. Psychiatry research. 2013;209:253-8.
3. Fontana L, Partridge L, Longo VD. Extending healthy life span--from yeast to humans. Science. 2010;328:321-6.
4. Gems D, Partridge L. Stress-response hormesis and aging: "that which does not kill us makes us stronger". Cell metabolism. 2008;7:200-3.
5. Gohler L, Hahnemann T, Michael N, Oehme P, Steglich HD, Conradi E, Grune T, Siems WG. Reduction of plasma catecholamines in humans during clinically controlled severe underfeeding. Preventive medicine. 2000;30:95-102
6. Goldhamer AC, Lisle DJ, Sultana P, Anderson SV, Parpia B, Hughes B, Campbell TC. Medically supervised water-only fasting in the treatment of borderline hypertension. J Altern Complement Med. 2002;8:643-50.
7. Horne BD, May HT, Anderson JL, Kfoury AG, Bailey BM, McClure BS, Renlund DG, Lappe DL, Carlquist JF, Fisher PW, Pearson RR, Bair TL, Adams TD, Muhlestein JB. Usefulness of routine periodic fasting to lower risk of coronary artery disease in patients undergoing coronary angiography. The American journal of cardiology. 2008;102:814-9
8. Kjeldsen-Kragh J, Haugen M, Borchgrevink CF, Laerum E, Eek M, Mowinkel P, Hovi K, Forre O. Controlled trial of fasting and one-year vegetarian diet in rheumatoid arthritis. Lancet. 1991;338:899-902.
9. Kouda K, Iki M. Beneficial effects of mild stress (hormetic effects): dietary restriction and health. Journal of physiological anthropology. 2010;29:127-32.
10. Lee C, Safdie FM, Raffaghello L, Wei M, Madia F, Parrella E, Hwang D, Cohen P, Bianchi G, Longo VD. Reduced levels of IGF-I mediate differential protection of normal and cancer cells in response to fasting and improve chemotherapeutic index. Cancer research. 2010;70:1564-72.
11. Li C, Ostermann T, Hardt M, Ludtke R, Broecker-Preuss M, Dobos G, Michalsen A. Metabolic and psychological response to 7-day fasting in obese patients with and without metabolic syndrome. Forsch Komplementmed. 2013;20:413-20.
12. Longo VD, Mattson MP. Fasting: molecular mechanisms and clinical applications. Cell metabolism. 2014;19:181-92.
13. Maoz E, Shamiss A, Peleg E, Salzberg M, Rosenthal T. The role of atrial natriuretic peptide in natriuresis of fasting. Journal of hypertension. 1992;10:1041-4.
14. Mattson MP, Wan R. Beneficial effects of intermittent fasting and caloric restriction on the cardiovascular and cerebrovascular systems. The Journal of nutritional biochemistry. 2005;16:129-37.
15. Mattson MP. Energy intake, meal frequency, and health: a neurobiological perspective. Annual review of nutrition. 2005;25:237-60.

16. Michalsen A, Schneider S, Rodenbeck A, Ludtke R, Huether G, Dobos GJ. The short-term effects of fasting on the neuroendocrine system in patients with chronic pain syndromes. Nutritional neuroscience. 2003;6:11-8.
17. Michalsen A, Li C. Fasting therapy for treating and preventing disease - current state of evidence. Forsch Komplementmed. 2013;20:444-53.
18. Michalsen A, Hoffmann B, Moebus S, Backer M, Langhorst J, Dobos GJ. Incorporation of fasting therapy in an integrative medicine ward: evaluation of outcome, safety, and effects on lifestyle adherence in a large prospective cohort study. J Altern Complement Med. 2005;11:601-7.
19. Michalsen A. Prolonged fasting as a method of mood enhancement in chronic pain syndromes: a review of clinical evidence and mechanisms. Current pain and headache reports. 2010;
14:80-7.
20. Müller H, Wilhelmi de Toledo F, Schuck P, Resch KL. Blutdrucksenkung durch Fasten bei adipösen und nichtadipösen Hypertonikern. Perfusion. 2001;14:108-12.
21. Muller H, de Toledo FW, Resch KL. Fasting followed by vegetarian diet in patients with rheumatoid arthritis: a systematic review. Scandinavian journal of rheumatology. 2001;30:1-10.
22. Stange R, Pflugbeil C, Michalsen A, Uehleke B. Therapeutic fasting in patients with metabolic syndrome and impaired insulin resistance. Forsch Komplementmed. 2013;20:421-6.
23. Varady KA, Hellerstein MK. Alternate-day fasting and chronic disease prevention: a review of human and animal trials. The American journal of clinical nutrition. 2007;
86:7-13.
24. Wan R, Ahmet I, Brown M, Cheng A, Kamimura N, Talan M, Mattson MP.
Cardioprotective effect of intermittent fasting is associated with an elevation of adiponectin levels in rats. The Journal of nutritional biochemistry. 2010;21:413-7.
25. Wilhelmi de Toledo F, Buchinger A, Burggrabe H, et al.: Fasting therapy – an Expert Panel Update of the 2002 Consensus Guidelines. Forsch Komplementmed 2013;20:444–453
