# Supplementary material for: Safety, health improvement and well-being during a 4 to 21-day fasting period in an observational study including 1422 subjects
Source: PLoS One. 2019 Jan 2;14(1):e0209353. doi: 10.1371/journal.pone.0209353 (PMC6314618; doi:10.1371/journal.pone.0209353)
Supplement: S3 Text — (PDF) [file pone.0209353.s020.pdf]

S3 Text. Study protocol translated to English

Application for consultation by the Ethics Committee for the implementation of a medical-scientific project which does not include the clinical trial of a medicinal product

1. Title of the study	Scientific documentation of the Buchinger Wilhelmi program (modified fasting, caloric restriction and vegetarian diet)
2. Ethics Commission application number	
3. Decisions of other ethics committees in the same case	-----
4. Subject of the study and its objectives; specification of the hypotheses separated into main and secondary hypotheses and the clinical parameters (primary and secondary endpoints) against which the hypotheses are assessed	Aim of the study is the prospective scientific documentation of the clinical effects of an inpatient / clinical therapy with focus on modified fasting and caloric restriction by pre- / post- and group comparisons as well as a selective written follow-up in consecutive regular patients (Outcome research)<br><br><u>Exploratory target parameters:</u><br><br>At the beginning, during and at the end of the inpatient stay as well as up to 12 months after the stay via emailing, the clinic-specific surveys of the target parameters and the analysis of changes of the following study endpoints are carried out:<br><br>- Height<br>- Blood pressure, systolic / diastolic in rest<br>- Clinical laboratory examination<br>- Weight<br>- Abdominal circumference<br>- Heart frequency in rest<br>- Mood (Profile of Mood States), well-being (visual analogue scale), stress (Cohen range), quality of life (WHO-5)<br>- Subjective stress experience<br>- Quantification (subjective self-report) of behavioral factors: nicotine, alcohol, amount of exercise, coffee, media consumption<br>- Subjective intensity of the main health complaint<br>- Mild symptoms and adverse events<br><br>In addition, there is a systematic documentation of the medication, the main

	and secondary diagnoses and the nutritional therapy.<br><br>Following the stay, the behavioral factors and the subjectively perceived effects of fasting or calorie restriction are asked by means of emailing in order to record the sustainability.
5. Explanation of the meaning of the study	Therapeutic fasting has been successfully used in natural medicine and integrative medicine for many decades in the treatment of chronic internal diseases and pain syndromes (1,11,17,18,21). Especially in chronic rheumatic, inflammatory and metabolic disorders, fasting therapy is used in specialized clinical settings (fasting clinics) with increasing patient request. Furthermore, in the field of classical naturopathy and naturopathic complex treatment fasting is an integral part of the therapy core modules.<br><br>Within the various historically increased patterns of fasting, the fasting program according to the Buchinger Wilhelmi method has established itself worldwide as the most frequently used method (25). Thereby a subtotal caloric restriction with a daily calorie intake (200-400kcal / day) as liquid components over a defined period of at least 10 days is performed and accompanied by supporting measures of a healthy lifestyle program with elements such as exercise, physiotherapy, stress reduction and hydro-balneotherapy (25).<br><br>In early randomized studies and in a systematic review, the efficacy of inpatient treatment for the indication of rheumatoid arthritis was confirmed to be 1α (8,21). For the further indications, there are predominantly empirical evidence or data from observational or prospective uncontrolled studies (5,6,16-19,22). In recent years, extensive fundamental research activity has developed in the field of calorie restriction and intermittent fasting. Here, a large number of beneficial findings from animal experiments could be substantiated by defined fasting periods, i.a. decreases in insulin, IGF-1, increases in adiponectin, insulin sensitivity, neurotrophic factors, and over a longer period of observation a decrease in the incidence of cardiovascular, inflammatory, metabolic and, more recently, oncological diseases in a wide variety of animal species (3,4,9,10,12,15,23,24).<br><br>In this context, it is scientifically meaningful to document systematically the clinical effects of fasting in a larger treated human population,

	in particular to gain further methodological information for future clinical studies and to analyze subpopulations and responders. The proposed study is intended to systematically document and analyze a larger population (n> 1000) prospectively for the first time.
6. Which of the following rules apply?<br>• Law concerning medical devices - according § 20 medical devices act (MPG) (The device does not have the declaration of conformity<br>it is used and another indication is being tested<br>additional invasive or other stressful examinations are being carried out) **or** - according § 23 MPG ?<br>• Radiation Protection Regulation § 23<br>• X-ray Regulation § 28 a<br>• Genetic Engineering Law<br>• Data protection laws	Data protection law
7. if necessary: name and characterization of the products tested (e.g. Devices in MPG studies; please attach enclosures)	----
8. significant results of preclinical tests or reasons for not performing them	It exists in the field of the physiological effects of calorie restriction and intermittent fasting comprehensive data from animal experiments [3,12,14], indicating the preventive and / or therapeutic health-promoting effects for a variety of degenerative, inflammatory, cardiovascular and oncological diseases.
9. Key content and results of previous studies / applications of the products to be tested in the study	Periodic fasting and intermittent fasting have shown beneficial effects on cardio metabolic, gluco-regulatory, and neuroendocrine-immunological function in a large number of experimental and clinical studies. They show significant reduction of insulin, leptin, IGF-1 and others [3,12,14,15] and elevation of neurotrophic factors, adiponectin, natriuretic peptides as mediators of potential beneficial effects being actually discussed and investigated [13,17,24].<br>The randomized studies showed clinically relevant effects on the course of rheumatoid arthritis [8,21]. Controlled non-randomized and uncontrolled intervention studies found beneficial effects on blood pressure, insulin sensitivity, chronic musculoskeletal pain, mental health and a recent RCT (in publication) on the quality of life in multiple sclerosis [2,6, 11,17,16,22]. Previous studies with prolonged observational time, have found improved compliance with healthy lifestyle choices and healthy diets, as well as no serious adverse effects of fasting [18].
10. Description of intended measures / methods of investigation and possible deviations from those in the med. practice usual measures / investigations (what is	Regular consecutive in-patient and clinical patients of the Buchinger Wilhelmi Clinic in Überlingen and the Department of Naturopathy at the Immanuel Hospital Berlin are requested to participate in the study.

"routine", what is different in the study?)	The duration of treatment will usually be between 10 days to 4 weeks.<br>Hereby, no influence on the therapeutic interventions will occur within the framework of the study. The patients receive a nutritional therapy according to their preference and / or indication, a therapeutic fasting according to the Buchinger method or a specified calorically reduced diet (800 / 1200kcal) or in case of existing contra-indications. A normocaloric diet is prescribed in isolated cases. An accompanying lifestyle and naturopathy program is part of the program independently to the nutritional strategy.<br><br>The subjects are asked if they want to participate in the study during the medical admission interview with the doctor.<br>The scientific documentation includes the diagnostic measures and findings obtained as part of the usual clinical procedure. It includes also daily measurements of blood pressure, heart frequency, weight, abdominal circumference and the standard clinical laboratory examinations, at the beginning and at the end of the stay. Furthermore, following data is collected as part of the regular clinical procedure: the documentation and assessments of the self-assessed physical and psychological overall condition, the lifestyle and dietary habits before and during the stay, the medication, the general assessment of the evolution of the main health complaint(s) and the overall satisfaction. Possible adverse effects associated with fasting or other nutritional therapy are documented daily.<br>There are no further measures that deviate from the routine.
11. Assessment and consideration of the foreseeable risks and disadvantages of study participation compared to the expected benefits for the study participants and to persons who might have indications for the intervention in future (benefit-risk-assessment)	Study participants can benefit individually while serious risks to the process being evaluated have not been observed until now. In summary, there is a positive benefit-risk assessment.
a. Predictable therapeutic benefit for the study participants (individual benefit for the individual patient)	All participants receive a regular potentially effective therapy (hospital stay, therapeutic fasting or calorie reduced diet). The present study provides a systematic, accurate clinical documentation. This raises a predictable potential benefit through the participation in the study.
b. Foreseeable medical benefit to treat illnesses **(group benefit)**	The results of this study may help to improve the medical care of patients through fasting therapy. This is a general benefit for future patients given by the study results.
c. **Risks** and burdens for the study participants (list each one separately)	All surveys do not go beyond the usual clinical routine except for a few self-assessment questions. All in all, there is no relevant health risk from the study participation.
12. Measures for risk governance	Participants are urged to promptly report any possible

	adverse events (AEs). AEs are evaluated by the study leader and investigators and the study progress is monitored accordingly.
13. Break-off criteria	Each AE is assessed by the investigator for its severity and possible association with the investigated therapy. Severe AEs and AEs must be reported to the Clinical Trial Director by telephone, telefax or telegram within 24 hours by the investigator.<br>If the patient withdraws his consent, the investigator must end the study. This will be noted in the case report form.<br>The study may be interrupted ahead of time if it becomes apparent that the study cannot fulfill the mentioned requirements. These include:<br>- occurrence of serious protocol violations<br>- the CRFs are filled in inadequately or intentionally incorrectly<br>- legal or ethical regulations are not respected<br>- the study can only be interrupted for the above reasons if agreement between the study leader and the investigators.<br><br>If severe AEs or relevant AEs occur or if non-serious AEs accumulate, the study director may, decide on his own, to stop the study. The study must be interrupted if the safety of the therapy is questioned due to adverse events.
14. Number, age and sex of the persons concerned	The studies will be conducted in two centers that will treat mixed patient populations<br>1. Buchinger Wilhelmi Clinic in Überlingen<br>2. Immanuel Hospital Berlin, Dept. of Integrative Medicine and Naturopathy / Institute of Social Medicine, Epidemiology and Helath Economics at the University Hospital Charité Berlin<br>These centers treat in-patients (n = 2000 and n = 1000 patients with fasting therapies) in most cases with a slightly higher proportion of women compared to men, with fasting patients of all ages with a focus between 45-65 years and a spectrum of 18-99 years.
15. Statistical planning and indication as well as biometric justification of the number of cases and signature of the statistician	The statistical analysis compares exploratively the pre-post data of all clinical parameters and inventories to estimate fasting in intragroup comparison. There is also a group comparison of fasting patients versus patients receiving moderate calorie restriction. The group comparisons are carried out exploratively with ANCOVA.<br>Finally, all patients receive a follow-up by email 7 days, 3 and 12 months after discharge. The statistical analysis is carried out at the Institute of Social Medicine, Epidemiology and Helath Economics at the University Hospital Charité Berlin.<br><br>Number of cases: a case number calculation is due to the nature of an outcome research with pre- to post-comparisons not necessary. The goal is the inclusion

	of a total of at least n> 1000 patients over a period of 2 years.
16.<br>a. Presentation and, if necessary, explanation of the **inclusion and exclusion criteria**	Inclusion criteria:<br>- Age 18-99 years<br>- Beginning (first 24h) inpatient treatment or hospital stay at one of the two centers in Überlingen or Berlin<br>- Present written consent<br><br>Exclusion criteria:<br>- insufficient linguistic understanding<br>- Dementia or other severely debilitating cognitive disease<br>- Existing pregnancy or lactation period<br>- Participation in another study
b. **Participant information** (who gives this orally? Information about how much time goes on between first information and consent. Reference to the patient information given as an attachment possible)	- Clinicians give personally the initial information and a written patient information is handed out. There will not be more than 24 hours between the first information and consent.
c. **Declaration of consent** (reference to its content as attachment possible)	See attachment
d. if necessary, **information and consent of the legal representative** (if necessary also description of the procedure for establishing a judicial assistance)	---
17. Measures for the acquisition of study participants (notice, newspaper advertisements, etc.)	At the two centers in Überlingen and Berlin, the patients are informed about the possibility of participating in the study during the inpatient admission interview.
18. If necessary: **Reason for the inclusion and presentation of the therapeutic benefit for persons who are minors and / or not able to consent.**	--
19. Relationship between study participant and study doctor (Is the study doctor at the same time the attending physician?)	In individual cases this is possible at the clinic Buchinger Wilhelmi.
20. Explanation regarding the involvement of any person dependent on the sponsor or study doctor	--
21. Actions that allow a determination of whether a study participant participates in several studies at the same time or before the expiry of a period set in the previous study.	All patients are interviewed explicitly after participating in the study and advised that simultaneous participation in other clinical trials during the study period is not possible.
22. If necessary: remuneration or reimbursement of study participants (Amount, what should be paid for?)	---
23. If necessary: Plan for the follow up and medical care of the affected persons after the end of the study	---
24. If necessary: Insurance of study participants (Confirmation of insurance and conditions of insurance, insurer, extend of insurance, period of insurance)	There is no study-specific insurance. Patients are insured under standard care and the treating physicians are insured by the clinical liability insurance.
25. If necessary: Documentation procedure (reference to CRF sheets possible)	CRF are created with in-clinic documentation, supplemented by visual analogue scales and numeric analogue scales.

26. If necessary: Description of how the health status of healthy persons should be documented	----
27. If necessary: Methods to detect, document and communicate adverse events (when, by whom and how?)	Initial notification is made by study participants to study physicians or study leaders. These will be reported in writing within 24 hours to the study leader and documented in writing.
28. Procedure for protecting the confidentiality of the stored data, documents and, if necessary, samples, presentation of the encryption of the data of study participants (please do not use initials and date of birth as coding scheme!)	In the present study all patient data in the case report forms and database are only identified by a patient number.
29. Declaration of observance of the data protection	According to the Federal Data Protection Act (BDSG) § 4(1)
30. Names and addresses of institutions involved in the study as study center or study laboratory, as well as the study director and the study physicians	Head of study:<br>Prof. Andreas Michalsen<br>Institute of Social Medicine, Epidemiology and Helath Economics at the University Hospital Charité Berlin.<br>C/o Department of Naturopathy<br>Immanuel Hospital Berlin, Dept. of Integrative Medicine and Naturopathy<br>Königstraße 63; 14109 Berlin<br>Phone Nr.: 030/80505 690/-691<br>E-Mail: a.michalsen@immanuel.de<br><br>Study center Berlin:<br>Institute of Social Medicine, Epidemiology and Helath Economics at the University Hospital Charité Berlin.<br>C/o Department of Naturopathy<br>Immanuel Hospital Berlin, Dept. of Integrative Medicine and Naturopathy<br>Investigators: Dr. Christian Kessler, Larissa Meier<br>Study Nurse: Miriam Rösner<br><br>Study center Überlingen:<br>Buchinger Wilhelmi Clinic<br>Wilhelm- Beck- Straße 27<br>D- 88662 Überlingen<br>Investigators: Dr. Françoise Wilhelmi de Toledo, Dr. Stefan Drinda<br>Study Nurse: Franziska Grundler
31. Information about the suitability of the testing center, in particular on the adequacy of the resources and facilities available there, as well as the staff available to carry out the clinical trial and on experience in carrying out similar studies	The lead investigator's facility has many years of experience in conducting clinical trials and has completed numerous clinical trials. These studies were successfully completed within the given timetable. Experienced staff, investigators with KKS / GCP certification as well as adequate premises and modern PC workstations, which can be used continuously, are available at the facility.<br><br>At Buchinger Wilhelmi Clinic, there are two

	investigators, a study nurse with a KKS certificate and a medical team with two other doctors experienced in the field. In addition, adequate space and continuously usable modern PC workstations are available.
32. Agreement on the access of the examiner / principal examiner / director of the clinical trial, the data and the principles of the publication	The data are only accessible via the study director. The publication is independent of the result in a recognized journal.
33. Information on the financing of the study (we refer to § 263 StGB)	Study costs will be financed by the both clinical facilities.
a. Funding source (name and headquarter)	Buchinger Wilhelmi Clinic, Überlingen<br>Immanuel Hospital Berlin
b. Amount of the calculated costs per participant and total	The costs of the study are divided into the medical content-related development phase and the actual study execution phase. The costs of the entire study and scientific work total € 50,000
c. Amount of reimbursement per participant and total	--------

Name and signature of the applicant:

I hereby confirm that the information given in this application is correct. I believe that it is possible to use the above mentioned study in accordance with the Protocol to implement national legislation.

I am aware that in accordance with §19 Berlin Data Protection Act (BlnDSG) I am obliged to prepare a file and process description for automated processing of personal and personal data and make it available to the Charité Data Protection Officer in accordance with §19a. I am informed that if this is a procedure for the processing of data subject to professional secrecy (eg medical confidentiality), I must arrange, in accordance with §5 BlnDSG, prior checking by the official data protection officer of the Charité and I can use the procedure only if the result of the test is positive.

Name: Prof. Michalsen

Surname: Andreas

Address: Königstrasse 63; 14109 Berlin

Position: chief doctor, Professor of Clinical Naturopathy of the Charité – University Berlin

Date: 22.02.2015

Signature:

Prof. Andreas Michalsen

Literature

1. Fahrner H. Fasten als Therapie. Stuttgart: Hippokrates; 1991.
2. Fond G, Macgregor A, Leboyer M, Michalsen A. Fasting in mood disorders: neurobiology and effectiveness. A review of the literature. Psychiatry research. 2013;209:253-8.
3. Fontana L, Partridge L, Longo VD. Extending healthy life span--from yeast to humans. Science. 2010;328:321-6.
4. Gems D, Partridge L. Stress-response hormesis and aging: "that which does not kill us makes us stronger". Cell metabolism. 2008;7:200-3.
5. Gohler L, Hahnemann T, Michael N, Oehme P, Steglich HD, Conradi E, Grune T, Siems WG. Reduction of plasma catecholamines in humans during clinically controlled severe underfeeding. Preventive medicine. 2000;30:95-102
6. Goldhamer AC, Lisle DJ, Sultana P, Anderson SV, Parpia B, Hughes B, Campbell TC. Medically supervised water-only fasting in the treatment of borderline hypertension. J Altern Complement Med. 2002;8:643-50.
7. Horne BD, May HT, Anderson JL, Kfoury AG, Bailey BM, McClure BS, Renlund DG, Lappe DL, Carlquist JF, Fisher PW, Pearson RR, Bair TL, Adams TD, Muhlestein JB. Usefulness of routine periodic fasting to lower risk of coronary artery disease in patients undergoing coronary angiography. The American journal of cardiology. 2008;102:814-9
8. Kjeldsen-Kragh J, Haugen M, Borchgrevink CF, Laerum E, Eek M, Mowinkel P, Hovi K, Forre O. Controlled trial of fasting and one-year vegetarian diet in rheumatoid arthritis. Lancet. 1991;338:899-902.
9. Kouda K, Iki M. Beneficial effects of mild stress (hormetic effects): dietary restriction and health. Journal of physiological anthropology. 2010;29:127-32.
10. Lee C, Safdie FM, Raffaghello L, Wei M, Madia F, Parrella E, Hwang D, Cohen P, Bianchi G, Longo VD. Reduced levels of IGF-I mediate differential protection of normal and cancer cells in response to fasting and improve chemotherapeutic index. Cancer research. 2010;70:1564-72.
11. Li C, Ostermann T, Hardt M, Ludtke R, Broecker-Preuss M, Dobos G, Michalsen A. Metabolic and psychological response to 7-day fasting in obese patients with and without metabolic syndrome. Forsch Komplementmed. 2013;20:413-20.
12. Longo VD, Mattson MP. Fasting: molecular mechanisms and clinical applications. Cell metabolism. 2014;19:181-92.
13. Maoz E, Shamiss A, Peleg E, Salzberg M, Rosenthal T. The role of atrial natriuretic peptide in natriuresis of fasting. Journal of hypertension. 1992;10:1041-4.
14. Mattson MP, Wan R. Beneficial effects of intermittent fasting and caloric restriction on the cardiovascular and cerebrovascular systems. The Journal of nutritional biochemistry. 2005;16:129-37.
15. Mattson MP. Energy intake, meal frequency, and health: a neurobiological perspective. Annual review of nutrition. 2005;25:237-60.
16. Michalsen A, Schneider S, Rodenbeck A, Ludtke R, Huether G, Dobos GJ. The short-term effects of fasting on the neuroendocrine system in patients with chronic pain syndromes. Nutritional neuroscience. 2003;6:11-8.

17. Michalsen A, Li C. Fasting therapy for treating and preventing disease - current state of evidence. Forsch Komplementmed. 2013;20:444-53.

18. Michalsen A, Hoffmann B, Moebus S, Backer M, Langhorst J, Dobos GJ. Incorporation of fasting therapy in an integrative medicine ward: evaluation of outcome, safety, and effects on lifestyle adherence in a large prospective cohort study. J Altern Complement Med. 2005;11:601-7.

19. Michalsen A. Prolonged fasting as a method of mood enhancement in chronic pain syndromes: a review of clinical evidence and mechanisms. Current pain and headache reports. 2010;
14:80-7.

20. Müller H, Wilhelmi de Toledo F, Schuck P, Resch KL. Blutdrucksenkung durch Fasten bei adipösen und nichtadipösen Hypertonikern. Perfusion. 2001;14:108-12.

21. Muller H, de Toledo FW, Resch KL. Fasting followed by vegetarian diet in patients with rheumatoid arthritis: a systematic review. Scandinavian journal of rheumatology. 2001;30:1-10.

22. Stange R, Pflugbeil C, Michalsen A, Uehleke B. Therapeutic fasting in patients with metabolic syndrome and impaired insulin resistance. Forsch Komplementmed. 2013;20:421-6.

23. Varady KA, Hellerstein MK. Alternate-day fasting and chronic disease prevention: a review of human and animal trials. The American journal of clinical nutrition. 2007;
86:7-13.

24. Wan R, Ahmet I, Brown M, Cheng A, Kamimura N, Talan M, Mattson MP.
Cardioprotective effect of intermittent fasting is associated with an elevation of adiponectin levels in rats. The Journal of nutritional biochemistry. 2010;21:413-7.

25. Wilhelmi de Toledo F, Buchinger A, Burggrabe H, et al.: Fasting therapy – an Expert Panel Update of the 2002 Consensus Guidelines. Forsch Komplementmed 2013;20:444–453
