# Supplementary material for: Safety, health improvement and well-being during a 4 to 21-day fasting period in an observational study including 1422 subjects
Source: PLoS One. 2019 Jan 2;14(1):e0209353. doi: 10.1371/journal.pone.0209353 (PMC6314618; doi:10.1371/journal.pone.0209353)
Supplement: S4 Text — (PDF) [file pone.0209353.s021.pdf]

S4 Text. Questionnaires in German.

Tagebuch

zur Selbstbeobachtung

Name: ________________________________

Zimmernummer: ________________________

Aufenthalt von: __________ bis: ______________

Liebe Patientinnen und Patienten,
liebe Gäste,

die Umstellung des Stoffwechsels durch das Fasten oder eine kalorienreduzierte vegetarische Diät kann sich auf das persönliche Befinden auswirken. Mit Hilfe dieses Tagebuchs können die Wirkungen erfasst und besser verstanden werden. Außerdem kann es dazu dienen, die Therapieempfehlungen in Zukunft zu optimieren.

Nur eine lückenlose Dokumentation erlaubt es Zusammenhänge zu erkennen. Daher bitten wir Sie, das Tagebuch täglich, möglichst abends, detailgetreu auszufüllen.

Zudem bitten wir Sie, folgende Angaben zu machen:

1. Lebensstil – vor dem Aufenthalt (S. 7)
2. Lebensstil – während des Aufenthalts (S. 53)
3. Fragen am Ende des Aufenthalts (S. 52)

Geben Sie bitte am Abreisetag das vollständig ausgefüllte Tagebuch an Ihre Stationsschwester zurück.

Vielen Dank für Ihre Teilnahme!

Dr. Françoise Wilhelmi de Toledo und
das wissenschaftliche Team der Klinik Buchinger Wilhelmi

Anleitung:

- Datum:
  Bitte tragen Sie als erstes das Datum ein. Der erste Tag entspricht dem Anreisetag.

- Kostform:
  Die zutreffende Kostform für den aktuellen Tag wird mit einem Kreuz markiert und die zugehörigen Angaben gegebenenfalls angekreuzt. Bitte markieren Sie nicht zutreffende Aussagen mit einem Kreuz im Nein-Feld.

- Ketonkörper:
  Messen Sie die Ketonkörper mit Hilfe der Ketostix im ersten Morgenurin und geben Sie das Resultat im vorgesehenen Feld an. Bitte beachten Sie, dass nur ein Wert angegeben werden kann.

- Körperliches und psychisch-seelisches Befinden:
  Beurteilen Sie intuitiv Ihr körperliches und emotionales Befinden auf einer Skala zwischen 0 und 10. Bitte legen Sie sich auf einen Wert fest.

- Beschwerden:
  Falls keine Beschwerden aufgetreten sind, kreuzen Sie bitte Nein an. Falls doch, markieren Sie Ja und wählen ein bzw. mehrere Beschwerden, die aufgetreten sind.

- Wohlbefinden am heutigen Tag:
  Schätzen Sie, wie häufig jede der aufgeführten Aussagen am heutigen Tag auftrat und markieren Sie eine Zahl zwischen 0 und 5.

Beispieltag: Montag, 18.01.16

Ketonkörper (mg/dl): ☐ 0 ☐ 5 ☐ 15 ☒ 40 ☐ 80 ☐ 160

○ **Diät:** ☐ 800 kcal ☐ 1.200 kcal ☐ 1.800 kcal

Zusätze: ☐ nein ☐ Fisch
☐ andere: ______________________

○ **Entlastungstag:** ☐ Reis ☐ Kartoffel/Gemüse
☐ Früchte ☐ andere: __________

☒ **Fasten:** Honig ☐ nein ☒ ja

	Honig	☐ nein	☒ ja
mittags:	Saft (¼ l)	☐ nein	☒ ja
	Gemüsebrühe (¼ l)	☒ nein	☐ ja
abends:	Gemüsebrühe (¼ l)	☐ nein	☒ ja

Zusätze: ☐ nein ☐ Kousmine ☐ Kousmine vegan
☒ Joghurt ☐ Haferschleim
☐ Reisschleim ☐ Karottensaft
☐ grüner Tee ☐ schwarzer Tee
☐ andere: ______________________

○ **Fastenbrechen**

○ **Aufbautag:** ☐ 1 ☐ 2 ☐ 3 ☐ 4

BEISPIEL

Körperliches Befinden:

sehr schlecht | 0 | 1 | ~~2~~ (angekreuzt) | 3 | 4 | 5 | 6 | 7 | 8 | 9 | 10 | hervorragend

Psychisch-seelisches Befinden:

sehr schlecht | 0 | 1 | 2 | 3 | 4 | 5 | 6 | 7 | ~~8~~ (angekreuzt) | 9 | 10 | hervorragend

In der Klinik neu aufgetretene Beschwerden: ☒ nein ☐ ja

☐ Kopfschmerzen
☐ Schlafstörungen
☐ Mundgeruch
(fragen Sie die Schwester)
☐ Müdigkeit
☐ Rückenschmerzen
☐ Kälteempfindlichkeit
☐ Schwindel
☐ Hunger
☐ Hautausschlag
☐ Blähungen
☐ sonstige: ____________________

☐ Muskelschmerzen
☐ Sodbrennen
☐ Übelkeit
☐ Trockener Mund
☐ Wadenkrämpfe
☐ Durchfall
☐ Unruhige Beine
☐ Obstipation
☐ Herzklopfen
☐ Sehstörungen
☐ Essdrang

BEISPIEL

Am heutigen Tag…	zu keinem Zeitpunkt	ab und zu	etwas weniger als die Hälfte der Zeit	etwas mehr als die Hälfte der Zeit	meistens	die ganze Zeit
…war ich froh und guter Laune	0	1	2	3	☒ 4	5
…habe ich mich ruhig und entspannt gefühlt	0	1	☒ 2	3	4	5
…habe ich mich energisch und aktiv gefühlt	0	1	2	3	4	☒ 5
…habe ich mich beim Aufwachen frisch und ausgeruht gefühlt	☒ 0	1	2	3	4	5
…war mein Alltag voller Dinge, die mich interessieren	0	1	2	3	☒ 4	5

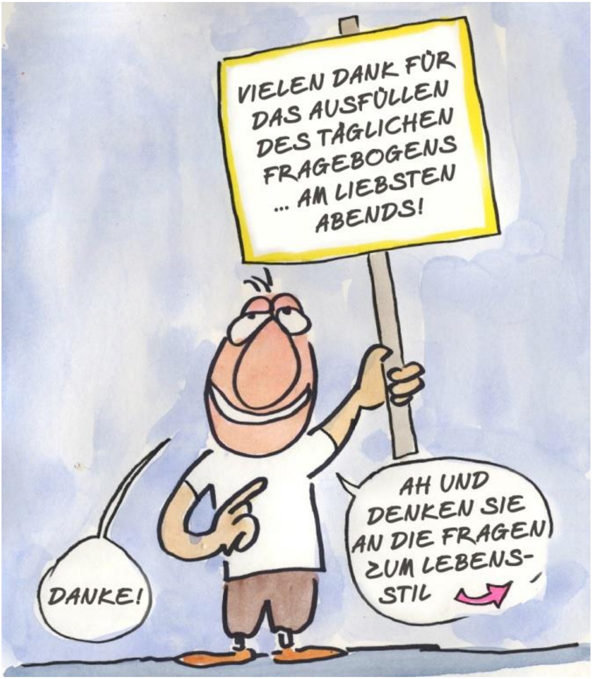
VIELEN DANK FÜR DAS AUSFÜLLEN DES TÄGLICHEN FRAGEBOGENS ... AM LIEBSTEN ABENDS!
DANKE!
AH UND DENKEN SIE AN DIE FRAGEN ZUM LEBENS-STIL

Lebensstil – vor dem Aufenthalt

Stress-/Entspannungsniveau:

bedrohliche Anspannung	0	1	2	3	4	5	6	7	8	9	10	total entspannt

**Nikotin:**	☐ nein	☐ Zigaretten/Tag:	☐☐
	☐ nein	☐ Zigarren/Tag:	☐☐
**Alkohol:**	☐ nein	☐ Bier: Gläser (0,5 l)/Woche:	☐☐
	☐ nein	☐ Wein: Gläser (0,25 l)/Woche:	☐☐
	☐ nein	☐ Schnaps: Gläser (2 cl)/Woche:	☐☐
**Kaffee:**	☐ nein	☐ Tassen/Tag:	☐☐
**Medien:**	☐ nein	☐ Smartphone (h/Woche):	☐☐
	☐ nein	☐ TV (h/Woche):	☐☐
	☐ nein	☐ PC (h/Woche):	☐☐
**Sport/ Bewegung:**	☐ nein	☐ ja: h/Woche:	☐☐ , ☐

**In den letzten zwei Wochen vor meinem Aufenthalt…**	zu keinem Zeitpunkt	ab und zu	etwas weniger als die Hälfte der Zeit	etwas mehr als die Hälfte der Zeit	meistens	die ganze Zeit
…war ich froh und guter Laune	0	1	2	3	4	5
…habe ich mich ruhig und entspannt gefühlt	0	1	2	3	4	5
…habe ich mich energisch und aktiv gefühlt	0	1	2	3	4	5
…habe ich mich beim Aufwachen frisch und ausgeruht gefühlt	0	1	2	3	4	5
…war mein Alltag voller Dinge, die mich interessieren	0	1	2	3	4	5

1. Tag (Anreise): ______________________

Ketonkörper (mg/dl): ☐ 0 ☐ 5 ☐ 15 ☐ 40 ☐ 80 ☐ 160

○ **Diät:** ☐ 800 kcal ☐ 1.200 kcal ☐ 1.800 kcal

Zusätze: ☐ nein ☐ Fisch
☐ andere: ______________________

○ **Entlastungstag**: ☐ Reis ☐ Kartoffel/Gemüse
☐ Früchte ☐ andere: __________

○ **Fasten:**

	Honig	☐ nein	☐ ja
mittags:	Saft (¼ l)	☐ nein	☐ ja
	Gemüsebrühe (¼ l)	☐ nein	☐ ja
abends:	Gemüsebrühe (¼ l)	☐ nein	☐ ja

Zusätze: ☐ nein ☐ Kousmine ☐ Kousmine vegan
☐ Joghurt ☐ Haferschleim
☐ Reisschleim ☐ Karottensaft
☐ grüner Tee ☐ schwarzer Tee
☐ andere: ______________________

○ **Fastenbrechen**

○ **Aufbautag:** ☐ 1 ☐ 2 ☐ 3 ☐ 4

Körperliches Befinden:

sehr schlecht	0	1	2	3	4	5	6	7	8	9	10	hervorragend

Psychisch-seelisches Befinden:

sehr schlecht	0	1	2	3	4	5	6	7	8	9	10	hervorragend

In der Klinik neu aufgetretene Beschwerden: ☐ nein ☐ ja

☐ Kopfschmerzen
☐ Schlafstörungen
☐ Mundgeruch
(fragen Sie die Schwester)
☐ Müdigkeit
☐ Rückenschmerzen
☐ Kälteempfindlichkeit
☐ Schwindel
☐ Hunger
☐ Hautausschlag
☐ Blähungen
☐ Muskelschmerzen
☐ Sodbrennen
☐ Übelkeit
☐ Trockener Mund
☐ Wadenkrämpfe
☐ Durchfall
☐ Unruhige Beine
☐ Obstipation
☐ Herzklopfen
☐ Sehstörungen
☐ Essdrang
☐ sonstige: ____________________

**Am heutigen Tag…**	zu keinem Zeitpunkt	ab und zu	etwas weniger als die Hälfte der Zeit	etwas mehr als die Hälfte der Zeit	meistens	die ganze Zeit
…war ich froh und guter Laune	0	1	2	3	4	5
…habe ich mich ruhig und entspannt gefühlt	0	1	2	3	4	5
…habe ich mich energisch und aktiv gefühlt	0	1	2	3	4	5
…habe ich mich beim Aufwachen frisch und ausgeruht gefühlt	0	1	2	3	4	5
…war mein Alltag voller Dinge, die mich interessieren	0	1	2	3	4	5

2. Tag: ______________________________

Ketonkörper (mg/dl): ☐ 0 ☐ 5 ☐ 15 ☐ 40 ☐ 80 ☐ 160

○ **Diät:** ☐ 800 kcal ☐ 1.200 kcal ☐ 1.800 kcal

Zusätze: ☐ nein ☐ Fisch
☐ andere: ______________________

○ **Entlastungstag**: ☐ Reis ☐ Kartoffel/Gemüse
☐ Früchte ☐ andere: __________

○ **Fasten:** Honig ☐ nein ☐ ja

mittags: Saft (¼ l) ☐ nein ☐ ja
Gemüsebrühe (¼ l) ☐ nein ☐ ja
abends: Gemüsebrühe (¼ l) ☐ nein ☐ ja

Zusätze: ☐ nein ☐ Kousmine ☐ Kousmine vegan
☐ Joghurt ☐ Haferschleim
☐ Reisschleim ☐ Karottensaft
☐ grüner Tee ☐ schwarzer Tee
☐ andere: ______________________

○ **Fastenbrechen**

○ **Aufbautag:** ☐ 1 ☐ 2 ☐ 3 ☐ 4

Körperliches Befinden:

sehr schlecht	0	1	2	3	4	5	6	7	8	9	10	hervorragend

Psychisch-seelisches Befinden:

sehr schlecht	0	1	2	3	4	5	6	7	8	9	10	hervorragend

In der Klinik neu aufgetretene Beschwerden: ☐ nein ☐ ja

- ☐ Kopfschmerzen
- ☐ Schlafstörungen
- ☐ Mundgeruch
  (fragen Sie die Schwester)
- ☐ Müdigkeit
- ☐ Rückenschmerzen
- ☐ Kälteempfindlichkeit
- ☐ Schwindel
- ☐ Hunger
- ☐ Hautausschlag
- ☐ Blähungen
- ☐ Muskelschmerzen
- ☐ Sodbrennen
- ☐ Übelkeit
- ☐ Trockener Mund
- ☐ Wadenkrämpfe
- ☐ Durchfall
- ☐ Unruhige Beine
- ☐ Obstipation
- ☐ Herzklopfen
- ☐ Sehstörungen
- ☐ Essdrang
- ☐ sonstige: ____________________

**Am heutigen Tag...**	zu keinem Zeitpunkt	ab und zu	etwas weniger als die Hälfte der Zeit	etwas mehr als die Hälfte der Zeit	meistens	die ganze Zeit
...war ich froh und guter Laune	0	1	2	3	4	5
...habe ich mich ruhig und entspannt gefühlt	0	1	2	3	4	5
...habe ich mich energisch und aktiv gefühlt	0	1	2	3	4	5
...habe ich mich beim Aufwachen frisch und ausgeruht gefühlt	0	1	2	3	4	5
...war mein Alltag voller Dinge, die mich interessieren	0	1	2	3	4	5

3. Tag: ______________________________

Ketonkörper (mg/dl): ☐ 0 ☐ 5 ☐ 15 ☐ 40 ☐ 80 ☐ 160

○ **Diät:** ☐ 800 kcal ☐ 1.200 kcal ☐ 1.800 kcal

Zusätze: ☐ nein ☐ Fisch
☐ andere: ____________________

○ **Entlastungstag**: ☐ Reis ☐ Kartoffel/Gemüse
☐ Früchte ☐ andere: ________

○ **Fasten:** Honig ☐ nein ☐ ja

mittags: Saft (¼ l) ☐ nein ☐ ja
Gemüsebrühe (¼ l) ☐ nein ☐ ja
abends: Gemüsebrühe (¼ l) ☐ nein ☐ ja

Zusätze: ☐ nein ☐ Kousmine ☐ Kousmine vegan
☐ Joghurt ☐ Haferschleim
☐ Reisschleim ☐ Karottensaft
☐ grüner Tee ☐ schwarzer Tee
☐ andere: ____________________

○ **Fastenbrechen**

○ **Aufbautag:** ☐ 1 ☐ 2 ☐ 3 ☐ 4

Körperliches Befinden:

sehr schlecht	0	1	2	3	4	5	6	7	8	9	10	hervorragend

Psychisch-seelisches Befinden:

sehr schlecht	0	1	2	3	4	5	6	7	8	9	10	hervorragend

In der Klinik neu aufgetretene Beschwerden: ☐ nein ☐ ja

- ☐ Kopfschmerzen
- ☐ Schlafstörungen
- ☐ Mundgeruch
  (fragen Sie die Schwester)
- ☐ Müdigkeit
- ☐ Rückenschmerzen
- ☐ Kälteempfindlichkeit
- ☐ Schwindel
- ☐ Hunger
- ☐ Hautausschlag
- ☐ Blähungen
- ☐ Muskelschmerzen
- ☐ Sodbrennen
- ☐ Übelkeit
- ☐ Trockener Mund
- ☐ Wadenkrämpfe
- ☐ Durchfall
- ☐ Unruhige Beine
- ☐ Obstipation
- ☐ Herzklopfen
- ☐ Sehstörungen
- ☐ Essdrang
- ☐ sonstige: ______________________________

**Am heutigen Tag…**	zu keinem Zeitpunkt	ab und zu	etwas weniger als die Hälfte der Zeit	etwas mehr als die Hälfte der Zeit	meistens	die ganze Zeit
…war ich froh und guter Laune	0	1	2	3	4	5
…habe ich mich ruhig und entspannt gefühlt	0	1	2	3	4	5
…habe ich mich energisch und aktiv gefühlt	0	1	2	3	4	5
…habe ich mich beim Aufwachen frisch und ausgeruht gefühlt	0	1	2	3	4	5
…war mein Alltag voller Dinge, die mich interessieren	0	1	2	3	4	5

4. Tag: ______________________

Ketonkörper (mg/dl): ☐ 0 ☐ 5 ☐ 15 ☐ 40 ☐ 80 ☐ 160

○ **Diät:** ☐ 800 kcal ☐ 1.200 kcal ☐ 1.800 kcal

Zusätze: ☐ nein ☐ Fisch
☐ andere: ______________

○ **Entlastungstag**: ☐ Reis ☐ Kartoffel/Gemüse
☐ Früchte ☐ andere: ________

○ **Fasten:**

	Honig	☐ nein	☐ ja
mittags:	Saft (¼ l)	☐ nein	☐ ja
	Gemüsebrühe (¼ l)	☐ nein	☐ ja
abends:	Gemüsebrühe (¼ l)	☐ nein	☐ ja

Zusätze: ☐ nein ☐ Kousmine ☐ Kousmine vegan
☐ Joghurt ☐ Haferschleim
☐ Reisschleim ☐ Karottensaft
☐ grüner Tee ☐ schwarzer Tee
☐ andere: ______________

○ **Fastenbrechen**

○ **Aufbautag:** ☐ 1 ☐ 2 ☐ 3 ☐ 4

Körperliches Befinden:

sehr schlecht	0	1	2	3	4	5	6	7	8	9	10	hervorragend

Psychisch-seelisches Befinden:

sehr schlecht	0	1	2	3	4	5	6	7	8	9	10	hervorragend

In der Klinik neu aufgetretene Beschwerden: ☐ nein ☐ ja

- ☐ Kopfschmerzen
- ☐ Schlafstörungen
- ☐ Mundgeruch (fragen Sie die Schwester)
- ☐ Müdigkeit
- ☐ Rückenschmerzen
- ☐ Kälteempfindlichkeit
- ☐ Schwindel
- ☐ Hunger
- ☐ Hautausschlag
- ☐ Blähungen
- ☐ Muskelschmerzen
- ☐ Sodbrennen
- ☐ Übelkeit
- ☐ Trockener Mund
- ☐ Wadenkrämpfe
- ☐ Durchfall
- ☐ Unruhige Beine
- ☐ Obstipation
- ☐ Herzklopfen
- ☐ Sehstörungen
- ☐ Essdrang
- ☐ sonstige: ____________________

**Am heutigen Tag…**	zu keinem Zeitpunkt	ab und zu	etwas weniger als die Hälfte der Zeit	etwas mehr als die Hälfte der Zeit	meistens	die ganze Zeit
…war ich froh und guter Laune	0	1	2	3	4	5
…habe ich mich ruhig und entspannt gefühlt	0	1	2	3	4	5
…habe ich mich energisch und aktiv gefühlt	0	1	2	3	4	5
…habe ich mich beim Aufwachen frisch und ausgeruht gefühlt	0	1	2	3	4	5
…war mein Alltag voller Dinge, die mich interessieren	0	1	2	3	4	5

5. Tag: ______________________________

Ketonkörper (mg/dl): ☐ 0 ☐ 5 ☐ 15 ☐ 40 ☐ 80 ☐ 160

○ **Diät:** ☐ 800 kcal ☐ 1.200 kcal ☐ 1.800 kcal

Zusätze: ☐ nein ☐ Fisch
☐ andere: ____________________

○ **Entlastungstag**: ☐ Reis ☐ Kartoffel/Gemüse
☐ Früchte ☐ andere: ________

○ **Fasten:** Honig ☐ nein ☐ ja

mittags: Saft (¼ l) ☐ nein ☐ ja
Gemüsebrühe (¼ l) ☐ nein ☐ ja
abends: Gemüsebrühe (¼ l) ☐ nein ☐ ja

Zusätze: ☐ nein ☐ Kousmine ☐ Kousmine vegan
☐ Joghurt ☐ Haferschleim
☐ Reisschleim ☐ Karottensaft
☐ grüner Tee ☐ schwarzer Tee
☐ andere: ____________________

○ **Fastenbrechen**

○ **Aufbautag:** ☐ 1 ☐ 2 ☐ 3 ☐ 4

Körperliches Befinden:

sehr schlecht	0	1	2	3	4	5	6	7	8	9	10	hervorragend

Psychisch-seelisches Befinden:

sehr schlecht	0	1	2	3	4	5	6	7	8	9	10	hervorragend

In der Klinik neu aufgetretene Beschwerden: ☐ nein ☐ ja

- ☐ Kopfschmerzen
- ☐ Schlafstörungen
- ☐ Mundgeruch
  (fragen Sie die Schwester)
- ☐ Müdigkeit
- ☐ Rückenschmerzen
- ☐ Kälteempfindlichkeit
- ☐ Schwindel
- ☐ Hunger
- ☐ Hautausschlag
- ☐ Blähungen
- ☐ Muskelschmerzen
- ☐ Sodbrennen
- ☐ Übelkeit
- ☐ Trockener Mund
- ☐ Wadenkrämpfe
- ☐ Durchfall
- ☐ Unruhige Beine
- ☐ Obstipation
- ☐ Herzklopfen
- ☐ Sehstörungen
- ☐ Essdrang
- ☐ sonstige: ____________________

**Am heutigen Tag...**	zu keinem Zeitpunkt	ab und zu	etwas weniger als die Hälfte der Zeit	etwas mehr als die Hälfte der Zeit	meistens	die ganze Zeit
...war ich froh und guter Laune	0	1	2	3	4	5
...habe ich mich ruhig und entspannt gefühlt	0	1	2	3	4	5
...habe ich mich energisch und aktiv gefühlt	0	1	2	3	4	5
...habe ich mich beim Aufwachen frisch und ausgeruht gefühlt	0	1	2	3	4	5
...war mein Alltag voller Dinge, die mich interessieren	0	1	2	3	4	5

6. Tag: ______________________________

Ketonkörper (mg/dl): ☐ 0 ☐ 5 ☐ 15 ☐ 40 ☐ 80 ☐ 160

○ **Diät:** ☐ 800 kcal ☐ 1.200 kcal ☐ 1.800 kcal

Zusätze: ☐ nein ☐ Fisch
☐ andere: ____________________

○ **Entlastungstag**: ☐ Reis ☐ Kartoffel/Gemüse
☐ Früchte ☐ andere: ________

○ **Fasten:** Honig ☐ nein ☐ ja

mittags: Saft (¼ l) ☐ nein ☐ ja
Gemüsebrühe (¼ l) ☐ nein ☐ ja
abends: Gemüsebrühe (¼ l) ☐ nein ☐ ja

Zusätze: ☐ nein ☐ Kousmine ☐ Kousmine vegan
☐ Joghurt ☐ Haferschleim
☐ Reisschleim ☐ Karottensaft
☐ grüner Tee ☐ schwarzer Tee
☐ andere: ____________________

○ **Fastenbrechen**

○ **Aufbautag:** ☐ 1 ☐ 2 ☐ 3 ☐ 4

Körperliches Befinden:

sehr schlecht	0	1	2	3	4	5	6	7	8	9	10	hervorragend

Psychisch-seelisches Befinden:

sehr schlecht	0	1	2	3	4	5	6	7	8	9	10	hervorragend

In der Klinik neu aufgetretene Beschwerden: ☐ nein ☐ ja

☐ Kopfschmerzen
☐ Schlafstörungen
☐ Mundgeruch
(fragen Sie die Schwester)
☐ Müdigkeit
☐ Rückenschmerzen
☐ Kälteempfindlichkeit
☐ Schwindel
☐ Hunger
☐ Hautausschlag
☐ Blähungen
☐ sonstige: ______________________

☐ Muskelschmerzen
☐ Sodbrennen
☐ Übelkeit
☐ Trockener Mund
☐ Wadenkrämpfe
☐ Durchfall
☐ Unruhige Beine
☐ Obstipation
☐ Herzklopfen
☐ Sehstörungen
☐ Essdrang

**Am heutigen Tag…**	zu keinem Zeitpunkt	ab und zu	etwas weniger als die Hälfte der Zeit	etwas mehr als die Hälfte der Zeit	meistens	die ganze Zeit
…war ich froh und guter Laune	0	1	2	3	4	5
…habe ich mich ruhig und entspannt gefühlt	0	1	2	3	4	5
…habe ich mich energisch und aktiv gefühlt	0	1	2	3	4	5
…habe ich mich beim Aufwachen frisch und ausgeruht gefühlt	0	1	2	3	4	5
…war mein Alltag voller Dinge, die mich interessieren	0	1	2	3	4	5

7. Tag: ______________________________

Ketonkörper (mg/dl): ☐ 0 ☐ 5 ☐ 15 ☐ 40 ☐ 80 ☐ 160

○ **Diät:** ☐ 800 kcal ☐ 1.200 kcal ☐ 1.800 kcal

Zusätze: ☐ nein ☐ Fisch
☐ andere: ____________________

○ **Entlastungstag**: ☐ Reis ☐ Kartoffel/Gemüse
☐ Früchte ☐ andere: ________

○ **Fasten:** Honig ☐ nein ☐ ja

mittags: Saft (¼ l) ☐ nein ☐ ja
Gemüsebrühe (¼ l) ☐ nein ☐ ja
abends: Gemüsebrühe (¼ l) ☐ nein ☐ ja

Zusätze: ☐ nein ☐ Kousmine ☐ Kousmine vegan
☐ Joghurt ☐ Haferschleim
☐ Reisschleim ☐ Karottensaft
☐ grüner Tee ☐ schwarzer Tee
☐ andere: ____________________

○ **Fastenbrechen**

○ **Aufbautag:** ☐ 1 ☐ 2 ☐ 3 ☐ 4

Körperliches Befinden:

sehr schlecht	0	1	2	3	4	5	6	7	8	9	10	hervorragend

Psychisch-seelisches Befinden:

sehr schlecht	0	1	2	3	4	5	6	7	8	9	10	hervorragend

In der Klinik neu aufgetretene Beschwerden: ☐ nein ☐ ja

☐ Kopfschmerzen
☐ Schlafstörungen
☐ Mundgeruch
(fragen Sie die Schwester)
☐ Müdigkeit
☐ Rückenschmerzen
☐ Kälteempfindlichkeit
☐ Schwindel
☐ Hunger
☐ Hautausschlag
☐ Blähungen
☐ Muskelschmerzen
☐ Sodbrennen
☐ Übelkeit
☐ Trockener Mund
☐ Wadenkrämpfe
☐ Durchfall
☐ Unruhige Beine
☐ Obstipation
☐ Herzklopfen
☐ Sehstörungen
☐ Essdrang
☐ sonstige: ____________________

**Am heutigen Tag…**	zu keinem Zeitpunkt	ab und zu	etwas weniger als die Hälfte der Zeit	etwas mehr als die Hälfte der Zeit	meistens	die ganze Zeit
…war ich froh und guter Laune	0	1	2	3	4	5
…habe ich mich ruhig und entspannt gefühlt	0	1	2	3	4	5
…habe ich mich energisch und aktiv gefühlt	0	1	2	3	4	5
…habe ich mich beim Aufwachen frisch und ausgeruht gefühlt	0	1	2	3	4	5
…war mein Alltag voller Dinge, die mich interessieren	0	1	2	3	4	5

8. Tag: ______________________________

Ketonkörper (mg/dl): ☐ 0 ☐ 5 ☐ 15 ☐ 40 ☐ 80 ☐ 160

○ **Diät:** ☐ 800 kcal ☐ 1.200 kcal ☐ 1.800 kcal

Zusätze: ☐ nein ☐ Fisch
☐ andere: ____________________

○ **Entlastungstag**: ☐ Reis ☐ Kartoffel/Gemüse
☐ Früchte ☐ andere: ________

○ **Fasten:**

	Honig	☐ nein	☐ ja
mittags:	Saft (¼ l)	☐ nein	☐ ja
	Gemüsebrühe (¼ l)	☐ nein	☐ ja
abends:	Gemüsebrühe (¼ l)	☐ nein	☐ ja

Zusätze: ☐ nein ☐ Kousmine ☐ Kousmine vegan
☐ Joghurt ☐ Haferschleim
☐ Reisschleim ☐ Karottensaft
☐ grüner Tee ☐ schwarzer Tee
☐ andere: ____________________

○ **Fastenbrechen**

○ **Aufbautag:** ☐ 1 ☐ 2 ☐ 3 ☐ 4

Körperliches Befinden:

sehr schlecht	0	1	2	3	4	5	6	7	8	9	10	hervorragend

Psychisch-seelisches Befinden:

sehr schlecht	0	1	2	3	4	5	6	7	8	9	10	hervorragend

In der Klinik neu aufgetretene Beschwerden: ☐ nein ☐ ja

- ☐ Kopfschmerzen
- ☐ Schlafstörungen
- ☐ Mundgeruch (fragen Sie die Schwester)
- ☐ Müdigkeit
- ☐ Rückenschmerzen
- ☐ Kälteempfindlichkeit
- ☐ Schwindel
- ☐ Hunger
- ☐ Hautausschlag
- ☐ Blähungen
- ☐ Muskelschmerzen
- ☐ Sodbrennen
- ☐ Übelkeit
- ☐ Trockener Mund
- ☐ Wadenkrämpfe
- ☐ Durchfall
- ☐ Unruhige Beine
- ☐ Obstipation
- ☐ Herzklopfen
- ☐ Sehstörungen
- ☐ Essdrang
- ☐ sonstige: ______________________

**Am heutigen Tag…**	zu keinem Zeitpunkt	ab und zu	etwas weniger als die Hälfte der Zeit	etwas mehr als die Hälfte der Zeit	meistens	die ganze Zeit
…war ich froh und guter Laune	0	1	2	3	4	5
…habe ich mich ruhig und entspannt gefühlt	0	1	2	3	4	5
…habe ich mich energisch und aktiv gefühlt	0	1	2	3	4	5
…habe ich mich beim Aufwachen frisch und ausgeruht gefühlt	0	1	2	3	4	5
…war mein Alltag voller Dinge, die mich interessieren	0	1	2	3	4	5

9. Tag: ______________________________

Ketonkörper (mg/dl): ☐ 0 ☐ 5 ☐ 15 ☐ 40 ☐ 80 ☐ 160

○ **Diät:** ☐ 800 kcal ☐ 1.200 kcal ☐ 1.800 kcal

Zusätze: ☐ nein ☐ Fisch
☐ andere: ______________________

○ **Entlastungstag**: ☐ Reis ☐ Kartoffel/Gemüse
☐ Früchte ☐ andere: __________

○ **Fasten:** Honig ☐ nein ☐ ja

mittags: Saft (¼ l) ☐ nein ☐ ja
Gemüsebrühe (¼ l) ☐ nein ☐ ja
abends: Gemüsebrühe (¼ l) ☐ nein ☐ ja

Zusätze: ☐ nein ☐ Kousmine ☐ Kousmine vegan
☐ Joghurt ☐ Haferschleim
☐ Reisschleim ☐ Karottensaft
☐ grüner Tee ☐ schwarzer Tee
☐ andere: ______________________

○ **Fastenbrechen**

○ **Aufbautag:** ☐ 1 ☐ 2 ☐ 3 ☐ 4

Körperliches Befinden:

sehr schlecht	0	1	2	3	4	5	6	7	8	9	10	hervorragend

Psychisch-seelisches Befinden:

sehr schlecht	0	1	2	3	4	5	6	7	8	9	10	hervorragend

In der Klinik neu aufgetretene Beschwerden: ☐ nein ☐ ja

- ☐ Kopfschmerzen
- ☐ Schlafstörungen
- ☐ Mundgeruch
  (fragen Sie die Schwester)
- ☐ Müdigkeit
- ☐ Rückenschmerzen
- ☐ Kälteempfindlichkeit
- ☐ Schwindel
- ☐ Hunger
- ☐ Hautausschlag
- ☐ Blähungen
- ☐ Muskelschmerzen
- ☐ Sodbrennen
- ☐ Übelkeit
- ☐ Trockener Mund
- ☐ Wadenkrämpfe
- ☐ Durchfall
- ☐ Unruhige Beine
- ☐ Obstipation
- ☐ Herzklopfen
- ☐ Sehstörungen
- ☐ Essdrang
- ☐ sonstige: ______________________

**Am heutigen Tag...**	zu keinem Zeitpunkt	ab und zu	etwas weniger als die Hälfte der Zeit	etwas mehr als die Hälfte der Zeit	meistens	die ganze Zeit
...war ich froh und guter Laune	0	1	2	3	4	5
...habe ich mich ruhig und entspannt gefühlt	0	1	2	3	4	5
...habe ich mich energisch und aktiv gefühlt	0	1	2	3	4	5
...habe ich mich beim Aufwachen frisch und ausgeruht gefühlt	0	1	2	3	4	5
...war mein Alltag voller Dinge, die mich interessieren	0	1	2	3	4	5

10. Tag: ____________________

Ketonkörper (mg/dl): ☐ 0 ☐ 5 ☐ 15 ☐ 40 ☐ 80 ☐ 160

○ **Diät:** ☐ 800 kcal ☐ 1.200 kcal ☐ 1.800 kcal

Zusätze: ☐ nein ☐ Fisch
☐ andere: ____________________

○ **Entlastungstag**: ☐ Reis ☐ Kartoffel/Gemüse
☐ Früchte ☐ andere: ________

○ **Fasten:** Honig ☐ nein ☐ ja

mittags: Saft (¼ l) ☐ nein ☐ ja
Gemüsebrühe (¼ l) ☐ nein ☐ ja
abends: Gemüsebrühe (¼ l) ☐ nein ☐ ja

Zusätze: ☐ nein ☐ Kousmine ☐ Kousmine vegan
☐ Joghurt ☐ Haferschleim
☐ Reisschleim ☐ Karottensaft
☐ grüner Tee ☐ schwarzer Tee
☐ andere: ____________________

○ **Fastenbrechen**

○ **Aufbautag:** ☐ 1 ☐ 2 ☐ 3 ☐ 4

Körperliches Befinden:

sehr schlecht	0	1	2	3	4	5	6	7	8	9	10	hervorragend

Psychisch-seelisches Befinden:

sehr schlecht	0	1	2	3	4	5	6	7	8	9	10	hervorragend

In der Klinik neu aufgetretene Beschwerden: ☐ nein ☐ ja

☐ Kopfschmerzen
☐ Schlafstörungen
☐ Mundgeruch
(fragen Sie die Schwester)
☐ Müdigkeit
☐ Rückenschmerzen
☐ Kälteempfindlichkeit
☐ Schwindel
☐ Hunger
☐ Hautausschlag
☐ Blähungen
☐ Muskelschmerzen
☐ Sodbrennen
☐ Übelkeit
☐ Trockener Mund
☐ Wadenkrämpfe
☐ Durchfall
☐ Unruhige Beine
☐ Obstipation
☐ Herzklopfen
☐ Sehstörungen
☐ Essdrang
☐ sonstige: ______________________________

**Am heutigen Tag…**	zu keinem Zeitpunkt	ab und zu	etwas weniger als die Hälfte der Zeit	etwas mehr als die Hälfte der Zeit	meistens	die ganze Zeit
…war ich froh und guter Laune	0	1	2	3	4	5
…habe ich mich ruhig und entspannt gefühlt	0	1	2	3	4	5
…habe ich mich energisch und aktiv gefühlt	0	1	2	3	4	5
…habe ich mich beim Aufwachen frisch und ausgeruht gefühlt	0	1	2	3	4	5
…war mein Alltag voller Dinge, die mich interessieren	0	1	2	3	4	5

11. Tag: ______________________________

Ketonkörper (mg/dl): ☐ 0 ☐ 5 ☐ 15 ☐ 40 ☐ 80 ☐ 160

○ **Diät:** ☐ 800 kcal ☐ 1.200 kcal ☐ 1.800 kcal

Zusätze: ☐ nein ☐ Fisch
☐ andere: ____________________

○ **Entlastungstag**: ☐ Reis ☐ Kartoffel/Gemüse
☐ Früchte ☐ andere: ________

○ **Fasten:** Honig ☐ nein ☐ ja

mittags: Saft (¼ l) ☐ nein ☐ ja
Gemüsebrühe (¼ l) ☐ nein ☐ ja
abends: Gemüsebrühe (¼ l) ☐ nein ☐ ja

Zusätze: ☐ nein ☐ Kousmine ☐ Kousmine vegan
☐ Joghurt ☐ Haferschleim
☐ Reisschleim ☐ Karottensaft
☐ grüner Tee ☐ schwarzer Tee
☐ andere: ____________________

○ **Fastenbrechen**

○ **Aufbautag:** ☐ 1 ☐ 2 ☐ 3 ☐ 4

Körperliches Befinden:

sehr schlecht | 0 | 1 | 2 | 3 | 4 | 5 | 6 | 7 | 8 | 9 | 10 | hervorragend

Psychisch-seelisches Befinden:

sehr schlecht | 0 | 1 | 2 | 3 | 4 | 5 | 6 | 7 | 8 | 9 | 10 | hervorragend

In der Klinik neu aufgetretene Beschwerden: ☐ nein ☐ ja

- ☐ Kopfschmerzen
- ☐ Schlafstörungen
- ☐ Mundgeruch
  (fragen Sie die Schwester)
- ☐ Müdigkeit
- ☐ Rückenschmerzen
- ☐ Kälteempfindlichkeit
- ☐ Schwindel
- ☐ Hunger
- ☐ Hautausschlag
- ☐ Blähungen
- ☐ Muskelschmerzen
- ☐ Sodbrennen
- ☐ Übelkeit
- ☐ Trockener Mund
- ☐ Wadenkrämpfe
- ☐ Durchfall
- ☐ Unruhige Beine
- ☐ Obstipation
- ☐ Herzklopfen
- ☐ Sehstörungen
- ☐ Essdrang
- ☐ sonstige: ____________________

**Am heutigen Tag…**	zu keinem Zeitpunkt	ab und zu	etwas weniger als die Hälfte der Zeit	etwas mehr als die Hälfte der Zeit	meistens	die ganze Zeit
…war ich froh und guter Laune	0	1	2	3	4	5
…habe ich mich ruhig und entspannt gefühlt	0	1	2	3	4	5
…habe ich mich energisch und aktiv gefühlt	0	1	2	3	4	5
…habe ich mich beim Aufwachen frisch und ausgeruht gefühlt	0	1	2	3	4	5
…war mein Alltag voller Dinge, die mich interessieren	0	1	2	3	4	5

12. Tag: ______________________________

Ketonkörper (mg/dl): ☐ 0 ☐ 5 ☐ 15 ☐ 40 ☐ 80 ☐ 160

○ **Diät:** ☐ 800 kcal ☐ 1.200 kcal ☐ 1.800 kcal

Zusätze: ☐ nein ☐ Fisch
☐ andere: ______________

○ **Entlastungstag:** ☐ Reis ☐ Kartoffel/Gemüse
☐ Früchte ☐ andere: ______

○ **Fasten:** Honig ☐ nein ☐ ja

mittags: Saft (¼ l) ☐ nein ☐ ja
Gemüsebrühe (¼ l) ☐ nein ☐ ja
abends: Gemüsebrühe (¼ l) ☐ nein ☐ ja

Zusätze: ☐ nein ☐ Kousmine ☐ Kousmine vegan
☐ Joghurt ☐ Haferschleim
☐ Reisschleim ☐ Karottensaft
☐ grüner Tee ☐ schwarzer Tee
☐ andere: ______________

○ **Fastenbrechen**

○ **Aufbautag:** ☐ 1 ☐ 2 ☐ 3 ☐ 4

Körperliches Befinden:

sehr schlecht	0	1	2	3	4	5	6	7	8	9	10	hervorragend

Psychisch-seelisches Befinden:

sehr schlecht	0	1	2	3	4	5	6	7	8	9	10	hervorragend

In der Klinik neu aufgetretene Beschwerden: ☐ nein ☐ ja

- ☐ Kopfschmerzen
- ☐ Schlafstörungen
- ☐ Mundgeruch (fragen Sie die Schwester)
- ☐ Müdigkeit
- ☐ Rückenschmerzen
- ☐ Kälteempfindlichkeit
- ☐ Schwindel
- ☐ Hunger
- ☐ Hautausschlag
- ☐ Blähungen
- ☐ Muskelschmerzen
- ☐ Sodbrennen
- ☐ Übelkeit
- ☐ Trockener Mund
- ☐ Wadenkrämpfe
- ☐ Durchfall
- ☐ Unruhige Beine
- ☐ Obstipation
- ☐ Herzklopfen
- ☐ Sehstörungen
- ☐ Essdrang
- ☐ sonstige: ____________________

**Am heutigen Tag…**	zu keinem Zeitpunkt	ab und zu	etwas weniger als die Hälfte der Zeit	etwas mehr als die Hälfte der Zeit	meistens	die ganze Zeit
…war ich froh und guter Laune	0	1	2	3	4	5
…habe ich mich ruhig und entspannt gefühlt	0	1	2	3	4	5
…habe ich mich energisch und aktiv gefühlt	0	1	2	3	4	5
…habe ich mich beim Aufwachen frisch und ausgeruht gefühlt	0	1	2	3	4	5
…war mein Alltag voller Dinge, die mich interessieren	0	1	2	3	4	5

13. Tag: ______________________________

Ketonkörper (mg/dl): ☐ 0 ☐ 5 ☐ 15 ☐ 40 ☐ 80 ☐ 160

○ **Diät:** ☐ 800 kcal ☐ 1.200 kcal ☐ 1.800 kcal

Zusätze: ☐ nein ☐ Fisch
☐ andere: ____________________

○ **Entlastungstag**: ☐ Reis ☐ Kartoffel/Gemüse
☐ Früchte ☐ andere: ________

○ **Fasten:** Honig ☐ nein ☐ ja

mittags: Saft (¼ l) ☐ nein ☐ ja
Gemüsebrühe (¼ l) ☐ nein ☐ ja
abends: Gemüsebrühe (¼ l) ☐ nein ☐ ja

Zusätze: ☐ nein ☐ Kousmine ☐ Kousmine vegan
☐ Joghurt ☐ Haferschleim
☐ Reisschleim ☐ Karottensaft
☐ grüner Tee ☐ schwarzer Tee
☐ andere: ____________________

○ **Fastenbrechen**

○ **Aufbautag:** ☐ 1 ☐ 2 ☐ 3 ☐ 4

Körperliches Befinden:

sehr schlecht	0	1	2	3	4	5	6	7	8	9	10	hervorragend

Psychisch-seelisches Befinden:

sehr schlecht	0	1	2	3	4	5	6	7	8	9	10	hervorragend

In der Klinik neu aufgetretene Beschwerden: ☐ nein ☐ ja

- ☐ Kopfschmerzen
- ☐ Schlafstörungen
- ☐ Mundgeruch (fragen Sie die Schwester)
- ☐ Müdigkeit
- ☐ Rückenschmerzen
- ☐ Kälteempfindlichkeit
- ☐ Schwindel
- ☐ Hunger
- ☐ Hautausschlag
- ☐ Blähungen
- ☐ Muskelschmerzen
- ☐ Sodbrennen
- ☐ Übelkeit
- ☐ Trockener Mund
- ☐ Wadenkrämpfe
- ☐ Durchfall
- ☐ Unruhige Beine
- ☐ Obstipation
- ☐ Herzklopfen
- ☐ Sehstörungen
- ☐ Essdrang
- ☐ sonstige: ____________

**Am heutigen Tag…**	zu keinem Zeitpunkt	ab und zu	etwas weniger als die Hälfte der Zeit	etwas mehr als die Hälfte der Zeit	meistens	die ganze Zeit
…war ich froh und guter Laune	0	1	2	3	4	5
…habe ich mich ruhig und entspannt gefühlt	0	1	2	3	4	5
…habe ich mich energisch und aktiv gefühlt	0	1	2	3	4	5
…habe ich mich beim Aufwachen frisch und ausgeruht gefühlt	0	1	2	3	4	5
…war mein Alltag voller Dinge, die mich interessieren	0	1	2	3	4	5

14. Tag: ______________________________

Ketonkörper (mg/dl): ☐ 0 ☐ 5 ☐ 15 ☐ 40 ☐ 80 ☐ 160

○ **Diät:** ☐ 800 kcal ☐ 1.200 kcal ☐ 1.800 kcal

Zusätze: ☐ nein ☐ Fisch
☐ andere: ______________________

○ **Entlastungstag**: ☐ Reis ☐ Kartoffel/Gemüse
☐ Früchte ☐ andere: __________

○ **Fasten:** Honig ☐ nein ☐ ja

mittags: Saft (¼ l) ☐ nein ☐ ja
Gemüsebrühe (¼ l) ☐ nein ☐ ja
abends: Gemüsebrühe (¼ l) ☐ nein ☐ ja

Zusätze: ☐ nein ☐ Kousmine ☐ Kousmine vegan
☐ Joghurt ☐ Haferschleim
☐ Reisschleim ☐ Karottensaft
☐ grüner Tee ☐ schwarzer Tee
☐ andere: ______________________

○ **Fastenbrechen**

○ **Aufbautag:** ☐ 1 ☐ 2 ☐ 3 ☐ 4

Körperliches Befinden:

sehr schlecht	0	1	2	3	4	5	6	7	8	9	10	hervorragend

Psychisch-seelisches Befinden:

sehr schlecht	0	1	2	3	4	5	6	7	8	9	10	hervorragend

In der Klinik neu aufgetretene Beschwerden: ☐ nein ☐ ja

☐ Kopfschmerzen
☐ Schlafstörungen
☐ Mundgeruch
(fragen Sie die Schwester)
☐ Müdigkeit
☐ Rückenschmerzen
☐ Kälteempfindlichkeit
☐ Schwindel
☐ Hunger
☐ Hautausschlag
☐ Blähungen
☐ Muskelschmerzen
☐ Sodbrennen
☐ Übelkeit
☐ Trockener Mund
☐ Wadenkrämpfe
☐ Durchfall
☐ Unruhige Beine
☐ Obstipation
☐ Herzklopfen
☐ Sehstörungen
☐ Essdrang
☐ sonstige: ____________________

**Am heutigen Tag…**	zu keinem Zeitpunkt	ab und zu	etwas weniger als die Hälfte der Zeit	etwas mehr als die Hälfte der Zeit	meistens	die ganze Zeit
…war ich froh und guter Laune	0	1	2	3	4	5
…habe ich mich ruhig und entspannt gefühlt	0	1	2	3	4	5
…habe ich mich energisch und aktiv gefühlt	0	1	2	3	4	5
…habe ich mich beim Aufwachen frisch und ausgeruht gefühlt	0	1	2	3	4	5
…war mein Alltag voller Dinge, die mich interessieren	0	1	2	3	4	5

15. Tag: ______________________________

Ketonkörper (mg/dl): ☐ 0 ☐ 5 ☐ 15 ☐ 40 ☐ 80 ☐ 160

○ **Diät:** ☐ 800 kcal ☐ 1.200 kcal ☐ 1.800 kcal

Zusätze: ☐ nein ☐ Fisch
☐ andere: ____________________

○ **Entlastungstag**: ☐ Reis ☐ Kartoffel/Gemüse
☐ Früchte ☐ andere: ________

○ **Fasten:** Honig ☐ nein ☐ ja

mittags: Saft (¼ l) ☐ nein ☐ ja
Gemüsebrühe (¼ l) ☐ nein ☐ ja
abends: Gemüsebrühe (¼ l) ☐ nein ☐ ja

Zusätze: ☐ nein ☐ Kousmine ☐ Kousmine vegan
☐ Joghurt ☐ Haferschleim
☐ Reisschleim ☐ Karottensaft
☐ grüner Tee ☐ schwarzer Tee
☐ andere: ____________________

○ **Fastenbrechen**

○ **Aufbautag:** ☐ 1 ☐ 2 ☐ 3 ☐ 4

Körperliches Befinden:

sehr schlecht	0	1	2	3	4	5	6	7	8	9	10	hervorragend

Psychisch-seelisches Befinden:

sehr schlecht	0	1	2	3	4	5	6	7	8	9	10	hervorragend

In der Klinik neu aufgetretene Beschwerden: ☐ nein ☐ ja

- ☐ Kopfschmerzen
- ☐ Schlafstörungen
- ☐ Mundgeruch
  (fragen Sie die Schwester)
- ☐ Müdigkeit
- ☐ Rückenschmerzen
- ☐ Kälteempfindlichkeit
- ☐ Schwindel
- ☐ Hunger
- ☐ Hautausschlag
- ☐ Blähungen
- ☐ Muskelschmerzen
- ☐ Sodbrennen
- ☐ Übelkeit
- ☐ Trockener Mund
- ☐ Wadenkrämpfe
- ☐ Durchfall
- ☐ Unruhige Beine
- ☐ Obstipation
- ☐ Herzklopfen
- ☐ Sehstörungen
- ☐ Essdrang
- ☐ sonstige: ______________________

**Am heutigen Tag…**	zu keinem Zeitpunkt	ab und zu	etwas weniger als die Hälfte der Zeit	etwas mehr als die Hälfte der Zeit	meistens	die ganze Zeit
…war ich froh und guter Laune	0	1	2	3	4	5
…habe ich mich ruhig und entspannt gefühlt	0	1	2	3	4	5
…habe ich mich energisch und aktiv gefühlt	0	1	2	3	4	5
…habe ich mich beim Aufwachen frisch und ausgeruht gefühlt	0	1	2	3	4	5
…war mein Alltag voller Dinge, die mich interessieren	0	1	2	3	4	5

16. Tag: ______________________

Ketonkörper (mg/dl): ☐ 0 ☐ 5 ☐ 15 ☐ 40 ☐ 80 ☐ 160

○ **Diät:** ☐ 800 kcal ☐ 1.200 kcal ☐ 1.800 kcal

Zusätze: ☐ nein ☐ Fisch
☐ andere: ______________

○ **Entlastungstag**: ☐ Reis ☐ Kartoffel/Gemüse
☐ Früchte ☐ andere: ________

○ **Fasten:** Honig ☐ nein ☐ ja

mittags: Saft (¼ l) ☐ nein ☐ ja
Gemüsebrühe (¼ l) ☐ nein ☐ ja
abends: Gemüsebrühe (¼ l) ☐ nein ☐ ja

Zusätze: ☐ nein ☐ Kousmine ☐ Kousmine vegan
☐ Joghurt ☐ Haferschleim
☐ Reisschleim ☐ Karottensaft
☐ grüner Tee ☐ schwarzer Tee
☐ andere: ______________

○ **Fastenbrechen**

○ **Aufbautag:** ☐ 1 ☐ 2 ☐ 3 ☐ 4

Körperliches Befinden:

sehr schlecht	0	1	2	3	4	5	6	7	8	9	10	hervorragend

Psychisch-seelisches Befinden:

sehr schlecht	0	1	2	3	4	5	6	7	8	9	10	hervorragend

In der Klinik neu aufgetretene Beschwerden: ☐ nein ☐ ja

- ☐ Kopfschmerzen
- ☐ Schlafstörungen
- ☐ Mundgeruch (fragen Sie die Schwester)
- ☐ Müdigkeit
- ☐ Rückenschmerzen
- ☐ Kälteempfindlichkeit
- ☐ Schwindel
- ☐ Hunger
- ☐ Hautausschlag
- ☐ Blähungen
- ☐ Muskelschmerzen
- ☐ Sodbrennen
- ☐ Übelkeit
- ☐ Trockener Mund
- ☐ Wadenkrämpfe
- ☐ Durchfall
- ☐ Unruhige Beine
- ☐ Obstipation
- ☐ Herzklopfen
- ☐ Sehstörungen
- ☐ Essdrang
- ☐ sonstige: ______________________________

**Am heutigen Tag…**	zu keinem Zeitpunkt	ab und zu	etwas weniger als die Hälfte der Zeit	etwas mehr als die Hälfte der Zeit	meistens	die ganze Zeit
…war ich froh und guter Laune	0	1	2	3	4	5
…habe ich mich ruhig und entspannt gefühlt	0	1	2	3	4	5
…habe ich mich energisch und aktiv gefühlt	0	1	2	3	4	5
…habe ich mich beim Aufwachen frisch und ausgeruht gefühlt	0	1	2	3	4	5
…war mein Alltag voller Dinge, die mich interessieren	0	1	2	3	4	5

17. Tag: ______________________________

Ketonkörper (mg/dl): ☐ 0 ☐ 5 ☐ 15 ☐ 40 ☐ 80 ☐ 160

○ **Diät:** ☐ 800 kcal ☐ 1.200 kcal ☐ 1.800 kcal

Zusätze: ☐ nein ☐ Fisch
☐ andere: ____________________

○ **Entlastungstag**: ☐ Reis ☐ Kartoffel/Gemüse
☐ Früchte ☐ andere: ________

○ **Fasten:** Honig ☐ nein ☐ ja

mittags: Saft (¼ l) ☐ nein ☐ ja
Gemüsebrühe (¼ l) ☐ nein ☐ ja
abends: Gemüsebrühe (¼ l) ☐ nein ☐ ja

Zusätze: ☐ nein ☐ Kousmine ☐ Kousmine vegan
☐ Joghurt ☐ Haferschleim
☐ Reisschleim ☐ Karottensaft
☐ grüner Tee ☐ schwarzer Tee
☐ andere: ____________________

○ **Fastenbrechen**

○ **Aufbautag:** ☐ 1 ☐ 2 ☐ 3 ☐ 4

Körperliches Befinden:

sehr schlecht	0	1	2	3	4	5	6	7	8	9	10	hervorragend

Psychisch-seelisches Befinden:

sehr schlecht	0	1	2	3	4	5	6	7	8	9	10	hervorragend

In der Klinik neu aufgetretene Beschwerden: ☐ nein ☐ ja

- ☐ Kopfschmerzen
- ☐ Schlafstörungen
- ☐ Mundgeruch
  (fragen Sie die Schwester)
- ☐ Müdigkeit
- ☐ Rückenschmerzen
- ☐ Kälteempfindlichkeit
- ☐ Schwindel
- ☐ Hunger
- ☐ Hautausschlag
- ☐ Blähungen
- ☐ Muskelschmerzen
- ☐ Sodbrennen
- ☐ Übelkeit
- ☐ Trockener Mund
- ☐ Wadenkrämpfe
- ☐ Durchfall
- ☐ Unruhige Beine
- ☐ Obstipation
- ☐ Herzklopfen
- ☐ Sehstörungen
- ☐ Essdrang
- ☐ sonstige: ____________________

**Am heutigen Tag…**	zu keinem Zeitpunkt	ab und zu	etwas weniger als die Hälfte der Zeit	etwas mehr als die Hälfte der Zeit	meistens	die ganze Zeit
…war ich froh und guter Laune	0	1	2	3	4	5
…habe ich mich ruhig und entspannt gefühlt	0	1	2	3	4	5
…habe ich mich energisch und aktiv gefühlt	0	1	2	3	4	5
…habe ich mich beim Aufwachen frisch und ausgeruht gefühlt	0	1	2	3	4	5
…war mein Alltag voller Dinge, die mich interessieren	0	1	2	3	4	5

18. Tag: ____________________

Ketonkörper (mg/dl): ☐ 0 ☐ 5 ☐ 15 ☐ 40 ☐ 80 ☐ 160

○ **Diät:** ☐ 800 kcal ☐ 1.200 kcal ☐ 1.800 kcal

Zusätze: ☐ nein ☐ Fisch
☐ andere: ____________________

○ **Entlastungstag**: ☐ Reis ☐ Kartoffel/Gemüse
☐ Früchte ☐ andere: __________

○ **Fasten:** Honig ☐ nein ☐ ja

mittags: Saft (¼ l) ☐ nein ☐ ja
Gemüsebrühe (¼ l) ☐ nein ☐ ja
abends: Gemüsebrühe (¼ l) ☐ nein ☐ ja

Zusätze: ☐ nein ☐ Kousmine ☐ Kousmine vegan
☐ Joghurt ☐ Haferschleim
☐ Reisschleim ☐ Karottensaft
☐ grüner Tee ☐ schwarzer Tee
☐ andere: ____________________

○ **Fastenbrechen**

○ **Aufbautag:** ☐ 1 ☐ 2 ☐ 3 ☐ 4

Körperliches Befinden:

sehr schlecht	0	1	2	3	4	5	6	7	8	9	10	hervorragend

Psychisch-seelisches Befinden:

sehr schlecht	0	1	2	3	4	5	6	7	8	9	10	hervorragend

In der Klinik neu aufgetretene Beschwerden: ☐ nein ☐ ja

☐ Kopfschmerzen
☐ Schlafstörungen
☐ Mundgeruch
(fragen Sie die Schwester)
☐ Müdigkeit
☐ Rückenschmerzen
☐ Kälteempfindlichkeit
☐ Schwindel
☐ Hunger
☐ Hautausschlag
☐ Blähungen
☐ Muskelschmerzen
☐ Sodbrennen
☐ Übelkeit
☐ Trockener Mund
☐ Wadenkrämpfe
☐ Durchfall
☐ Unruhige Beine
☐ Obstipation
☐ Herzklopfen
☐ Sehstörungen
☐ Essdrang
☐ sonstige: ______________________

**Am heutigen Tag…**	zu keinem Zeitpunkt	ab und zu	etwas weniger als die Hälfte der Zeit	etwas mehr als die Hälfte der Zeit	meistens	die ganze Zeit
…war ich froh und guter Laune	0	1	2	3	4	5
…habe ich mich ruhig und entspannt gefühlt	0	1	2	3	4	5
…habe ich mich energisch und aktiv gefühlt	0	1	2	3	4	5
…habe ich mich beim Aufwachen frisch und ausgeruht gefühlt	0	1	2	3	4	5
…war mein Alltag voller Dinge, die mich interessieren	0	1	2	3	4	5

19. Tag: ____________________

Ketonkörper (mg/dl): ☐ 0 ☐ 5 ☐ 15 ☐ 40 ☐ 80 ☐ 160

○ **Diät:** ☐ 800 kcal ☐ 1.200 kcal ☐ 1.800 kcal

Zusätze: ☐ nein ☐ Fisch
☐ andere: ____________________

○ **Entlastungstag**: ☐ Reis ☐ Kartoffel/Gemüse
☐ Früchte ☐ andere: __________

○ **Fasten:** Honig ☐ nein ☐ ja

mittags: Saft (¼ l) ☐ nein ☐ ja
Gemüsebrühe (¼ l) ☐ nein ☐ ja
abends: Gemüsebrühe (¼ l) ☐ nein ☐ ja

Zusätze: ☐ nein ☐ Kousmine ☐ Kousmine vegan
☐ Joghurt ☐ Haferschleim
☐ Reisschleim ☐ Karottensaft
☐ grüner Tee ☐ schwarzer Tee
☐ andere: ____________________

○ **Fastenbrechen**

○ **Aufbautag:** ☐ 1 ☐ 2 ☐ 3 ☐ 4

Körperliches Befinden:

sehr schlecht	0	1	2	3	4	5	6	7	8	9	10	hervorragend

Psychisch-seelisches Befinden:

sehr schlecht	0	1	2	3	4	5	6	7	8	9	10	hervorragend

In der Klinik neu aufgetretene Beschwerden: ☐ nein ☐ ja

- ☐ Kopfschmerzen
- ☐ Schlafstörungen
- ☐ Mundgeruch
  (fragen Sie die Schwester)
- ☐ Müdigkeit
- ☐ Rückenschmerzen
- ☐ Kälteempfindlichkeit
- ☐ Schwindel
- ☐ Hunger
- ☐ Hautausschlag
- ☐ Blähungen
- ☐ Muskelschmerzen
- ☐ Sodbrennen
- ☐ Übelkeit
- ☐ Trockener Mund
- ☐ Wadenkrämpfe
- ☐ Durchfall
- ☐ Unruhige Beine
- ☐ Obstipation
- ☐ Herzklopfen
- ☐ Sehstörungen
- ☐ Essdrang
- ☐ sonstige: ______________________

**Am heutigen Tag...**	zu keinem Zeitpunkt	ab und zu	etwas weniger als die Hälfte der Zeit	etwas mehr als die Hälfte der Zeit	meistens	die ganze Zeit
...war ich froh und guter Laune	0	1	2	3	4	5
...habe ich mich ruhig und entspannt gefühlt	0	1	2	3	4	5
...habe ich mich energisch und aktiv gefühlt	0	1	2	3	4	5
...habe ich mich beim Aufwachen frisch und ausgeruht gefühlt	0	1	2	3	4	5
...war mein Alltag voller Dinge, die mich interessieren	0	1	2	3	4	5

20. Tag: ______________________

Ketonkörper (mg/dl): ☐ 0 ☐ 5 ☐ 15 ☐ 40 ☐ 80 ☐ 160

○ **Diät:** ☐ 800 kcal ☐ 1.200 kcal ☐ 1.800 kcal

Zusätze: ☐ nein ☐ Fisch
☐ andere: ______________

○ **Entlastungstag**: ☐ Reis ☐ Kartoffel/Gemüse
☐ Früchte ☐ andere: ______

○ **Fasten:**

	Honig	☐ nein	☐ ja
mittags:	Saft (¼ l)	☐ nein	☐ ja
	Gemüsebrühe (¼ l)	☐ nein	☐ ja
abends:	Gemüsebrühe (¼ l)	☐ nein	☐ ja

Zusätze: ☐ nein ☐ Kousmine ☐ Kousmine vegan
☐ Joghurt ☐ Haferschleim
☐ Reisschleim ☐ Karottensaft
☐ grüner Tee ☐ schwarzer Tee
☐ andere: ______________

○ **Fastenbrechen**

○ **Aufbautag:** ☐ 1 ☐ 2 ☐ 3 ☐ 4

Körperliches Befinden:

sehr schlecht	0	1	2	3	4	5	6	7	8	9	10	hervorragend

Psychisch-seelisches Befinden:

sehr schlecht	0	1	2	3	4	5	6	7	8	9	10	hervorragend

In der Klinik neu aufgetretene Beschwerden: ☐ nein ☐ ja

- ☐ Kopfschmerzen
- ☐ Schlafstörungen
- ☐ Mundgeruch (fragen Sie die Schwester)
- ☐ Müdigkeit
- ☐ Rückenschmerzen
- ☐ Kälteempfindlichkeit
- ☐ Schwindel
- ☐ Hunger
- ☐ Hautausschlag
- ☐ Blähungen
- ☐ Muskelschmerzen
- ☐ Sodbrennen
- ☐ Übelkeit
- ☐ Trockener Mund
- ☐ Wadenkrämpfe
- ☐ Durchfall
- ☐ Unruhige Beine
- ☐ Obstipation
- ☐ Herzklopfen
- ☐ Sehstörungen
- ☐ Essdrang
- ☐ sonstige: ____________________

**Am heutigen Tag…**	zu keinem Zeitpunkt	ab und zu	etwas weniger als die Hälfte der Zeit	etwas mehr als die Hälfte der Zeit	meistens	die ganze Zeit
…war ich froh und guter Laune	0	1	2	3	4	5
…habe ich mich ruhig und entspannt gefühlt	0	1	2	3	4	5
…habe ich mich energisch und aktiv gefühlt	0	1	2	3	4	5
…habe ich mich beim Aufwachen frisch und ausgeruht gefühlt	0	1	2	3	4	5
…war mein Alltag voller Dinge, die mich interessieren	0	1	2	3	4	5

21. Tag: ____________________

Ketonkörper (mg/dl): ☐ 0 ☐ 5 ☐ 15 ☐ 40 ☐ 80 ☐ 160

○ **Diät:** ☐ 800 kcal ☐ 1.200 kcal ☐ 1.800 kcal

Zusätze: ☐ nein ☐ Fisch
☐ andere: ____________________

○ **Entlastungstag**: ☐ Reis ☐ Kartoffel/Gemüse
☐ Früchte ☐ andere: __________

○ **Fasten:** Honig ☐ nein ☐ ja

mittags: Saft (¼ l) ☐ nein ☐ ja
Gemüsebrühe (¼ l) ☐ nein ☐ ja
abends: Gemüsebrühe (¼ l) ☐ nein ☐ ja

Zusätze: ☐ nein ☐ Kousmine ☐ Kousmine vegan
☐ Joghurt ☐ Haferschleim
☐ Reisschleim ☐ Karottensaft
☐ grüner Tee ☐ schwarzer Tee
☐ andere: ____________________

○ **Fastenbrechen**

○ **Aufbautag:** ☐ 1 ☐ 2 ☐ 3 ☐ 4

Körperliches Befinden:

sehr schlecht	0	1	2	3	4	5	6	7	8	9	10	hervorragend

Psychisch-seelisches Befinden:

sehr schlecht	0	1	2	3	4	5	6	7	8	9	10	hervorragend

In der Klinik neu aufgetretene Beschwerden: ☐ nein ☐ ja

- ☐ Kopfschmerzen
- ☐ Schlafstörungen
- ☐ Mundgeruch (fragen Sie die Schwester)
- ☐ Müdigkeit
- ☐ Rückenschmerzen
- ☐ Kälteempfindlichkeit
- ☐ Schwindel
- ☐ Hunger
- ☐ Hautausschlag
- ☐ Blähungen
- ☐ Muskelschmerzen
- ☐ Sodbrennen
- ☐ Übelkeit
- ☐ Trockener Mund
- ☐ Wadenkrämpfe
- ☐ Durchfall
- ☐ Unruhige Beine
- ☐ Obstipation
- ☐ Herzklopfen
- ☐ Sehstörungen
- ☐ Essdrang
- ☐ sonstige: ______________________

**Am heutigen Tag…**	zu keinem Zeitpunkt	ab und zu	etwas weniger als die Hälfte der Zeit	etwas mehr als die Hälfte der Zeit	meistens	die ganze Zeit
…war ich froh und guter Laune	0	1	2	3	4	5
…habe ich mich ruhig und entspannt gefühlt	0	1	2	3	4	5
…habe ich mich energisch und aktiv gefühlt	0	1	2	3	4	5
…habe ich mich beim Aufwachen frisch und ausgeruht gefühlt	0	1	2	3	4	5
…war mein Alltag voller Dinge, die mich interessieren	0	1	2	3	4	5

22. Tag: ______________________________

Ketonkörper (mg/dl): ☐ 0 ☐ 5 ☐ 15 ☐ 40 ☐ 80 ☐ 160

○ **Diät:** ☐ 800 kcal ☐ 1.200 kcal ☐ 1.800 kcal

Zusätze: ☐ nein ☐ Fisch
☐ andere: ______________________

○ **Entlastungstag**: ☐ Reis ☐ Kartoffel/Gemüse
☐ Früchte ☐ andere: __________

○ **Fasten:**

	Honig	☐ nein	☐ ja
mittags:	Saft (¼ l)	☐ nein	☐ ja
	Gemüsebrühe (¼ l)	☐ nein	☐ ja
abends:	Gemüsebrühe (¼ l)	☐ nein	☐ ja

Zusätze: ☐ nein ☐ Kousmine ☐ Kousmine vegan
☐ Joghurt ☐ Haferschleim
☐ Reisschleim ☐ Karottensaft
☐ grüner Tee ☐ schwarzer Tee
☐ andere: ______________________

○ **Fastenbrechen**

○ **Aufbautag:** ☐ 1 ☐ 2 ☐ 3 ☐ 4

Körperliches Befinden:

sehr schlecht	0	1	2	3	4	5	6	7	8	9	10	hervorragend

Psychisch-seelisches Befinden:

sehr schlecht	0	1	2	3	4	5	6	7	8	9	10	hervorragend

In der Klinik neu aufgetretene Beschwerden: ☐ nein ☐ ja

- ☐ Kopfschmerzen
- ☐ Schlafstörungen
- ☐ Mundgeruch
  (fragen Sie die Schwester)
- ☐ Müdigkeit
- ☐ Rückenschmerzen
- ☐ Kälteempfindlichkeit
- ☐ Schwindel
- ☐ Hunger
- ☐ Hautausschlag
- ☐ Blähungen
- ☐ Muskelschmerzen
- ☐ Sodbrennen
- ☐ Übelkeit
- ☐ Trockener Mund
- ☐ Wadenkrämpfe
- ☐ Durchfall
- ☐ Unruhige Beine
- ☐ Obstipation
- ☐ Herzklopfen
- ☐ Sehstörungen
- ☐ Essdrang
- ☐ sonstige: ____________________

**Am heutigen Tag…**	zu keinem Zeitpunkt	ab und zu	etwas weniger als die Hälfte der Zeit	etwas mehr als die Hälfte der Zeit	meistens	die ganze Zeit
…war ich froh und guter Laune	0	1	2	3	4	5
…habe ich mich ruhig und entspannt gefühlt	0	1	2	3	4	5
…habe ich mich energisch und aktiv gefühlt	0	1	2	3	4	5
…habe ich mich beim Aufwachen frisch und ausgeruht gefühlt	0	1	2	3	4	5
…war mein Alltag voller Dinge, die mich interessieren	0	1	2	3	4	5

Fragen am Ende des Aufenthalts

1) Hatten Sie zu Beginn des Aufenthalts eine gesundheitliche Hauptbeschwerde?

   ☐ nein

   ☐ ja, welche? ______________________________

   ____

   Wie hat sich diese seit Aufenthaltsbeginn zu heute verändert?

sehr viel schlechter	1	2	3	4	5	6	7	sehr viel besser

2) Sie haben sich während des Aufenthalts einer Fastentherapie bzw. einer Diät unterzogen.
   Wie würden Sie diese bewerten?

es war fast nicht durchzuhalten	1	2	3	4	5	6	7	ein großartiges Erlebnis

3) Wenn Sie Ihren Aufenthalt bei Buchinger Wilhelmi mit einer üblichen Urlaubsreise oder einem anderen Erholungsurlaub vergleichen, war dieser:

   ☐ weniger erholsam ☐ gleich erholsam ☐ stärker erholsam

4) Die Länge Ihres Aufenthalts empfanden Sie als:

   ☐ zu kurz ☐ genau richtig ☐ zu lang

5) Falls Sie gefastet haben, hätten Sie am letzten Fastentag gerne länger gefastet?

   ☐ nein ☐ ja

Lebensstil – während des Aufenthalts

Stress-/Entspannungsniveau:

bedrohliche Anspannung	0	1	2	3	4	5	6	7	8	9	10	total entspannt

**Nikotin:**	☐ nein	☐ Zigaretten/Tag:	
	☐ nein	☐ Zigarren/Tag:	
**Alkohol:**	☐ nein	☐ Bier: Gläser (0,5 l)/Woche:	
	☐ nein	☐ Wein: Gläser (0,25 l)/Woche:	
	☐ nein	☐ Schnaps: Gläser (2 cl)/Woche:	
**Kaffee:**	☐ nein	☐ Tassen/Tag:	
**Medien:**	☐ nein	☐ Smartphone (h/Woche):	
	☐ nein	☐ TV (h/Woche):	
	☐ nein	☐ PC (h/Woche):	
**Sport/ Bewegung:**	☐ nein	☐ ja: h/Woche:	,

**In den letzten Wochen…**	zu keinem Zeitpunkt	ab und zu	etwas weniger als die Hälfte der Zeit	etwas mehr als die Hälfte der Zeit	meistens	die ganze Zeit
…war ich froh und guter Laune	0	1	2	3	4	5
…habe ich mich ruhig und entspannt gefühlt	0	1	2	3	4	5
…habe ich mich energisch und aktiv gefühlt	0	1	2	3	4	5
…habe ich mich beim Aufwachen frisch und ausgeruht gefühlt	0	1	2	3	4	5
…war mein Alltag voller Dinge, die mich interessieren	0	1	2	3	4	5

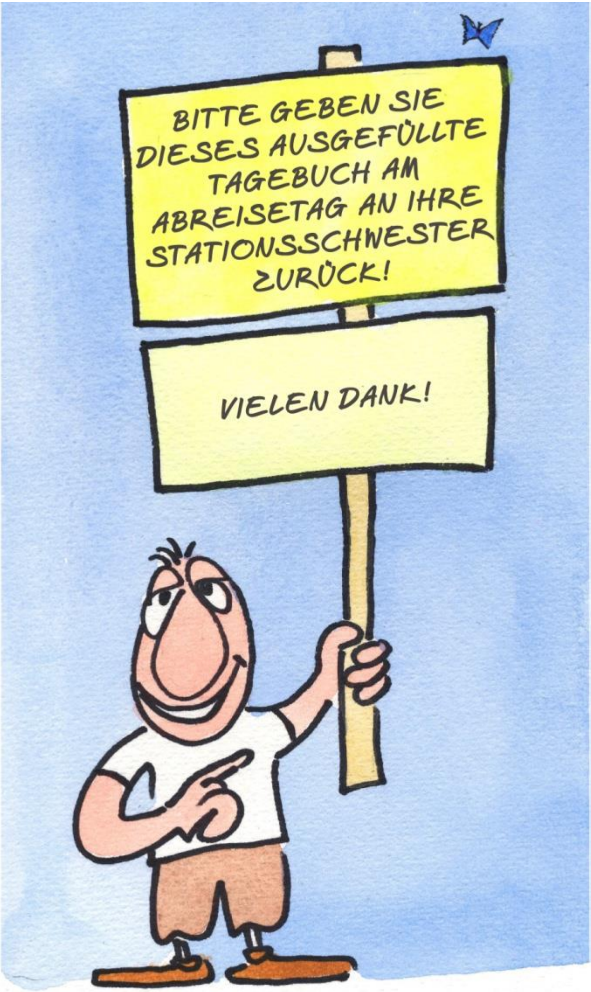
BITTE GEBEN SIE DIESES AUSGEFÜLLTE TAGEBUCH AM ABREISETAG AN IHRE STATIONSSCHWESTER ZURÜCK!
VIELEN DANK!

Die Beantwortung der folgenden Fragen ist für die wissenschaftliche Dokumentation nicht erforderlich, hilft jedoch der Klinik Buchinger Wilhelmi ihr Angebot immer besser zu gestalten:

Wie empfanden Sie das Ausfüllen dieses Heftchens?

Hätten Sie Verbesserungsvorschläge?

Hat der Aufenthalt Ihre Erwartungen erfüllt?

Welchen Einfluss, glauben Sie, wird der Aufenthalt auf Ihr Leben haben?

Brauchen Sie die Unterstützung einer Ernährungsberaterin im Alltag? Hier unser Skype-Angebot: „Feel well after Buchinger Wilhelmi“
E-Mail: nutrition@buchinger-wilhelmi.com

Vielen Dank und auf Wiedersehen!

Wir freuen uns, Sie in zwei Wochen, drei und elf Monaten anzuschreiben, um zu erfahren wie es Ihnen geht.

Gute Heimfahrt!
Follow up Buchinger Wilhelmi
