# Supplementary material for: Safety, health improvement and well-being during a 4 to 21-day fasting period in an observational study including 1422 subjects
Source: PLoS One. 2019 Jan 2;14(1):e0209353. doi: 10.1371/journal.pone.0209353 (PMC6314618; doi:10.1371/journal.pone.0209353)
Supplement: S5 Text — (PDF) [file pone.0209353.s022.pdf]

S5 Text. Questionnaires in English.

Self-observation

diary

Name: ______________________________

Room number: ______________________________

Stay from: ______________ to: ______________

Dear patients,
dear guests

The changes in metabolism caused by fasting or nutritional therapy could affect the personal well-being. By using this diary the effects could be recorded and thus become more comprehensible. In addition it could help to optimize prospective therapy recommendations.

Only a complete documentation enables us to detect coherences. Therefore, we kindly ask you to complete this diary as accurately as possible.

In addition we request you to fill in information about:

1. your lifestyle before the stay (page 7)
2. your lifestyle during the stay (page 53)
3. questions at the end of your stay (page 52)

Please give the completed diary back to your nurse on the day of departure.

Thank you for your participation!

Dr Françoise Wilhelmi de Toledo and
the scientific team of the clinic Buchinger Wilhelmi

Instruction:

- Date:
  First enter the date, please. Day 1 is equivalent to the day of arrival.

- Diet:
  Please mark your accurate diet with a cross day by day and complete the belonging information. In case of not-appropriate statements please check the No-box.

- Ketone bodies:
  Measure the ketone bodies daily in the first morning urine and indicate the result in the respective field. Only one value can be indicated.

- Physical and emotional well-being:
  Evaluate your physical and emotional well-being on a scale reaching from 0 to 10. Please determine one value.

- Symptoms:
  If you don’t have any symptoms please tick no. If symptoms occurred please check the Yes-box and choose one or more symptoms that you have observed.

- Your today’s well-being:
  Evaluate your well-being by choosing one value between 0 and 5 for each statement.

Example: *monday, 18.01.16*

ketone bodies (mg/dl): ☐ 0 ☐ 5 ☐ 15 ☒ 40 ☐ 80 ☐ 160

○ **diet:** ☐ 800 kcal ☐ 1.200 kcal ☐ 1.800 kcal

supplements: ☐ none ☐ fish
☐ others: ____________

○ **digestive rest day:** ☐ rice ☐ potato/vegetables
☐ fruit ☐ others: ____________

☒ **fasting:** honey ☐ no ☒ yes

noon: juice (¼ l) ☐ no ☒ yes
vegetable soup (¼ l) ☒ no ☐ yes

evening: vegetable soup (¼ l) ☐ no ☒ yes

supplements: ☐ none ☐ Kousmine ☐ Kousmine vegan
☒ joghurt ☐ oatmeal soup
☐ rice soup ☐ carott juice
☐ green tea ☐ black tea
☐ others: ____________

○ **day of breaking the fast**

○ **building up day:** ☐ 1 ☐ 2 ☐ 3 ☐ 4

physical well-being:

very bad	0	1	~~2~~ (X)	3	4	5	6	7	8	9	10	excellent

emotional well-being:

very bad	0	1	2	3	4	5	6	7	~~8~~ (X)	9	10	excellent

EXAMPLE

symptoms that occurred in the clinic: ☒ no ☐ yes

☐ headache	☐ muscle pains
☐ sleep disturbances	☐ heartburn
☐ bad breath (ask the nurse)	☐ nausea
	☐ dry mouth
☐ tiredness	☐ cramp in the calf
☐ back pain	☐ diarrhea
☐ sensitivity to cold	☐ restless legs
☐ vertigo	☐ obstipation
☐ hunger	☐ palpitations
☐ skin rash	☐ blurred vision
☐ abdominal bloating	☐ cravings

☐ others: ______________________

EXAMPLE

Today...	At no time	Some of the time	Less than half of the time	More than half of the time	Most of the time	All of the time
...I have felt cheerful and in good spirits	0	1	2	3	☒ 4	5
...I have felt calm and relaxed	0	1	☒ 2	3	4	5
...I have felt active and vigorous	0	1	2	☒ 3	4	5
...I woke up feeling fresh and rested	☒ 0	1	2	3	4	5
...my daily life has been filled with things that interest me	0	1	2	3	☒ 4	5

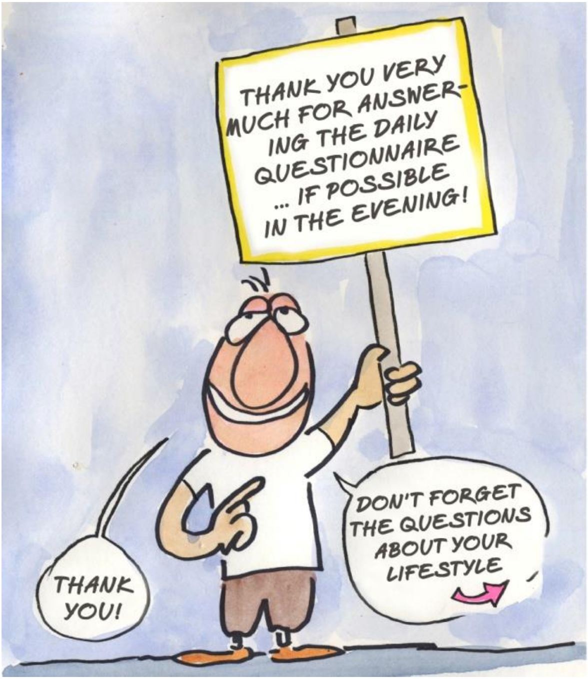
THANK YOU VERY MUCH FOR ANSWER-ING THE DAILY QUESTIONNAIRE ... IF POSSIBLE IN THE EVENING!
DON'T FORGET THE QUESTIONS ABOUT YOUR LIFESTYLE
THANK YOU!

Lifestyle – before the stay

stress/relaxation level:

extreme tension	0	1	2	3	4	5	6	7	8	9	10	total relaxation

nicotine: ☐ none ☐ cigarettes/day: ☐☐
☐ none ☐ cigars/day: ☐☐

alcohol: ☐ none ☐ beer: glasses (0,5 l)/week: ☐☐
☐ none ☐ wine: glasses (0,25 l)/week: ☐☐
☐ none ☐ spirits: glasses (2 cl)/week: ☐☐

coffee: ☐ none ☐ cups/day: ☐☐

media: ☐ none ☐ Smartphone (h/week): ☐☐
☐ none ☐ TV (h/week): ☐☐
☐ none ☐ PC (h/week): ☐☐

exercise: ☐ none ☐ yes: h/week: ☐☐ ,☐

**Over the last two weeks before coming to Buchinger Wilhelmi…**	At no time	Some of the time	Less than half of the time	More than half of the time	Most of the time	All of the time
…I have felt cheerful and in good spirits	0	1	2	3	4	5
…I have felt calm and relaxed	0	1	2	3	4	5
…I have felt active and vigorous	0	1	2	3	4	5
…I woke up feeling fresh and rested	0	1	2	3	4	5
…my daily life has been filled with things that interest me	0	1	2	3	4	5

Day 1 (arrival): ______________________________

ketone bodies (mg/dl): ☐ 0 ☐ 5 ☐ 15 ☐ 40 ☐ 80 ☐ 160

○ **diet:** ☐ 800 kcal ☐ 1.200 kcal ☐ 1.800 kcal

supplements: ☐ none ☐ fish
☐ others: ______________

○ **digestive rest day**: ☐ rice ☐ potato/vegetables
☐ fruit ☐ others: ______

○ **fasting:** honey ☐ no ☐ yes

noon: juice (¼ l) ☐ no ☐ yes
vegetable soup (¼ l) ☐ no ☐ yes
evening: vegetable soup (¼ l) ☐ no ☐ yes

supplements: ☐ none ☐ Kousmine ☐ Kousmine vegan
☐ joghurt ☐ oatmeal soup
☐ rice soup ☐ carott juice
☐ green tea ☐ black tea
☐ others: ______________

○ **day of breaking the fast**

○ **building up day:** ☐ 1 ☐ 2 ☐ 3 ☐ 4

physical well-being:

very bad

0	1	2	3	4	5	6	7	8	9	10

excellent

emotional well-being:

very bad

0	1	2	3	4	5	6	7	8	9	10

excellent

symptoms that occurred in the clinic: ☐ no ☐ yes

- ☐ headache
- ☐ sleep disturbances
- ☐ bad breath
  (ask the nurse)
- ☐ tiredness
- ☐ back pain
- ☐ sensitivity to cold
- ☐ vertigo
- ☐ hunger
- ☐ skin rash
- ☐ abdominal bloating
- ☐ muscle pains
- ☐ heartburn
- ☐ nausea
- ☐ dry mouth
- ☐ cramp in the calf
- ☐ diarrhea
- ☐ restless legs
- ☐ obstipation
- ☐ palpitations
- ☐ blurred vision
- ☐ cravings
- ☐ others: ______________________

**Today…**	At no time	Some of the time	Less than half of the time	More than half of the time	Most of the time	All of the time
…I have felt cheerful and in good spirits	0	1	2	3	4	5
…I have felt calm and relaxed	0	1	2	3	4	5
…I have felt active and vigorous	0	1	2	3	4	5
…I woke up feeling fresh and rested	0	1	2	3	4	5
…my daily life has been filled with things that interest me	0	1	2	3	4	5

Day 2: ______________________

ketone bodies (mg/dl): ☐ 0 ☐ 5 ☐ 15 ☐ 40 ☐ 80 ☐ 160

○ **diet:** ☐ 800 kcal ☐ 1.200 kcal ☐ 1.800 kcal

supplements: ☐ none ☐ fish
☐ others: ______________

○ **digestive rest day**: ☐ rice ☐ potato/vegetables
☐ fruit ☐ others: ________

○ **fasting:** honey ☐ no ☐ yes

noon: juice (¼ l) ☐ no ☐ yes
vegetable soup (¼ l) ☐ no ☐ yes
evening: vegetable soup (¼ l) ☐ no ☐ yes

supplements: ☐ none ☐ Kousmine ☐ Kousmine vegan
☐ joghurt ☐ oatmeal soup
☐ rice soup ☐ carott juice
☐ green tea ☐ black tea
☐ others: ______________

○ **day of breaking the fast**

○ **building up day:** ☐ 1 ☐ 2 ☐ 3 ☐ 4

physical well-being:

very bad	0	1	2	3	4	5	6	7	8	9	10	excellent

emotional well-being:

very bad	0	1	2	3	4	5	6	7	8	9	10	excellent

symptoms that occurred in the clinic: ☐ no ☐ yes

☐ headache
☐ sleep disturbances
☐ bad breath
(ask the nurse)
☐ tiredness
☐ back pain
☐ sensitivity to cold
☐ vertigo
☐ hunger
☐ skin rash
☐ abdominal bloating
☐ muscle pains
☐ heartburn
☐ nausea
☐ dry mouth
☐ cramp in the calf
☐ diarrhea
☐ restless legs
☐ obstipation
☐ palpitations
☐ blurred vision
☐ cravings
☐ others: ____________________

**Today…**	At no time	Some of the time	Less than half of the time	More than half of the time	Most of the time	All of the time
…I have felt cheerful and in good spirits	0	1	2	3	4	5
…I have felt calm and relaxed	0	1	2	3	4	5
…I have felt active and vigorous	0	1	2	3	4	5
…I woke up feeling fresh and rested	0	1	2	3	4	5
…my daily life has been filled with things that interest me	0	1	2	3	4	5

Day 3: ____________________

ketone bodies (mg/dl): ☐ 0 ☐ 5 ☐ 15 ☐ 40 ☐ 80 ☐ 160

○ **diet:** ☐ 800 kcal ☐ 1.200 kcal ☐ 1.800 kcal

supplements: ☐ none ☐ fish
☐ others: ____________

○ **digestive rest day**: ☐ rice ☐ potato/vegetables
☐ fruit ☐ others: ________

○ **fasting:**

	honey	☐ no	☐ yes
noon:	juice (¼ l)	☐ no	☐ yes
	vegetable soup (¼ l)	☐ no	☐ yes
evening:	vegetable soup (¼ l)	☐ no	☐ yes

supplements: ☐ none ☐ Kousmine ☐ Kousmine vegan
☐ joghurt ☐ oatmeal soup
☐ rice soup ☐ carott juice
☐ green tea ☐ black tea
☐ others: ____________

○ **day of breaking the fast**

○ **building up day:** ☐ 1 ☐ 2 ☐ 3 ☐ 4

physical well-being:

very bad	0	1	2	3	4	5	6	7	8	9	10	excellent

emotional well-being:

very bad	0	1	2	3	4	5	6	7	8	9	10	excellent

symptoms that occurred in the clinic: ☐ no ☐ yes

- ☐ headache
- ☐ sleep disturbances
- ☐ bad breath (ask the nurse)
- ☐ tiredness
- ☐ back pain
- ☐ sensitivity to cold
- ☐ vertigo
- ☐ hunger
- ☐ skin rash
- ☐ abdominal bloating
- ☐ muscle pains
- ☐ heartburn
- ☐ nausea
- ☐ dry mouth
- ☐ cramp in the calf
- ☐ diarrhea
- ☐ restless legs
- ☐ obstipation
- ☐ palpitations
- ☐ blurred vision
- ☐ cravings
- ☐ others: ______________________

**Today…**	At no time	Some of the time	Less than half of the time	More than half of the time	Most of the time	All of the time
…I have felt cheerful and in good spirits	0	1	2	3	4	5
…I have felt calm and relaxed	0	1	2	3	4	5
…I have felt active and vigorous	0	1	2	3	4	5
…I woke up feeling fresh and rested	0	1	2	3	4	5
…my daily life has been filled with things that interest me	0	1	2	3	4	5

Day 4: ____________________

ketone bodies (mg/dl): ☐ 0 ☐ 5 ☐ 15 ☐ 40 ☐ 80 ☐ 160

○ **diet:** ☐ 800 kcal ☐ 1.200 kcal ☐ 1.800 kcal

supplements: ☐ none ☐ fish
☐ others: ____________

○ **digestive rest day**: ☐ rice ☐ potato/vegetables
☐ fruit ☐ others: ________

○ **fasting:**

	honey	☐ no	☐ yes
noon:	juice (¼ l)	☐ no	☐ yes
	vegetable soup (¼ l)	☐ no	☐ yes
evening:	vegetable soup (¼ l)	☐ no	☐ yes

supplements: ☐ none ☐ Kousmine ☐ Kousmine vegan
☐ joghurt ☐ oatmeal soup
☐ rice soup ☐ carott juice
☐ green tea ☐ black tea
☐ others: ____________

○ **day of breaking the fast**

○ **building up day:** ☐ 1 ☐ 2 ☐ 3 ☐ 4

physical well-being:

very bad	0	1	2	3	4	5	6	7	8	9	10	excellent

emotional well-being:

very bad	0	1	2	3	4	5	6	7	8	9	10	excellent

symptoms that occurred in the clinic: ☐ no ☐ yes

☐ headache
☐ sleep disturbances
☐ bad breath
(ask the nurse)
☐ tiredness
☐ back pain
☐ sensitivity to cold
☐ vertigo
☐ hunger
☐ skin rash
☐ abdominal bloating
☐ muscle pains
☐ heartburn
☐ nausea
☐ dry mouth
☐ cramp in the calf
☐ diarrhea
☐ restless legs
☐ obstipation
☐ palpitations
☐ blurred vision
☐ cravings
☐ others: ______________________

**Today...**	At no time	Some of the time	Less than half of the time	More than half of the time	Most of the time	All of the time
... I have felt cheerful and in good spirits	0	1	2	3	4	5
... I have felt calm and relaxed	0	1	2	3	4	5
... I have felt active and vigorous	0	1	2	3	4	5
... I woke up feeling fresh and rested	0	1	2	3	4	5
... my daily life has been filled with things that interest me	0	1	2	3	4	5

Day 5: ____________________

ketone bodies (mg/dl): ☐ 0 ☐ 5 ☐ 15 ☐ 40 ☐ 80 ☐ 160

- **diet:** ☐ 800 kcal ☐ 1.200 kcal ☐ 1.800 kcal

  supplements: ☐ none ☐ fish
  ☐ others: ____________________

- **digestive rest day**: ☐ rice ☐ potato/vegetables
  ☐ fruit ☐ others: __________

- **fasting:** honey ☐ no ☐ yes

  noon: juice (¼ l) ☐ no ☐ yes
  vegetable soup (¼ l) ☐ no ☐ yes
  evening: vegetable soup (¼ l) ☐ no ☐ yes

  supplements: ☐ none ☐ Kousmine ☐ Kousmine vegan
  ☐ joghurt ☐ oatmeal soup
  ☐ rice soup ☐ carott juice
  ☐ green tea ☐ black tea
  ☐ others: ____________________

- **day of breaking the fast**

- **building up day:** ☐ 1 ☐ 2 ☐ 3 ☐ 4

physical well-being:

very bad	0	1	2	3	4	5	6	7	8	9	10	excellent

emotional well-being:

very bad	0	1	2	3	4	5	6	7	8	9	10	excellent

symptoms that occurred in the clinic: ☐ no ☐ yes

- ☐ headache
- ☐ sleep disturbances
- ☐ bad breath (ask the nurse)
- ☐ tiredness
- ☐ back pain
- ☐ sensitivity to cold
- ☐ vertigo
- ☐ hunger
- ☐ skin rash
- ☐ abdominal bloating
- ☐ muscle pains
- ☐ heartburn
- ☐ nausea
- ☐ dry mouth
- ☐ cramp in the calf
- ☐ diarrhea
- ☐ restless legs
- ☐ obstipation
- ☐ palpitations
- ☐ blurred vision
- ☐ cravings
- ☐ others: ____________________

**Today...**	At no time	Some of the time	Less than half of the time	More than half of the time	Most of the time	All of the time
...I have felt cheerful and in good spirits	0	1	2	3	4	5
...I have felt calm and relaxed	0	1	2	3	4	5
...I have felt active and vigorous	0	1	2	3	4	5
...I woke up feeling fresh and rested	0	1	2	3	4	5
...my daily life has been filled with things that interest me	0	1	2	3	4	5

Day 6: ______________________

ketone bodies (mg/dl): ☐ 0 ☐ 5 ☐ 15 ☐ 40 ☐ 80 ☐ 160

○ **diet:** ☐ 800 kcal ☐ 1.200 kcal ☐ 1.800 kcal

supplements: ☐ none ☐ fish
☐ others: ______________

○ **digestive rest day**: ☐ rice ☐ potato/vegetables
☐ fruit ☐ others: ________

○ **fasting:** honey ☐ no ☐ yes

noon: juice (¼ l) ☐ no ☐ yes
vegetable soup (¼ l) ☐ no ☐ yes
evening: vegetable soup (¼ l) ☐ no ☐ yes

supplements: ☐ none ☐ Kousmine ☐ Kousmine vegan
☐ joghurt ☐ oatmeal soup
☐ rice soup ☐ carott juice
☐ green tea ☐ black tea
☐ others: ______________

○ **day of breaking the fast**

○ **building up day:** ☐ 1 ☐ 2 ☐ 3 ☐ 4

physical well-being:

very bad	0	1	2	3	4	5	6	7	8	9	10	excellent

emotional well-being:

very bad	0	1	2	3	4	5	6	7	8	9	10	excellent

symptoms that occurred in the clinic: ☐ no ☐ yes

☐ headache
☐ sleep disturbances
☐ bad breath
(ask the nurse)
☐ tiredness
☐ back pain
☐ sensitivity to cold
☐ vertigo
☐ hunger
☐ skin rash
☐ abdominal bloating
☐ muscle pains
☐ heartburn
☐ nausea
☐ dry mouth
☐ cramp in the calf
☐ diarrhea
☐ restless legs
☐ obstipation
☐ palpitations
☐ blurred vision
☐ cravings
☐ others: ______

**Today…**	At no time	Some of the time	Less than half of the time	More than half of the time	Most of the time	All of the time
…I have felt cheerful and in good spirits	0	1	2	3	4	5
…I have felt calm and relaxed	0	1	2	3	4	5
…I have felt active and vigorous	0	1	2	3	4	5
…I woke up feeling fresh and rested	0	1	2	3	4	5
…my daily life has been filled with things that interest me	0	1	2	3	4	5

Day 7: ____________________

ketone bodies (mg/dl): ☐ 0 ☐ 5 ☐ 15 ☐ 40 ☐ 80 ☐ 160

○ **diet:** ☐ 800 kcal ☐ 1.200 kcal ☐ 1.800 kcal

supplements: ☐ none ☐ fish
☐ others: ____________

○ **digestive rest day**: ☐ rice ☐ potato/vegetables
☐ fruit ☐ others: ______

○ **fasting:** honey ☐ no ☐ yes

noon: juice (¼ l) ☐ no ☐ yes
vegetable soup (¼ l) ☐ no ☐ yes
evening: vegetable soup (¼ l) ☐ no ☐ yes

supplements: ☐ none ☐ Kousmine ☐ Kousmine vegan
☐ joghurt ☐ oatmeal soup
☐ rice soup ☐ carott juice
☐ green tea ☐ black tea
☐ others: ____________

○ **day of breaking the fast**

○ **building up day:** ☐ 1 ☐ 2 ☐ 3 ☐ 4

physical well-being:

very bad	0	1	2	3	4	5	6	7	8	9	10	excellent

emotional well-being:

very bad	0	1	2	3	4	5	6	7	8	9	10	excellent

symptoms that occurred in the clinic: ☐ no ☐ yes

☐ headache
☐ sleep disturbances
☐ bad breath
(ask the nurse)
☐ tiredness
☐ back pain
☐ sensitivity to cold
☐ vertigo
☐ hunger
☐ skin rash
☐ abdominal bloating
☐ others: ______________________
☐ muscle pains
☐ heartburn
☐ nausea
☐ dry mouth
☐ cramp in the calf
☐ diarrhea
☐ restless legs
☐ obstipation
☐ palpitations
☐ blurred vision
☐ cravings

**Today...**	At no time	Some of the time	Less than half of the time	More than half of the time	Most of the time	All of the time
...I have felt cheerful and in good spirits	0	1	2	3	4	5
...I have felt calm and relaxed	0	1	2	3	4	5
...I have felt active and vigorous	0	1	2	3	4	5
...I woke up feeling fresh and rested	0	1	2	3	4	5
...my daily life has been filled with things that interest me	0	1	2	3	4	5

Day 8: ____________________

ketone bodies (mg/dl): ☐ 0 ☐ 5 ☐ 15 ☐ 40 ☐ 80 ☐ 160

- **diet:** ☐ 800 kcal ☐ 1.200 kcal ☐ 1.800 kcal

  supplements: ☐ none ☐ fish
  ☐ others: ____________________

- **digestive rest day**: ☐ rice ☐ potato/vegetables
  ☐ fruit ☐ others: __________

- **fasting:** honey ☐ no ☐ yes

  noon: juice (¼ l) ☐ no ☐ yes
  vegetable soup (¼ l) ☐ no ☐ yes
  evening: vegetable soup (¼ l) ☐ no ☐ yes

  supplements: ☐ none ☐ Kousmine ☐ Kousmine vegan
  ☐ joghurt ☐ oatmeal soup
  ☐ rice soup ☐ carott juice
  ☐ green tea ☐ black tea
  ☐ others: ____________________

- **day of breaking the fast**

- **building up day:** ☐ 1 ☐ 2 ☐ 3 ☐ 4

physical well-being:

very bad	0	1	2	3	4	5	6	7	8	9	10	excellent

emotional well-being:

very bad	0	1	2	3	4	5	6	7	8	9	10	excellent

symptoms that occurred in the clinic: ☐ no ☐ yes

- ☐ headache
- ☐ sleep disturbances
- ☐ bad breath (ask the nurse)
- ☐ tiredness
- ☐ back pain
- ☐ sensitivity to cold
- ☐ vertigo
- ☐ hunger
- ☐ skin rash
- ☐ abdominal bloating
- ☐ muscle pains
- ☐ heartburn
- ☐ nausea
- ☐ dry mouth
- ☐ cramp in the calf
- ☐ diarrhea
- ☐ restless legs
- ☐ obstipation
- ☐ palpitations
- ☐ blurred vision
- ☐ cravings
- ☐ others: ______________________

**Today...**	At no time	Some of the time	Less than half of the time	More than half of the time	Most of the time	All of the time
...I have felt cheerful and in good spirits	0	1	2	3	4	5
...I have felt calm and relaxed	0	1	2	3	4	5
...I have felt active and vigorous	0	1	2	3	4	5
...I woke up feeling fresh and rested	0	1	2	3	4	5
...my daily life has been filled with things that interest me	0	1	2	3	4	5

Day 9: ______

ketone bodies (mg/dl): ☐ 0 ☐ 5 ☐ 15 ☐ 40 ☐ 80 ☐ 160

- **diet:** ☐ 800 kcal ☐ 1.200 kcal ☐ 1.800 kcal

  supplements: ☐ none ☐ fish
  ☐ others: ______

- **digestive rest day**: ☐ rice ☐ potato/vegetables
  ☐ fruit ☐ others: ______

- **fasting:** honey ☐ no ☐ yes

  noon: juice (¼ l) ☐ no ☐ yes
  vegetable soup (¼ l) ☐ no ☐ yes
  evening: vegetable soup (¼ l) ☐ no ☐ yes

  supplements: ☐ none ☐ Kousmine ☐ Kousmine vegan
  ☐ joghurt ☐ oatmeal soup
  ☐ rice soup ☐ carott juice
  ☐ green tea ☐ black tea
  ☐ others: ______

- **day of breaking the fast**

- **building up day:** ☐ 1 ☐ 2 ☐ 3 ☐ 4

physical well-being:

very bad	0	1	2	3	4	5	6	7	8	9	10	excellent

emotional well-being:

very bad	0	1	2	3	4	5	6	7	8	9	10	excellent

symptoms that occurred in the clinic: ☐ no ☐ yes

☐ headache
☐ sleep disturbances
☐ bad breath
(ask the nurse)
☐ tiredness
☐ back pain
☐ sensitivity to cold
☐ vertigo
☐ hunger
☐ skin rash
☐ abdominal bloating
☐ others: ______________________

☐ muscle pains
☐ heartburn
☐ nausea
☐ dry mouth
☐ cramp in the calf
☐ diarrhea
☐ restless legs
☐ obstipation
☐ palpitations
☐ blurred vision
☐ cravings

**Today...**	At no time	Some of the time	Less than half of the time	More than half of the time	Most of the time	All of the time
…I have felt cheerful and in good spirits	0	1	2	3	4	5
…I have felt calm and relaxed	0	1	2	3	4	5
…I have felt active and vigorous	0	1	2	3	4	5
…I woke up feeling fresh and rested	0	1	2	3	4	5
…my daily life has been filled with things that interest me	0	1	2	3	4	5

Day 10: ______________________________

ketone bodies (mg/dl): ☐ 0 ☐ 5 ☐ 15 ☐ 40 ☐ 80 ☐ 160

○ **diet:** ☐ 800 kcal ☐ 1.200 kcal ☐ 1.800 kcal

supplements: ☐ none ☐ fish
☐ others: ______________

○ **digestive rest day**: ☐ rice ☐ potato/vegetables
☐ fruit ☐ others: ________

○ **fasting:** honey ☐ no ☐ yes

noon: juice (¼ l) ☐ no ☐ yes
vegetable soup (¼ l) ☐ no ☐ yes
evening: vegetable soup (¼ l) ☐ no ☐ yes

supplements: ☐ none ☐ Kousmine ☐ Kousmine vegan
☐ joghurt ☐ oatmeal soup
☐ rice soup ☐ carott juice
☐ green tea ☐ black tea
☐ others: ______________

○ **day of breaking the fast**

○ **building up day:** ☐ 1 ☐ 2 ☐ 3 ☐ 4

physical well-being:

very bad	0	1	2	3	4	5	6	7	8	9	10	excellent

emotional well-being:

very bad	0	1	2	3	4	5	6	7	8	9	10	excellent

symptoms that occurred in the clinic: ☐ no ☐ yes

☐ headache
☐ sleep disturbances
☐ bad breath
(ask the nurse)
☐ tiredness
☐ back pain
☐ sensitivity to cold
☐ vertigo
☐ hunger
☐ skin rash
☐ abdominal bloating
☐ muscle pains
☐ heartburn
☐ nausea
☐ dry mouth
☐ cramp in the calf
☐ diarrhea
☐ restless legs
☐ obstipation
☐ palpitations
☐ blurred vision
☐ cravings
☐ others: ____________________

**Today...**	At no time	Some of the time	Less than half of the time	More than half of the time	Most of the time	All of the time
...I have felt cheerful and in good spirits	0	1	2	3	4	5
...I have felt calm and relaxed	0	1	2	3	4	5
...I have felt active and vigorous	0	1	2	3	4	5
...I woke up feeling fresh and rested	0	1	2	3	4	5
...my daily life has been filled with things that interest me	0	1	2	3	4	5

Day 11: ______________________________

ketone bodies (mg/dl): ☐ 0 ☐ 5 ☐ 15 ☐ 40 ☐ 80 ☐ 160

- **diet:** ☐ 800 kcal ☐ 1.200 kcal ☐ 1.800 kcal

  supplements: ☐ none ☐ fish
  ☐ others: ______________

- **digestive rest day:** ☐ rice ☐ potato/vegetables
  ☐ fruit ☐ others: ______

- **fasting:** honey ☐ no ☐ yes

  noon: juice (¼ l) ☐ no ☐ yes
  vegetable soup (¼ l) ☐ no ☐ yes
  evening: vegetable soup (¼ l) ☐ no ☐ yes

  supplements: ☐ none ☐ Kousmine ☐ Kousmine vegan
  ☐ joghurt ☐ oatmeal soup
  ☐ rice soup ☐ carott juice
  ☐ green tea ☐ black tea
  ☐ others: ______________

- **day of breaking the fast**

- **building up day:** ☐ 1 ☐ 2 ☐ 3 ☐ 4

physical well-being:

very bad	0	1	2	3	4	5	6	7	8	9	10	excellent

emotional well-being:

very bad	0	1	2	3	4	5	6	7	8	9	10	excellent

symptoms that occurred in the clinic: ☐ no ☐ yes

- ☐ headache
- ☐ sleep disturbances
- ☐ bad breath (ask the nurse)
- ☐ tiredness
- ☐ back pain
- ☐ sensitivity to cold
- ☐ vertigo
- ☐ hunger
- ☐ skin rash
- ☐ abdominal bloating
- ☐ muscle pains
- ☐ heartburn
- ☐ nausea
- ☐ dry mouth
- ☐ cramp in the calf
- ☐ diarrhea
- ☐ restless legs
- ☐ obstipation
- ☐ palpitations
- ☐ blurred vision
- ☐ cravings
- ☐ others: ______________________________

**Today...**	At no time	Some of the time	Less than half of the time	More than half of the time	Most of the time	All of the time
...I have felt cheerful and in good spirits	0	1	2	3	4	5
...I have felt calm and relaxed	0	1	2	3	4	5
...I have felt active and vigorous	0	1	2	3	4	5
...I woke up feeling fresh and rested	0	1	2	3	4	5
...my daily life has been filled with things that interest me	0	1	2	3	4	5

Day 12: ____________________

ketone bodies (mg/dl): ☐ 0 ☐ 5 ☐ 15 ☐ 40 ☐ 80 ☐ 160

○ **diet:** ☐ 800 kcal ☐ 1.200 kcal ☐ 1.800 kcal

supplements: ☐ none ☐ fish
☐ others: ____________

○ **digestive rest day**: ☐ rice ☐ potato/vegetables
☐ fruit ☐ others: ________

○ **fasting:** honey ☐ no ☐ yes

noon: juice (¼ l) ☐ no ☐ yes
vegetable soup (¼ l) ☐ no ☐ yes
evening: vegetable soup (¼ l) ☐ no ☐ yes

supplements: ☐ none ☐ Kousmine ☐ Kousmine vegan
☐ joghurt ☐ oatmeal soup
☐ rice soup ☐ carott juice
☐ green tea ☐ black tea
☐ others: ____________

○ **day of breaking the fast**

○ **building up day:** ☐ 1 ☐ 2 ☐ 3 ☐ 4

physical well-being:

very bad	0	1	2	3	4	5	6	7	8	9	10	excellent

emotional well-being:

very bad	0	1	2	3	4	5	6	7	8	9	10	excellent

symptoms that occurred in the clinic: ☐ no ☐ yes

- ☐ headache
- ☐ sleep disturbances
- ☐ bad breath (ask the nurse)
- ☐ tiredness
- ☐ back pain
- ☐ sensitivity to cold
- ☐ vertigo
- ☐ hunger
- ☐ skin rash
- ☐ abdominal bloating
- ☐ muscle pains
- ☐ heartburn
- ☐ nausea
- ☐ dry mouth
- ☐ cramp in the calf
- ☐ diarrhea
- ☐ restless legs
- ☐ obstipation
- ☐ palpitations
- ☐ blurred vision
- ☐ cravings
- ☐ others: ______________________

**Today...**	At no time	Some of the time	Less than half of the time	More than half of the time	Most of the time	All of the time
...I have felt cheerful and in good spirits	0	1	2	3	4	5
...I have felt calm and relaxed	0	1	2	3	4	5
...I have felt active and vigorous	0	1	2	3	4	5
...I woke up feeling fresh and rested	0	1	2	3	4	5
...my daily life has been filled with things that interest me	0	1	2	3	4	5

Day 13: ____________________

ketone bodies (mg/dl): ☐ 0 ☐ 5 ☐ 15 ☐ 40 ☐ 80 ☐ 160

○ **diet:** ☐ 800 kcal ☐ 1.200 kcal ☐ 1.800 kcal

supplements: ☐ none ☐ fish
☐ others: ____________

○ **digestive rest day**: ☐ rice ☐ potato/vegetables
☐ fruit ☐ others: ________

○ **fasting:** honey ☐ no ☐ yes

noon:	juice (¼ l)	☐ no	☐ yes
	vegetable soup (¼ l)	☐ no	☐ yes
evening:	vegetable soup (¼ l)	☐ no	☐ yes

supplements: ☐ none ☐ Kousmine ☐ Kousmine vegan
☐ joghurt ☐ oatmeal soup
☐ rice soup ☐ carott juice
☐ green tea ☐ black tea
☐ others: ____________

○ **day of breaking the fast**

○ **building up day:** ☐ 1 ☐ 2 ☐ 3 ☐ 4

physical well-being:

very bad	0	1	2	3	4	5	6	7	8	9	10	excellent

emotional well-being:

very bad	0	1	2	3	4	5	6	7	8	9	10	excellent

symptoms that occurred in the clinic: ☐ no ☐ yes

- ☐ headache
- ☐ sleep disturbances
- ☐ bad breath (ask the nurse)
- ☐ tiredness
- ☐ back pain
- ☐ sensitivity to cold
- ☐ vertigo
- ☐ hunger
- ☐ skin rash
- ☐ abdominal bloating
- ☐ muscle pains
- ☐ heartburn
- ☐ nausea
- ☐ dry mouth
- ☐ cramp in the calf
- ☐ diarrhea
- ☐ restless legs
- ☐ obstipation
- ☐ palpitations
- ☐ blurred vision
- ☐ cravings
- ☐ others: ______________________

**Today...**	At no time	Some of the time	Less than half of the time	More than half of the time	Most of the time	All of the time
...I have felt cheerful and in good spirits	0	1	2	3	4	5
...I have felt calm and relaxed	0	1	2	3	4	5
...I have felt active and vigorous	0	1	2	3	4	5
...I woke up feeling fresh and rested	0	1	2	3	4	5
...my daily life has been filled with things that interest me	0	1	2	3	4	5

Day 14: ______

ketone bodies (mg/dl): ☐ 0 ☐ 5 ☐ 15 ☐ 40 ☐ 80 ☐ 160

○ **diet:** ☐ 800 kcal ☐ 1.200 kcal ☐ 1.800 kcal

supplements: ☐ none ☐ fish
☐ others: ______

○ **digestive rest day**: ☐ rice ☐ potato/vegetables
☐ fruit ☐ others: ______

○ **fasting:** honey ☐ no ☐ yes

noon: juice (¼ l) ☐ no ☐ yes
vegetable soup (¼ l) ☐ no ☐ yes
evening: vegetable soup (¼ l) ☐ no ☐ yes

supplements: ☐ none ☐ Kousmine ☐ Kousmine vegan
☐ joghurt ☐ oatmeal soup
☐ rice soup ☐ carott juice
☐ green tea ☐ black tea
☐ others: ______

○ **day of breaking the fast**

○ **building up day:** ☐ 1 ☐ 2 ☐ 3 ☐ 4

physical well-being:

very bad	0	1	2	3	4	5	6	7	8	9	10	excellent

emotional well-being:

very bad	0	1	2	3	4	5	6	7	8	9	10	excellent

symptoms that occurred in the clinic: ☐ no ☐ yes

- ☐ headache
- ☐ sleep disturbances
- ☐ bad breath (ask the nurse)
- ☐ tiredness
- ☐ back pain
- ☐ sensitivity to cold
- ☐ vertigo
- ☐ hunger
- ☐ skin rash
- ☐ abdominal bloating
- ☐ muscle pains
- ☐ heartburn
- ☐ nausea
- ☐ dry mouth
- ☐ cramp in the calf
- ☐ diarrhea
- ☐ restless legs
- ☐ obstipation
- ☐ palpitations
- ☐ blurred vision
- ☐ cravings
- ☐ others: ______________________

**Today...**	At no time	Some of the time	Less than half of the time	More than half of the time	Most of the time	All of the time
...I have felt cheerful and in good spirits	0	1	2	3	4	5
...I have felt calm and relaxed	0	1	2	3	4	5
...I have felt active and vigorous	0	1	2	3	4	5
...I woke up feeling fresh and rested	0	1	2	3	4	5
...my daily life has been filled with things that interest me	0	1	2	3	4	5

Day 15: ______________________

ketone bodies (mg/dl): ☐ 0 ☐ 5 ☐ 15 ☐ 40 ☐ 80 ☐ 160

○ **diet:** ☐ 800 kcal ☐ 1.200 kcal ☐ 1.800 kcal

supplements: ☐ none ☐ fish
☐ others: ______________

○ **digestive rest day**: ☐ rice ☐ potato/vegetables
☐ fruit ☐ others: ________

○ **fasting:** honey ☐ no ☐ yes

noon: juice (¼ l) ☐ no ☐ yes
vegetable soup (¼ l) ☐ no ☐ yes
evening: vegetable soup (¼ l) ☐ no ☐ yes

supplements: ☐ none ☐ Kousmine ☐ Kousmine vegan
☐ joghurt ☐ oatmeal soup
☐ rice soup ☐ carott juice
☐ green tea ☐ black tea
☐ others: ______________

○ **day of breaking the fast**

○ **building up day:** ☐ 1 ☐ 2 ☐ 3 ☐ 4

physical well-being:

very bad | 0 | 1 | 2 | 3 | 4 | 5 | 6 | 7 | 8 | 9 | 10 | excellent

emotional well-being:

very bad | 0 | 1 | 2 | 3 | 4 | 5 | 6 | 7 | 8 | 9 | 10 | excellent

symptoms that occurred in the clinic: ☐ no ☐ yes

- ☐ headache
- ☐ sleep disturbances
- ☐ bad breath (ask the nurse)
- ☐ tiredness
- ☐ back pain
- ☐ sensitivity to cold
- ☐ vertigo
- ☐ hunger
- ☐ skin rash
- ☐ abdominal bloating
- ☐ muscle pains
- ☐ heartburn
- ☐ nausea
- ☐ dry mouth
- ☐ cramp in the calf
- ☐ diarrhea
- ☐ restless legs
- ☐ obstipation
- ☐ palpitations
- ☐ blurred vision
- ☐ cravings
- ☐ others: ______________________

**Today...**	At no time	Some of the time	Less than half of the time	More than half of the time	Most of the time	All of the time
...I have felt cheerful and in good spirits	0	1	2	3	4	5
...I have felt calm and relaxed	0	1	2	3	4	5
...I have felt active and vigorous	0	1	2	3	4	5
...I woke up feeling fresh and rested	0	1	2	3	4	5
...my daily life has been filled with things that interest me	0	1	2	3	4	5

Day 16: ______________________________

ketone bodies (mg/dl): ☐ 0 ☐ 5 ☐ 15 ☐ 40 ☐ 80 ☐ 160

○ **diet:** ☐ 800 kcal ☐ 1.200 kcal ☐ 1.800 kcal

supplements: ☐ none ☐ fish
☐ others: ______________

○ **digestive rest day**: ☐ rice ☐ potato/vegetables
☐ fruit ☐ others: ________

○ **fasting:** honey ☐ no ☐ yes

noon: juice (¼ l) ☐ no ☐ yes
vegetable soup (¼ l) ☐ no ☐ yes
evening: vegetable soup (¼ l) ☐ no ☐ yes

supplements: ☐ none ☐ Kousmine ☐ Kousmine vegan
☐ joghurt ☐ oatmeal soup
☐ rice soup ☐ carott juice
☐ green tea ☐ black tea
☐ others: ______________

○ **day of breaking the fast**

○ **building up day:** ☐ 1 ☐ 2 ☐ 3 ☐ 4

physical well-being:

very bad	0	1	2	3	4	5	6	7	8	9	10	excellent

emotional well-being:

very bad	0	1	2	3	4	5	6	7	8	9	10	excellent

symptoms that occurred in the clinic: ☐ no ☐ yes

- ☐ headache
- ☐ sleep disturbances
- ☐ bad breath (ask the nurse)
- ☐ tiredness
- ☐ back pain
- ☐ sensitivity to cold
- ☐ vertigo
- ☐ hunger
- ☐ skin rash
- ☐ abdominal bloating
- ☐ muscle pains
- ☐ heartburn
- ☐ nausea
- ☐ dry mouth
- ☐ cramp in the calf
- ☐ diarrhea
- ☐ restless legs
- ☐ obstipation
- ☐ palpitations
- ☐ blurred vision
- ☐ cravings
- ☐ others: ______________________

**Today...**	At no time	Some of the time	Less than half of the time	More than half of the time	Most of the time	All of the time
...I have felt cheerful and in good spirits	0	1	2	3	4	5
...I have felt calm and relaxed	0	1	2	3	4	5
...I have felt active and vigorous	0	1	2	3	4	5
...I woke up feeling fresh and rested	0	1	2	3	4	5
...my daily life has been filled with things that interest me	0	1	2	3	4	5

Day 17: ______________________________

ketone bodies (mg/dl): ☐ 0 ☐ 5 ☐ 15 ☐ 40 ☐ 80 ☐ 160

- **diet:** ☐ 800 kcal ☐ 1.200 kcal ☐ 1.800 kcal

  supplements: ☐ none ☐ fish
  ☐ others: ____________________

- **digestive rest day**: ☐ rice ☐ potato/vegetables
  ☐ fruit ☐ others: __________

- **fasting:** honey ☐ no ☐ yes

  noon: juice (¼ l) ☐ no ☐ yes
  vegetable soup (¼ l) ☐ no ☐ yes
  evening: vegetable soup (¼ l) ☐ no ☐ yes

  supplements: ☐ none ☐ Kousmine ☐ Kousmine vegan
  ☐ joghurt ☐ oatmeal soup
  ☐ rice soup ☐ carott juice
  ☐ green tea ☐ black tea
  ☐ others: ____________________

- **day of breaking the fast**

- **building up day:** ☐ 1 ☐ 2 ☐ 3 ☐ 4

physical well-being:

very bad | 0 | 1 | 2 | 3 | 4 | 5 | 6 | 7 | 8 | 9 | 10 | excellent

emotional well-being:

very bad | 0 | 1 | 2 | 3 | 4 | 5 | 6 | 7 | 8 | 9 | 10 | excellent

symptoms that occurred in the clinic: ☐ no ☐ yes

- ☐ headache
- ☐ sleep disturbances
- ☐ bad breath (ask the nurse)
- ☐ tiredness
- ☐ back pain
- ☐ sensitivity to cold
- ☐ vertigo
- ☐ hunger
- ☐ skin rash
- ☐ abdominal bloating
- ☐ muscle pains
- ☐ heartburn
- ☐ nausea
- ☐ dry mouth
- ☐ cramp in the calf
- ☐ diarrhea
- ☐ restless legs
- ☐ obstipation
- ☐ palpitations
- ☐ blurred vision
- ☐ cravings
- ☐ others: ____________________

**Today…**	At no time	Some of the time	Less than half of the time	More than half of the time	Most of the time	All of the time
…I have felt cheerful and in good spirits	0	1	2	3	4	5
…I have felt calm and relaxed	0	1	2	3	4	5
…I have felt active and vigorous	0	1	2	3	4	5
…I woke up feeling fresh and rested	0	1	2	3	4	5
…my daily life has been filled with things that interest me	0	1	2	3	4	5

Day 18: ______________________________

ketone bodies (mg/dl): ☐ 0 ☐ 5 ☐ 15 ☐ 40 ☐ 80 ☐ 160

○ **diet:** ☐ 800 kcal ☐ 1.200 kcal ☐ 1.800 kcal

supplements: ☐ none ☐ fish
☐ others: ____________________

○ **digestive rest day**: ☐ rice ☐ potato/vegetables
☐ fruit ☐ others: __________

○ **fasting:** honey ☐ no ☐ yes

noon: juice (¼ l) ☐ no ☐ yes
vegetable soup (¼ l) ☐ no ☐ yes
evening: vegetable soup (¼ l) ☐ no ☐ yes

supplements: ☐ none ☐ Kousmine ☐ Kousmine vegan
☐ joghurt ☐ oatmeal soup
☐ rice soup ☐ carott juice
☐ green tea ☐ black tea
☐ others: ____________________

○ **day of breaking the fast**

○ **building up day:** ☐ 1 ☐ 2 ☐ 3 ☐ 4

physical well-being:

very bad	0	1	2	3	4	5	6	7	8	9	10	excellent

emotional well-being:

very bad	0	1	2	3	4	5	6	7	8	9	10	excellent

symptoms that occurred in the clinic: ☐ no ☐ yes

- ☐ headache
- ☐ sleep disturbances
- ☐ bad breath
  (ask the nurse)
- ☐ tiredness
- ☐ back pain
- ☐ sensitivity to cold
- ☐ vertigo
- ☐ hunger
- ☐ skin rash
- ☐ abdominal bloating
- ☐ muscle pains
- ☐ heartburn
- ☐ nausea
- ☐ dry mouth
- ☐ cramp in the calf
- ☐ diarrhea
- ☐ restless legs
- ☐ obstipation
- ☐ palpitations
- ☐ blurred vision
- ☐ cravings
- ☐ others: ______________________

**Today...**	At no time	Some of the time	Less than half of the time	More than half of the time	Most of the time	All of the time
...I have felt cheerful and in good spirits	0	1	2	3	4	5
...I have felt calm and relaxed	0	1	2	3	4	5
...I have felt active and vigorous	0	1	2	3	4	5
...I woke up feeling fresh and rested	0	1	2	3	4	5
...my daily life has been filled with things that interest me	0	1	2	3	4	5

Day 19: ______________________________

ketone bodies (mg/dl): ☐ 0 ☐ 5 ☐ 15 ☐ 40 ☐ 80 ☐ 160

○ **diet:** ☐ 800 kcal ☐ 1.200 kcal ☐ 1.800 kcal

supplements: ☐ none ☐ fish
☐ others: ______________

○ **digestive rest day**: ☐ rice ☐ potato/vegetables
☐ fruit ☐ others: ________

○ **fasting:** honey ☐ no ☐ yes

noon: juice (¼ l) ☐ no ☐ yes
vegetable soup (¼ l) ☐ no ☐ yes
evening: vegetable soup (¼ l) ☐ no ☐ yes

supplements: ☐ none ☐ Kousmine ☐ Kousmine vegan
☐ joghurt ☐ oatmeal soup
☐ rice soup ☐ carott juice
☐ green tea ☐ black tea
☐ others: ______________

○ **day of breaking the fast**

○ **building up day:** ☐ 1 ☐ 2 ☐ 3 ☐ 4

physical well-being:

very bad	0	1	2	3	4	5	6	7	8	9	10	excellent

emotional well-being:

very bad	0	1	2	3	4	5	6	7	8	9	10	excellent

symptoms that occurred in the clinic: ☐ no ☐ yes

- ☐ headache
- ☐ sleep disturbances
- ☐ bad breath (ask the nurse)
- ☐ tiredness
- ☐ back pain
- ☐ sensitivity to cold
- ☐ vertigo
- ☐ hunger
- ☐ skin rash
- ☐ abdominal bloating
- ☐ muscle pains
- ☐ heartburn
- ☐ nausea
- ☐ dry mouth
- ☐ cramp in the calf
- ☐ diarrhea
- ☐ restless legs
- ☐ obstipation
- ☐ palpitations
- ☐ blurred vision
- ☐ cravings
- ☐ others: ____________________

Today...	At no time	Some of the time	Less than half of the time	More than half of the time	Most of the time	All of the time
...I have felt cheerful and in good spirits	0	1	2	3	4	5
...I have felt calm and relaxed	0	1	2	3	4	5
...I have felt active and vigorous	0	1	2	3	4	5
...I woke up feeling fresh and rested	0	1	2	3	4	5
...my daily life has been filled with things that interest me	0	1	2	3	4	5

Day 20: ____________________

ketone bodies (mg/dl): ☐ 0 ☐ 5 ☐ 15 ☐ 40 ☐ 80 ☐ 160

○ **diet:** ☐ 800 kcal ☐ 1.200 kcal ☐ 1.800 kcal

supplements: ☐ none ☐ fish
☐ others: ____________

○ **digestive rest day**: ☐ rice ☐ potato/vegetables
☐ fruit ☐ others: ____________

○ **fasting:** honey ☐ no ☐ yes

noon: juice (¼ l) ☐ no ☐ yes
vegetable soup (¼ l) ☐ no ☐ yes
evening: vegetable soup (¼ l) ☐ no ☐ yes

supplements: ☐ none ☐ Kousmine ☐ Kousmine vegan
☐ joghurt ☐ oatmeal soup
☐ rice soup ☐ carott juice
☐ green tea ☐ black tea
☐ others: ____________

○ **day of breaking the fast**

○ **building up day:** ☐ 1 ☐ 2 ☐ 3 ☐ 4

physical well-being:

very bad | 0 | 1 | 2 | 3 | 4 | 5 | 6 | 7 | 8 | 9 | 10 | excellent

emotional well-being:

very bad | 0 | 1 | 2 | 3 | 4 | 5 | 6 | 7 | 8 | 9 | 10 | excellent

symptoms that occurred in the clinic: ☐ no ☐ yes

☐ headache
☐ sleep disturbances
☐ bad breath
(ask the nurse)
☐ tiredness
☐ back pain
☐ sensitivity to cold
☐ vertigo
☐ hunger
☐ skin rash
☐ abdominal bloating
☐ muscle pains
☐ heartburn
☐ nausea
☐ dry mouth
☐ cramp in the calf
☐ diarrhea
☐ restless legs
☐ obstipation
☐ palpitations
☐ blurred vision
☐ cravings
☐ others: ______________________________

**Today…**	At no time	Some of the time	Less than half of the time	More than half of the time	Most of the time	All of the time
…I have felt cheerful and in good spirits	0	1	2	3	4	5
…I have felt calm and relaxed	0	1	2	3	4	5
…I have felt active and vigorous	0	1	2	3	4	5
…I woke up feeling fresh and rested	0	1	2	3	4	5
…my daily life has been filled with things that interest me	0	1	2	3	4	5

Day 21: ____________________

ketone bodies (mg/dl): ☐ 0 ☐ 5 ☐ 15 ☐ 40 ☐ 80 ☐ 160

○ **diet:** ☐ 800 kcal ☐ 1.200 kcal ☐ 1.800 kcal

supplements: ☐ none ☐ fish
☐ others: ____________

○ **digestive rest day**: ☐ rice ☐ potato/vegetables
☐ fruit ☐ others: ________

○ **fasting:**

	honey	☐ no	☐ yes
noon:	juice (¼ l)	☐ no	☐ yes
	vegetable soup (¼ l)	☐ no	☐ yes
evening:	vegetable soup (¼ l)	☐ no	☐ yes

supplements: ☐ none ☐ Kousmine ☐ Kousmine vegan
☐ joghurt ☐ oatmeal soup
☐ rice soup ☐ carott juice
☐ green tea ☐ black tea
☐ others: ____________

○ **day of breaking the fast**

○ **building up day:** ☐ 1 ☐ 2 ☐ 3 ☐ 4

physical well-being:

very bad	0	1	2	3	4	5	6	7	8	9	10	excellent

emotional well-being:

very bad	0	1	2	3	4	5	6	7	8	9	10	excellent

symptoms that occurred in the clinic: ☐ no ☐ yes

- ☐ headache
- ☐ sleep disturbances
- ☐ bad breath (ask the nurse)
- ☐ tiredness
- ☐ back pain
- ☐ sensitivity to cold
- ☐ vertigo
- ☐ hunger
- ☐ skin rash
- ☐ abdominal bloating
- ☐ muscle pains
- ☐ heartburn
- ☐ nausea
- ☐ dry mouth
- ☐ cramp in the calf
- ☐ diarrhea
- ☐ restless legs
- ☐ obstipation
- ☐ palpitations
- ☐ blurred vision
- ☐ cravings
- ☐ others: ______

Today...	At no time	Some of the time	Less than half of the time	More than half of the time	Most of the time	All of the time
...I have felt cheerful and in good spirits	0	1	2	3	4	5
...I have felt calm and relaxed	0	1	2	3	4	5
...I have felt active and vigorous	0	1	2	3	4	5
...I woke up feeling fresh and rested	0	1	2	3	4	5
...my daily life has been filled with things that interest me	0	1	2	3	4	5

Day 22: ____________________

ketone bodies (mg/dl): ☐ 0 ☐ 5 ☐ 15 ☐ 40 ☐ 80 ☐ 160

○ **diet:** ☐ 800 kcal ☐ 1.200 kcal ☐ 1.800 kcal

supplements: ☐ none ☐ fish
☐ others: ____________

○ **digestive rest day**: ☐ rice ☐ potato/vegetables
☐ fruit ☐ others: ________

○ **fasting:** honey ☐ no ☐ yes

noon: juice (¼ l) ☐ no ☐ yes
vegetable soup (¼ l) ☐ no ☐ yes
evening: vegetable soup (¼ l) ☐ no ☐ yes

supplements: ☐ none ☐ Kousmine ☐ Kousmine vegan
☐ joghurt ☐ oatmeal soup
☐ rice soup ☐ carott juice
☐ green tea ☐ black tea
☐ others: ____________

○ **day of breaking the fast**

○ **building up day:** ☐ 1 ☐ 2 ☐ 3 ☐ 4

physical well-being:

very bad	0	1	2	3	4	5	6	7	8	9	10	excellent

emotional well-being:

very bad	0	1	2	3	4	5	6	7	8	9	10	excellent

symptoms that occurred in the clinic: ☐ no ☐ yes

☐ headache
☐ sleep disturbances
☐ bad breath
(ask the nurse)
☐ tiredness
☐ back pain
☐ sensitivity to cold
☐ vertigo
☐ hunger
☐ skin rash
☐ abdominal bloating
☐ muscle pains
☐ heartburn
☐ nausea
☐ dry mouth
☐ cramp in the calf
☐ diarrhea
☐ restless legs
☐ obstipation
☐ palpitations
☐ blurred vision
☐ cravings
☐ others: ______________________

**Today…**	At no time	Some of the time	Less than half of the time	More than half of the time	Most of the time	All of the time
…I have felt cheerful and in good spirits	0	1	2	3	4	5
…I have felt calm and relaxed	0	1	2	3	4	5
…I have felt active and vigorous	0	1	2	3	4	5
…I woke up feeling fresh and rested	0	1	2	3	4	5
…my daily life has been filled with things that interest me	0	1	2	3	4	5

Questions at the end of your stay

1) Did you have a major health problem at the beginning of your stay?
   ☐ no
   ☐ yes, which one? ______________________________

   ____

   How did your major health problem evolve during your stay?

much worse	1	2	3	4	5	6	7	much better

2) During your stay you underwent a diet or a fasting therapy. How would you evaluate it:

it was a nightmare	1	2	3	4	5	6	7	a fantastic experience

3) If you compare your stay with a usual holiday, Buchinger Wilhelmi had:

   ☐ a less relaxing effect   ☐ the same relaxing effect   ☐ a more relaxing effect

4) The length of your stay was:

   ☐ too short   ☐ just right   ☐ too long

5) In case you fasted, would you have wished to extend the length of your fast on the last day of fasting?

   ☐ no   ☐ yes

Lifestyle – during the stay

stress/relaxation level:

extreme tension	0	1	2	3	4	5	6	7	8	9	10	total relaxation

nicotine: ☐ none ☐ cigarettes/day: ☐☐
☐ none ☐ cigars/day: ☐☐

alcohol: ☐ none ☐ beer: glasses (0,5 l)/week: ☐☐
☐ none ☐ wine: glasses (0,25 l)/week: ☐☐
☐ none ☐ spirits: glasses (2 cl)/week: ☐☐

coffee: ☐ none ☐ cups/day: ☐☐

media: ☐ none ☐ Smartphone (h/week): ☐☐
☐ none ☐ TV (h/week): ☐☐
☐ none ☐ PC (h/week): ☐☐

exercise: ☐ none ☐ yes: h/week: ☐☐ , ☐

Over the last weeks…	At no time	Some of the time	Less than half of the time	More than half of the time	Most of the time	All of the time
…I have felt cheerful and in good spirits	0	1	2	3	4	5
…I have felt calm and relaxed	0	1	2	3	4	5
…I have felt active and vigorous	0	1	2	3	4	5
…I woke up feeling fresh and rested	0	1	2	3	4	5
…my daily life has been filled with things that interest me	0	1	2	3	4	5

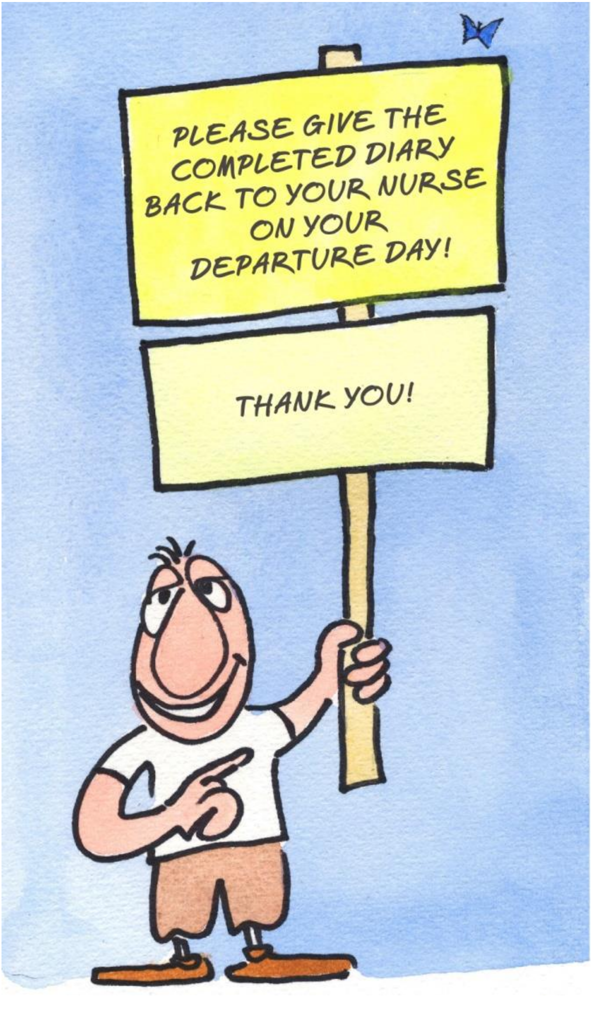
PLEASE GIVE THE COMPLETED DIARY BACK TO YOUR NURSE ON YOUR DEPARTURE DAY!
THANK YOU!

The following questions are not indispensable for our scientific data collection but help Buchinger Wilhelmi to better target your needs:

Was the filling of this diary acceptable for you?

Do you have any suggestions for improvement?

Did your stay at Buchinger Wilhelmi fulfill your expectations?

To which extent will the stay influence your life?

Do you need help from our service “Feel well after Buchinger Wilhelmi”? A nutritionist could assist you at home.
E-Mail: nutrition@buchinger-wilhelmi.com

Thank you very much and see you soon!

We will write an email to you after two weeks, three and eleven months and hope to find you well.

Have a good trip back home!
Follow up Buchinger Wilhelmi
