# Supplementary material for: Safety, health improvement and well-being during a 4 to 21-day fasting period in an observational study including 1422 subjects
Source: PLoS One. 2019 Jan 2;14(1):e0209353. doi: 10.1371/journal.pone.0209353 (PMC6314618; doi:10.1371/journal.pone.0209353)
Supplement: S6 Text — (PDF) [file pone.0209353.s023.pdf]

S6 Text. Questionnaires in French.

Journal

d’évaluation personnelle

Nom : ______________________________

Numéro de votre chambre : ______________

Séjour de : __________ jusqu’à : ____________

Chers hôtes,
chère patiente, cher patient,

Le changement de métabolisme qui se produit lors d'un jeûne thérapeutique ou d'un régime basse-calorie a un impact sur le bien-être. Lorsque vous remplirez le questionnaire quotidien, vous pourrez prendre conscience des changements qui s'opèrent en vous. L'attention portée à votre propre état de bien-être vous sera utile au quotidien.
Pour pouvoir évaluer votre évolution, les données que vous voudrez bien recueillir quotidiennement doivent être complètes.

Pensez aussi à répondre aux questions concernant :

1. votre style de vie avant le séjour (p 7)
2. votre style de vie pendant la durée de votre séjour (p 53)
3. questions à la fin du séjour (p 52)

Un grand merci de remplir tous les questionnaires en détail.

Merci également de retourner le « journal d'évaluation personnelle » à votre infirmière le jour de votre départ.

Merci enfin pour votre aimable participation !

Dr Françoise Wilhelmi de Toledo et
l'équipe scientifique Buchinger Wilhelmi

Instructions :

- Date :
  Nous vous prions de noter la date. Le jour 1 correspond au jour d'arrivée.

- Régime :
  Chaque jour vous documenterez votre forme d'alimentation (régime, repos digestif, jeûne, interruption du jeûne, jour de réalimentation) et leurs annexes (par exemple jeûne avec miel, jus et bouillon). Si vous ne recevez pas de supplément additionnel cochez « non ». Sinon cochez les suppléments correspondants.

- Corps cétoniques :
  Mesurez les corps cétoniques à l'aide d'un stick dans la première urine du matin et documentez le résultat dans la case appropriée. Vous ne pouvez indiquer seule qu'une valeur par jour.

- Votre état de santé physique/émotionnelle :
  Evaluez votre état de santé émotionnelle et physique sur une échelle visuelle allant de 0 à 10 intuitivement. Il s'agit là d'une évaluation subjective.

- Symptômes :
  Si vous n'avez observé aucun symptôme, veuillez cocher la case « non ». Si vous avez observé un ou plusieurs symptômes, cochez « oui » et la/les case(s) correspondante(s).

- Bien-être quotidien :
  Evaluez votre bien-être de 0 à 5 en fonction de fréquence à laquelle vous avez ressenti ce bien-être.

Exemple : Lundi, 18.01.16

Corps cétoniques (mg/dl): ☐ 0 ☐ 5 ☐ 15 ☒ 40 ☐ 80 ☐ 160

○ **Régime :** ☐ 800 kcal ☐ 1.200 kcal ☐ 1.800 kcal

Suppléments : ☐ non ☐ poisson
☐ autre : ______________________

○ **Jour de repos digestif :** ☐ riz ☐ pommes de terre/légumes
☐ fruits ☐ autre: ______________

☒ **Jeûne :** miel ☐ non ☒ oui

midi : jus (¼l) ☐ non ☒ oui
bouillon (¼l) ☒ non ☐ oui

soir : bouillon (¼l) ☐ non ☒ oui

suppléments: ☒ non ☐ Kousmine ☐ Kousmine vegan
☐ yoghourt ☐ décoction d'avoine
☐ décoction de riz ☐ jus de carotte
☐ thé vert ☐ thé noir
☐ autre : ______________________

○ **Rupture du jeûne**

○ **Jour de réalimentation :** ☐ 1 ☐ 2 ☐ 3 ☐ 4

Votre état de santé physique :

très mauvais	0	1	2 (X)	3	4	5	6	7	8	9	10	excellent

Votre état de santé émotionnelle :

très mauvais	0	1	2	3	4	5	6	7	8 (X)	9	10	excellent

EXEMPLE

Symptômes depuis l'arrivée à la clinique: ☒ non ☐ oui

- ☐ maux de tête
- ☐ trouble du sommeil
- ☐ haleine chargée (demandez à l'infirmière)
- ☐ fatigue
- ☐ mal de dos
- ☐ frilosité
- ☐ vertige
- ☐ faim
- ☐ éruption cutanée
- ☐ ballonnements
- ☐ douleurs musculaires
- ☐ brûlures d'estomac
- ☐ nausée
- ☐ bouche sèche
- ☐ crampe au mollet
- ☐ diarrhée
- ☐ jambes sans repos
- ☐ constipation
- ☐ palpitations
- ☐ troubles de la vue
- ☐ envie de manger
- ☐ autres: ____________________

EXEMPLE

**Aujourd'hui …**	jamais	de temps en temps	moins de la moitié du temps	plus de la moitié du temps	la plupart du temps	tout le temps
…je me suis senti(e) bien et de bonne humeur	0	1	2	3	~~4~~ ✗	5
…je me suis senti(e) calme et tranquille	0	1	~~2~~ ✗	3	4	5
…je me suis senti(e) plein(e) d'énergie et vigoureux(se)	0	1	2	3	4	~~5~~ ✗
…je me suis réveillé(e) en me sentant frais(che) et dispos(e)	~~0~~ ✗	1	2	3	4	5
…ma vie quotidienne a été remplie de choses intéressantes	0	1	2	3	~~4~~ ✗	5

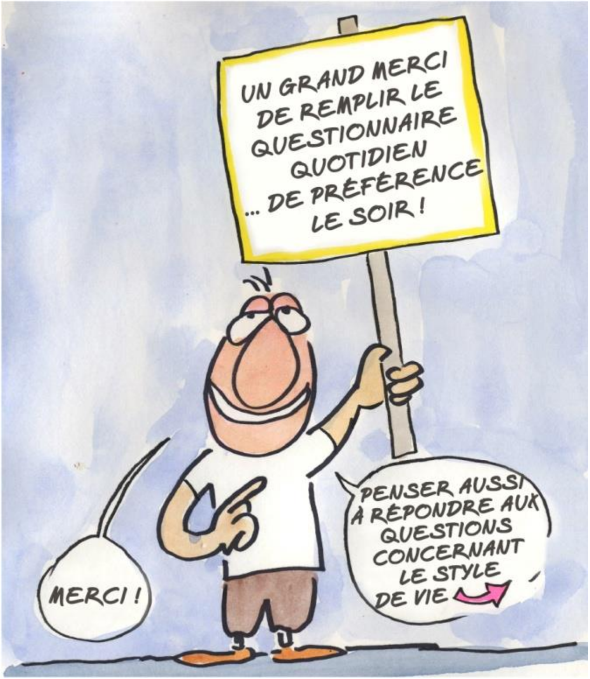
UN GRAND MERCI DE REMPLIR LE QUESTIONNAIRE QUOTIDIEN ... DE PRÉFÉRENCE LE SOIR !
MERCI !
PENSER AUSSI À RÉPONDRE AUX QUESTIONS CONCERNANT LE STYLE DE VIE

Style de vie – avant le séjour

Niveau de stress/relaxation :

état de tension extrême	0	1	2	3	4	5	6	7	8	9	10	totalement relaxé

**Nicotine :**	☐ non	☐ cigarettes/jour:	☐☐
	☐ non	☐ cigares/jour:	☐☐
**Alcool :**	☐ non	☐ bière : verre (0,5 l)/semaine:	☐☐
	☐ non	☐ vin : verre (0,25 l)/semaine:	☐☐
	☐ non	☐ alcool fort : verre (2 cl)/semaine:	☐☐
**Café :**	☐ non	☐ tasse/jour:	☐☐
**Médias :**	☐ non	☐ smart-phone (h/semaine):	☐☐
	☐ non	☐ télévision (h/semaine):	☐☐
	☐ non	☐ ordinateur (h/semaine):	☐☐
**Exercice physique :**	☐ non	☐ oui : h/semaine:	☐☐ , ☐

**Au cours des deux dernières semaines avant mon arrivée ...**	jamais	de temps en temps	moins de la moitié du temps	plus de la moitié du temps	la plupart du temps	tout le temps
...je me suis senti(e) bien et de bonne humeur	0	1	2	3	4	5
...je me suis senti(e) calme et tranquille	0	1	2	3	4	5
...je me suis senti(e) plein(e) d'énerqie et vigoureux(se)	0	1	2	3	4	5
...je me suis réveillé(e) en me sentant frais(che) et dispos(e)	0	1	2	3	4	5
...ma vie quotidienne a été remplie de choses intéressantes	0	1	2	3	4	5

1. jour (jour d'arrivée): ____________________

Corps cétoniques (mg/dl): ☐ 0 ☐ 5 ☐ 15 ☐ 40 ☐ 80 ☐ 160

- **Régime :** ☐ 800 kcal ☐ 1.200 kcal ☐ 1.800 kcal

  suppléments : ☐ non ☐ poisson
  ☐ autre : ____________________

- **Jour de repos digestif :** ☐ riz ☐ pommes de terre/légumes
  ☐ fruits ☐ autre : ____________________

- **Jeûne :**

	miel	☐ non	☐ oui
midi :	jus (¼l)	☐ non	☐ oui
	bouillon (¼l)	☐ non	☐ oui
soir :	bouillon (¼l)	☐ non	☐ oui

  suppléments: ☐ non ☐ Kousmine ☐ Kousmine vegan
  ☐ yoghourt ☐ décoction d'avoine
  ☐ décoction de riz ☐ jus de carotte
  ☐ thé vert ☐ thé noir
  ☐ autre : ____________________

- **Rupture du jeûne**

- **Jour de réalimentation :** ☐ 1 ☐ 2 ☐ 3 ☐ 4

Votre état de santé physique :

très mauvais	0	1	2	3	4	5	6	7	8	9	10	excellent

Votre état de santé émotionnelle :

très mauvais	0	1	2	3	4	5	6	7	8	9	10	excellent

Symptômes depuis l'arrivée à la clinique: ☐ non ☐ oui

- ☐ maux de tête
- ☐ trouble du sommeil
- ☐ haleine chargée
  (demandez à l'infirmière)
- ☐ fatigue
- ☐ mal de dos
- ☐ frilosité
- ☐ vertige
- ☐ faim
- ☐ éruption cutanée
- ☐ ballonnements
- ☐ douleurs musculaires
- ☐ brûlures d'estomac
- ☐ nausée
- ☐ bouche sèche
- ☐ crampe au mollet
- ☐ diarrhée
- ☐ jambes sans repos
- ☐ constipation
- ☐ palpitations
- ☐ troubles de la vue
- ☐ envie de manger
- ☐ autres: ______________________

**Aujourd'hui …**	jamais	de temps en temps	moins de la moitié du temps	plus de la moitié du temps	la plupart du temps	tout le temps
…je me suis senti(e) bien et de bonne humeur	0	1	2	3	4	5
…je me suis senti(e) calme et tranquille	0	1	2	3	4	5
…je me suis senti(e) plein(e) d'énerqie et vigoureux(se)	0	1	2	3	4	5
…je me suis réveillé(e) en me sentant frais(che) et dispos(e)	0	1	2	3	4	5
…ma vie quotidienne a été remplie de choses intéressantes	0	1	2	3	4	5

2. jour : ____________________

Corps cétoniques (mg/dl): ☐ 0 ☐ 5 ☐ 15 ☐ 40 ☐ 80 ☐ 160

○ **Régime :** ☐ 800 kcal ☐ 1.200 kcal ☐ 1.800 kcal

suppléments : ☐ non ☐ poisson
☐ autre : ____________________

○ **Jour de repos digestif :** ☐ riz ☐ pommes de terre/légumes
☐ fruits ☐ autre : ____________________

○ **Jeûne :**

	miel	☐ non	☐ oui
midi :	jus (¼l)	☐ non	☐ oui
	bouillon (¼l)	☐ non	☐ oui
soir :	bouillon (¼l)	☐ non	☐ oui

suppléments: ☐ non ☐ Kousmine ☐ Kousmine vegan
☐ yoghourt ☐ décoction d'avoine
☐ décoction de riz ☐ jus de carotte
☐ thé vert ☐ thé noir
☐ autre : ____________________

○ **Rupture du jeûne**

○ **Jour de réalimentation :** ☐ 1 ☐ 2 ☐ 3 ☐ 4

Votre état de santé physique :

très mauvais	0	1	2	3	4	5	6	7	8	9	10	excellent

Votre état de santé émotionnelle :

très mauvais	0	1	2	3	4	5	6	7	8	9	10	excellent

Symptômes depuis l'arrivée à la clinique: ☐ non ☐ oui

- ☐ maux de tête
- ☐ trouble du sommeil
- ☐ haleine chargée (demandez à l'infirmière)
- ☐ fatigue
- ☐ mal de dos
- ☐ frilosité
- ☐ vertige
- ☐ faim
- ☐ éruption cutanée
- ☐ ballonnements
- ☐ douleurs musculaires
- ☐ brûlures d'estomac
- ☐ nausée
- ☐ bouche sèche
- ☐ crampe au mollet
- ☐ diarrhée
- ☐ jambes sans repos
- ☐ constipation
- ☐ palpitations
- ☐ troubles de la vue
- ☐ envie de manger
- ☐ autres: ______________________________

**Aujourd'hui …**	jamais	de temps en temps	moins de la moitié du temps	plus de la moitié du temps	la plupart du temps	tout le temps
…je me suis senti(e) bien et de bonne humeur	0	1	2	3	4	5
…je me suis senti(e) calme et tranquille	0	1	2	3	4	5
…je me suis senti(e) plein(e) d'énergie et vigoureux(se)	0	1	2	3	4	5
…je me suis réveillé(e) en me sentant frais(che) et dispos(e)	0	1	2	3	4	5
…ma vie quotidienne a été remplie de choses intéressantes	0	1	2	3	4	5

3. jour : ______________________

Corps cétoniques (mg/dl): ☐ 0 ☐ 5 ☐ 15 ☐ 40 ☐ 80 ☐ 160

- **Régime :** ☐ 800 kcal ☐ 1.200 kcal ☐ 1.800 kcal

  suppléments : ☐ non ☐ poisson
  ☐ autre : ______________________

- **Jour de repos digestif :** ☐ riz ☐ pommes de terre/légumes
  ☐ fruits ☐ autre : ______________

- **Jeûne :**

	miel	☐ non	☐ oui
midi :	jus (¼l)	☐ non	☐ oui
	bouillon (¼l)	☐ non	☐ oui
soir :	bouillon (¼l)	☐ non	☐ oui

  suppléments: ☐ non ☐ Kousmine ☐ Kousmine vegan
  ☐ yoghourt ☐ décoction d'avoine
  ☐ décoction de riz ☐ jus de carotte
  ☐ thé vert ☐ thé noir
  ☐ autre : ______________________

- **Rupture du jeûne**

- **Jour de réalimentation :** ☐ 1 ☐ 2 ☐ 3 ☐ 4

Votre état de santé physique :

très mauvais	0	1	2	3	4	5	6	7	8	9	10	excellent

Votre état de santé émotionnelle :

très mauvais	0	1	2	3	4	5	6	7	8	9	10	excellent

Symptômes depuis l'arrivée à la clinique: ☐ non ☐ oui

- ☐ maux de tête
- ☐ trouble du sommeil
- ☐ haleine chargée
  (demandez à l'infirmière)
- ☐ fatigue
- ☐ mal de dos
- ☐ frilosité
- ☐ vertige
- ☐ faim
- ☐ éruption cutanée
- ☐ ballonnements
- ☐ douleurs musculaires
- ☐ brûlures d'estomac
- ☐ nausée
- ☐ bouche sèche
- ☐ crampe au mollet
- ☐ diarrhée
- ☐ jambes sans repos
- ☐ constipation
- ☐ palpitations
- ☐ troubles de la vue
- ☐ envie de manger
- ☐ autres: ______________________________

**Aujourd'hui …**	jamais	de temps en temps	moins de la moitié du temps	plus de la moitié du temps	la plupart du temps	tout le temps
…je me suis senti(e) bien et de bonne humeur	0	1	2	3	4	5
…je me suis senti(e) calme et tranquille	0	1	2	3	4	5
…je me suis senti(e) plein(e) d'énerqie et vigoureux(se)	0	1	2	3	4	5
…je me suis réveillé(e) en me sentant frais(che) et dispos(e)	0	1	2	3	4	5
…ma vie quotidienne a été remplie de choses intéressantes	0	1	2	3	4	5

4. jour : ______________________________

Corps cétoniques (mg/dl): ☐ 0 ☐ 5 ☐ 15 ☐ 40 ☐ 80 ☐ 160

○ **Régime :** ☐ 800 kcal ☐ 1.200 kcal ☐ 1.800 kcal

suppléments : ☐ non ☐ poisson
☐ autre : ______________________________

○ **Jour de repos digestif :** ☐ riz ☐ pommes de terre/légumes
☐ fruits ☐ autre : ______________

○ **Jeûne :**

	miel	☐ non	☐ oui
midi :	jus (¼l)	☐ non	☐ oui
	bouillon (¼l)	☐ non	☐ oui
soir :	bouillon (¼l)	☐ non	☐ oui

suppléments: ☐ non ☐ Kousmine ☐ Kousmine vegan
☐ yoghourt ☐ décoction d'avoine
☐ décoction de riz ☐ jus de carotte
☐ thé vert ☐ thé noir
☐ autre : ______________________________

○ **Rupture du jeûne**

○ **Jour de réalimentation :** ☐ 1 ☐ 2 ☐ 3 ☐ 4

Votre état de santé physique :

très mauvais	0	1	2	3	4	5	6	7	8	9	10	excellent

Votre état de santé émotionnelle :

très mauvais	0	1	2	3	4	5	6	7	8	9	10	excellent

Symptômes depuis l'arrivée à la clinique: ☐ non ☐ oui

☐ maux de tête
☐ trouble du sommeil
☐ haleine chargée
(demandez à l'infirmière)
☐ fatigue
☐ mal de dos
☐ frilosité
☐ vertige
☐ faim
☐ éruption cutanée
☐ ballonnements
☐ douleurs musculaires
☐ brûlures d'estomac
☐ nausée
☐ bouche sèche
☐ crampe au mollet
☐ diarrhée
☐ jambes sans repos
☐ constipation
☐ palpitations
☐ troubles de la vue
☐ envie de manger
☐ autres: ______________________________

**Aujourd'hui …**	jamais	de temps en temps	moins de la moitié du temps	plus de la moitié du temps	la plupart du temps	tout le temps
…je me suis senti(e) bien et de bonne humeur	0	1	2	3	4	5
…je me suis senti(e) calme et tranquille	0	1	2	3	4	5
…je me suis senti(e) plein(e) d'énerqie et vigoureux(se)	0	1	2	3	4	5
…je me suis réveillé(e) en me sentant frais(che) et dispos(e)	0	1	2	3	4	5
…ma vie quotidienne a été remplie de choses intéressantes	0	1	2	3	4	5

5. jour : ______________________________

Corps cétoniques (mg/dl): ☐ 0 ☐ 5 ☐ 15 ☐ 40 ☐ 80 ☐ 160

○ **Régime :** ☐ 800 kcal ☐ 1.200 kcal ☐ 1.800 kcal

suppléments : ☐ non ☐ poisson
☐ autre : ______________________________

○ **Jour de repos digestif :** ☐ riz ☐ pommes de terre/légumes
☐ fruits ☐ autre : ____________________

○ **Jeûne :** miel ☐ non ☐ oui

midi :	jus (¼l)	☐ non	☐ oui
	bouillon (¼l)	☐ non	☐ oui
soir :	bouillon (¼l)	☐ non	☐ oui

suppléments: ☐ non ☐ Kousmine ☐ Kousmine vegan
☐ yoghourt ☐ décoction d'avoine
☐ décoction de riz ☐ jus de carotte
☐ thé vert ☐ thé noir
☐ autre : ______________________________

○ **Rupture du jeûne**

○ **Jour de réalimentation :** ☐ 1 ☐ 2 ☐ 3 ☐ 4

Votre état de santé physique :

très mauvais	0	1	2	3	4	5	6	7	8	9	10	excellent

Votre état de santé émotionnelle :

très mauvais	0	1	2	3	4	5	6	7	8	9	10	excellent

Symptômes depuis l'arrivée à la clinique: ☐ non ☐ oui

- ☐ maux de tête
- ☐ trouble du sommeil
- ☐ haleine chargée (demandez à l'infirmière)
- ☐ fatigue
- ☐ mal de dos
- ☐ frilosité
- ☐ vertige
- ☐ faim
- ☐ éruption cutanée
- ☐ ballonnements
- ☐ douleurs musculaires
- ☐ brûlures d'estomac
- ☐ nausée
- ☐ bouche sèche
- ☐ crampe au mollet
- ☐ diarrhée
- ☐ jambes sans repos
- ☐ constipation
- ☐ palpitations
- ☐ troubles de la vue
- ☐ envie de manger
- ☐ autres: ____________________

**Aujourd'hui …**	jamais	de temps en temps	moins de la moitié du temps	plus de la moitié du temps	la plupart du temps	tout le temps
…je me suis senti(e) bien et de bonne humeur	0	1	2	3	4	5
…je me suis senti(e) calme et tranquille	0	1	2	3	4	5
…je me suis senti(e) plein(e) d'énerqie et vigoureux(se)	0	1	2	3	4	5
…je me suis réveillé(e) en me sentant frais(che) et dispos(e)	0	1	2	3	4	5
…ma vie quotidienne a été remplie de choses intéressantes	0	1	2	3	4	5

6. jour : ______________________________

Corps cétoniques (mg/dl): ☐ 0 ☐ 5 ☐ 15 ☐ 40 ☐ 80 ☐ 160

○ **Régime :** ☐ 800 kcal ☐ 1.200 kcal ☐ 1.800 kcal

suppléments : ☐ non ☐ poisson
☐ autre : ______________________________

○ **Jour de repos digestif :** ☐ riz ☐ pommes de terre/légumes
☐ fruits ☐ autre : ____________________

○ **Jeûne :** miel ☐ non ☐ oui

midi : jus (¼l) ☐ non ☐ oui
bouillon (¼l) ☐ non ☐ oui

soir : bouillon (¼l) ☐ non ☐ oui

suppléments: ☐ non ☐ Kousmine ☐ Kousmine vegan
☐ yoghourt ☐ décoction d'avoine
☐ décoction de riz ☐ jus de carotte
☐ thé vert ☐ thé noir
☐ autre : ____________________

○ **Rupture du jeûne**

○ **Jour de réalimentation :** ☐ 1 ☐ 2 ☐ 3 ☐ 4

Votre état de santé physique :

très mauvais	0	1	2	3	4	5	6	7	8	9	10	excellent

Votre état de santé émotionnelle :

très mauvais	0	1	2	3	4	5	6	7	8	9	10	excellent

Symptômes depuis l'arrivée à la clinique: ☐ non ☐ oui

- ☐ maux de tête
- ☐ trouble du sommeil
- ☐ haleine chargée (demandez à l'infirmière)
- ☐ fatigue
- ☐ mal de dos
- ☐ frilosité
- ☐ vertige
- ☐ faim
- ☐ éruption cutanée
- ☐ ballonnements
- ☐ douleurs musculaires
- ☐ brûlures d'estomac
- ☐ nausée
- ☐ bouche sèche
- ☐ crampe au mollet
- ☐ diarrhée
- ☐ jambes sans repos
- ☐ constipation
- ☐ palpitations
- ☐ troubles de la vue
- ☐ envie de manger
- ☐ autres: ____________________

**Aujourd'hui …**	jamais	de temps en temps	moins de la moitié du temps	plus de la moitié du temps	la plupart du temps	tout le temps
…je me suis senti(e) bien et de bonne humeur	0	1	2	3	4	5
…je me suis senti(e) calme et tranquille	0	1	2	3	4	5
…je me suis senti(e) plein(e) d'énerqie et vigoureux(se)	0	1	2	3	4	5
…je me suis réveillé(e) en me sentant frais(che) et dispos(e)	0	1	2	3	4	5
…ma vie quotidienne a été remplie de choses intéressantes	0	1	2	3	4	5

7. jour : ______________________________

Corps cétoniques (mg/dl): ☐ 0 ☐ 5 ☐ 15 ☐ 40 ☐ 80 ☐ 160

○ **Régime :** ☐ 800 kcal ☐ 1.200 kcal ☐ 1.800 kcal

suppléments : ☐ non ☐ poisson
☐ autre : ______________________

○ **Jour de repos digestif :** ☐ riz ☐ pommes de terre/légumes
☐ fruits ☐ autre : ______________

○ **Jeûne :**

	miel	☐ non	☐ oui
midi :	jus (¼l)	☐ non	☐ oui
	bouillon (¼l)	☐ non	☐ oui
soir :	bouillon (¼l)	☐ non	☐ oui

suppléments: ☐ non
☐ Kousmine ☐ Kousmine vegan
☐ yoghourt ☐ décoction d'avoine
☐ décoction de riz ☐ jus de carotte
☐ thé vert ☐ thé noir
☐ autre : ______________________

○ **Rupture du jeûne**

○ **Jour de réalimentation :** ☐ 1 ☐ 2 ☐ 3 ☐ 4

Votre état de santé physique :

très mauvais	0	1	2	3	4	5	6	7	8	9	10	excellent

Votre état de santé émotionnelle :

très mauvais	0	1	2	3	4	5	6	7	8	9	10	excellent

Symptômes depuis l'arrivée à la clinique: ☐ non ☐ oui

- ☐ maux de tête
- ☐ trouble du sommeil
- ☐ haleine chargée (demandez à l'infirmière)
- ☐ fatigue
- ☐ mal de dos
- ☐ frilosité
- ☐ vertige
- ☐ faim
- ☐ éruption cutanée
- ☐ ballonnements
- ☐ douleurs musculaires
- ☐ brûlures d'estomac
- ☐ nausée
- ☐ bouche sèche
- ☐ crampe au mollet
- ☐ diarrhée
- ☐ jambes sans repos
- ☐ constipation
- ☐ palpitations
- ☐ troubles de la vue
- ☐ envie de manger
- ☐ autres: ____________________

**Aujourd'hui …**	jamais	de temps en temps	moins de la moitié du temps	plus de la moitié du temps	la plupart du temps	tout le temps
…je me suis senti(e) bien et de bonne humeur	0	1	2	3	4	5
…je me suis senti(e) calme et tranquille	0	1	2	3	4	5
…je me suis senti(e) plein(e) d'énerqie et vigoureux(se)	0	1	2	3	4	5
…je me suis réveillé(e) en me sentant frais(che) et dispos(e)	0	1	2	3	4	5
…ma vie quotidienne a été remplie de choses intéressantes	0	1	2	3	4	5

8. jour : ______________________________

Corps cétoniques (mg/dl): ☐ 0 ☐ 5 ☐ 15 ☐ 40 ☐ 80 ☐ 160

○ **Régime :** ☐ 800 kcal ☐ 1.200 kcal ☐ 1.800 kcal

suppléments : ☐ non ☐ poisson
☐ autre : ______________________

○ **Jour de repos digestif :** ☐ riz ☐ pommes de terre/légumes
☐ fruits ☐ autre : ______________

○ **Jeûne :**

	miel	☐ non	☐ oui
midi :	jus (¼l)	☐ non	☐ oui
	bouillon (¼l)	☐ non	☐ oui
soir :	bouillon (¼l)	☐ non	☐ oui

suppléments: ☐ non ☐ Kousmine ☐ Kousmine vegan
☐ yoghourt ☐ décoction d'avoine
☐ décoction de riz ☐ jus de carotte
☐ thé vert ☐ thé noir
☐ autre : ______________________

○ **Rupture du jeûne**

○ **Jour de réalimentation :** ☐ 1 ☐ 2 ☐ 3 ☐ 4

Votre état de santé physique :

très mauvais	0	1	2	3	4	5	6	7	8	9	10	excellent

Votre état de santé émotionnelle :

très mauvais	0	1	2	3	4	5	6	7	8	9	10	excellent

Symptômes depuis l'arrivée à la clinique: ☐ non ☐ oui

☐ maux de tête	☐ douleurs musculaires
☐ trouble du sommeil	☐ brûlures d'estomac
☐ haleine chargée (demandez à l'infirmière)	☐ nausée
	☐ bouche sèche
☐ fatigue	☐ crampe au mollet
☐ mal de dos	☐ diarrhée
☐ frilosité	☐ jambes sans repos
☐ vertige	☐ constipation
☐ faim	☐ palpitations
☐ éruption cutanée	☐ troubles de la vue
☐ ballonnements	☐ envie de manger

☐ autres: ______________________

**Aujourd'hui ...**	jamais	de temps en temps	moins de la moitié du temps	plus de la moitié du temps	la plupart du temps	tout le temps
...je me suis senti(e) bien et de bonne humeur	0	1	2	3	4	5
...je me suis senti(e) calme et tranquille	0	1	2	3	4	5
...je me suis senti(e) plein(e) d'énerqie et vigoureux(se)	0	1	2	3	4	5
...je me suis réveillé(e) en me sentant frais(che) et dispos(e)	0	1	2	3	4	5
...ma vie quotidienne a été remplie de choses intéressantes	0	1	2	3	4	5

9. jour : ______________________

Corps cétoniques (mg/dl): ☐ 0 ☐ 5 ☐ 15 ☐ 40 ☐ 80 ☐ 160

○ **Régime :** ☐ 800 kcal ☐ 1.200 kcal ☐ 1.800 kcal

suppléments : ☐ non ☐ poisson
☐ autre :______________________

○ **Jour de repos digestif :** ☐ riz ☐ pommes de terre/légumes
☐ fruits ☐ autre : ______________

○ **Jeûne :**

	miel	☐ non	☐ oui
midi :	jus (¼l)	☐ non	☐ oui
	bouillon (¼l)	☐ non	☐ oui
soir :	bouillon (¼l)	☐ non	☐ oui

suppléments: ☐ non ☐ Kousmine ☐ Kousmine vegan
☐ yoghourt ☐ décoction d'avoine
☐ décoction de riz ☐ jus de carotte
☐ thé vert ☐ thé noir
☐ autre :______________________

○ **Rupture du jeûne**

○ **Jour de réalimentation :** ☐ 1 ☐ 2 ☐ 3 ☐ 4

Votre état de santé physique :

très mauvais	0	1	2	3	4	5	6	7	8	9	10	excellent

Votre état de santé émotionnelle :

très mauvais	0	1	2	3	4	5	6	7	8	9	10	excellent

Symptômes depuis l’arrivée à la clinique: ☐ non ☐ oui

☐ maux de tête
☐ trouble du sommeil
☐ haleine chargée
(demandez à l’infirmière)
☐ fatigue
☐ mal de dos
☐ frilosité
☐ vertige
☐ faim
☐ éruption cutanée
☐ ballonnements
☐ douleurs musculaires
☐ brûlures d’estomac
☐ nausée
☐ bouche sèche
☐ crampe au mollet
☐ diarrhée
☐ jambes sans repos
☐ constipation
☐ palpitations
☐ troubles de la vue
☐ envie de manger
☐ autres: ______

**Aujourd’hui ...**	jamais	de temps en temps	moins de la moitié du temps	plus de la moitié du temps	la plupart du temps	tout le temps
...je me suis senti(e) bien et de bonne humeur	0	1	2	3	4	5
...je me suis senti(e) calme et tranquille	0	1	2	3	4	5
...je me suis senti(e) plein(e) d’énergie et vigoureux(se)	0	1	2	3	4	5
...je me suis réveillé(e) en me sentant frais(che) et dispos(e)	0	1	2	3	4	5
...ma vie quotidienne a été remplie de choses intéressantes	0	1	2	3	4	5

10. jour : ____________________

Corps cétoniques (mg/dl): ☐ 0 ☐ 5 ☐ 15 ☐ 40 ☐ 80 ☐ 160

- **Régime :** ☐ 800 kcal ☐ 1.200 kcal ☐ 1.800 kcal

  suppléments : ☐ non ☐ poisson
  ☐ autre : ____________________

- **Jour de repos digestif :** ☐ riz ☐ pommes de terre/légumes
  ☐ fruits ☐ autre : ____________________

- **Jeûne :** miel ☐ non ☐ oui

  midi : jus (¼l) ☐ non ☐ oui
  bouillon (¼l) ☐ non ☐ oui
  soir : bouillon (¼l) ☐ non ☐ oui

  suppléments: ☐ non ☐ Kousmine ☐ Kousmine vegan
  ☐ yoghourt ☐ décoction d'avoine
  ☐ décoction de riz ☐ jus de carotte
  ☐ thé vert ☐ thé noir
  ☐ autre : ____________________

- **Rupture du jeûne**

- **Jour de réalimentation :** ☐ 1 ☐ 2 ☐ 3 ☐ 4

Votre état de santé physique :

très mauvais	0	1	2	3	4	5	6	7	8	9	10	excellent

Votre état de santé émotionnelle :

très mauvais	0	1	2	3	4	5	6	7	8	9	10	excellent

Symptômes depuis l'arrivée à la clinique: ☐ non ☐ oui

- ☐ maux de tête
- ☐ trouble du sommeil
- ☐ haleine chargée (demandez à l'infirmière)
- ☐ fatigue
- ☐ mal de dos
- ☐ frilosité
- ☐ vertige
- ☐ faim
- ☐ éruption cutanée
- ☐ ballonnements
- ☐ douleurs musculaires
- ☐ brûlures d'estomac
- ☐ nausée
- ☐ bouche sèche
- ☐ crampe au mollet
- ☐ diarrhée
- ☐ jambes sans repos
- ☐ constipation
- ☐ palpitations
- ☐ troubles de la vue
- ☐ envie de manger
- ☐ autres: ____________________

**Aujourd'hui …**	jamais	de temps en temps	moins de la moitié du temps	plus de la moitié du temps	la plupart du temps	tout le temps
…je me suis senti(e) bien et de bonne humeur	0	1	2	3	4	5
…je me suis senti(e) calme et tranquille	0	1	2	3	4	5
…je me suis senti(e) plein(e) d'énergie et vigoureux(se)	0	1	2	3	4	5
…je me suis réveillé(e) en me sentant frais(che) et dispos(e)	0	1	2	3	4	5
…ma vie quotidienne a été remplie de choses intéressantes	0	1	2	3	4	5

11. jour : ______________________________

Corps cétoniques (mg/dl): ☐ 0 ☐ 5 ☐ 15 ☐ 40 ☐ 80 ☐ 160

○ **Régime :** ☐ 800 kcal ☐ 1.200 kcal ☐ 1.800 kcal

suppléments : ☐ non ☐ poisson
☐ autre : ______________________

○ **Jour de repos digestif :** ☐ riz ☐ pommes de terre/légumes
☐ fruits ☐ autre : ______________

○ **Jeûne :**

	miel	☐ non	☐ oui
midi :	jus (¼l)	☐ non	☐ oui
	bouillon (¼l)	☐ non	☐ oui
soir :	bouillon (¼l)	☐ non	☐ oui

suppléments: ☐ non ☐ Kousmine ☐ Kousmine vegan
☐ yoghourt ☐ décoction d'avoine
☐ décoction de riz ☐ jus de carotte
☐ thé vert ☐ thé noir
☐ autre : ______________________

○ **Rupture du jeûne**

○ **Jour de réalimentation :** ☐ 1 ☐ 2 ☐ 3 ☐ 4

Votre état de santé physique :

très mauvais	0	1	2	3	4	5	6	7	8	9	10	excellent

Votre état de santé émotionnelle :

très mauvais	0	1	2	3	4	5	6	7	8	9	10	excellent

Symptômes depuis l'arrivée à la clinique: ☐ non ☐ oui

☐ maux de tête
☐ trouble du sommeil
☐ haleine chargée
(demandez à l'infirmière)
☐ fatigue
☐ mal de dos
☐ frilosité
☐ vertige
☐ faim
☐ éruption cutanée
☐ ballonnements
☐ douleurs musculaires
☐ brûlures d'estomac
☐ nausée
☐ bouche sèche
☐ crampe au mollet
☐ diarrhée
☐ jambes sans repos
☐ constipation
☐ palpitations
☐ troubles de la vue
☐ envie de manger
☐ autres: ____________________

**Aujourd'hui …**	jamais	de temps en temps	moins de la moitié du temps	plus de la moitié du temps	la plupart du temps	tout le temps
…je me suis senti(e) bien et de bonne humeur	0	1	2	3	4	5
…je me suis senti(e) calme et tranquille	0	1	2	3	4	5
…je me suis senti(e) plein(e) d'énerqie et vigoureux(se)	0	1	2	3	4	5
…je me suis réveillé(e) en me sentant frais(che) et dispos(e)	0	1	2	3	4	5
…ma vie quotidienne a été remplie de choses intéressantes	0	1	2	3	4	5

12. jour : ______________________________

Corps cétoniques (mg/dl): ☐ 0 ☐ 5 ☐ 15 ☐ 40 ☐ 80 ☐ 160

○ **Régime :** ☐ 800 kcal ☐ 1.200 kcal ☐ 1.800 kcal

suppléments : ☐ non ☐ poisson
☐ autre : ______________________

○ **Jour de repos digestif :** ☐ riz ☐ pommes de terre/légumes
☐ fruits ☐ autre : ______________

○ **Jeûne :**

	miel	☐ non	☐ oui
midi :	jus (¼l)	☐ non	☐ oui
	bouillon (¼l)	☐ non	☐ oui
soir :	bouillon (¼l)	☐ non	☐ oui

suppléments: ☐ non ☐ Kousmine ☐ Kousmine vegan
☐ yoghourt ☐ décoction d'avoine
☐ décoction de riz ☐ jus de carotte
☐ thé vert ☐ thé noir
☐ autre : ______________________

○ **Rupture du jeûne**

○ **Jour de réalimentation :** ☐ 1 ☐ 2 ☐ 3 ☐ 4

Votre état de santé physique :

très mauvais	0	1	2	3	4	5	6	7	8	9	10	excellent

Votre état de santé émotionnelle :

très mauvais	0	1	2	3	4	5	6	7	8	9	10	excellent

Symptômes depuis l'arrivée à la clinique: ☐ non ☐ oui

☐ maux de tête
☐ trouble du sommeil
☐ haleine chargée
(demandez à l'infirmière)
☐ fatigue
☐ mal de dos
☐ frilosité
☐ vertige
☐ faim
☐ éruption cutanée
☐ ballonnements
☐ douleurs musculaires
☐ brûlures d'estomac
☐ nausée
☐ bouche sèche
☐ crampe au mollet
☐ diarrhée
☐ jambes sans repos
☐ constipation
☐ palpitations
☐ troubles de la vue
☐ envie de manger
☐ autres: ____________________

**Aujourd'hui …**	jamais	de temps en temps	moins de la moitié du temps	plus de la moitié du temps	la plupart du temps	tout le temps
…je me suis senti(e) bien et de bonne humeur	0	1	2	3	4	5
…je me suis senti(e) calme et tranquille	0	1	2	3	4	5
…je me suis senti(e) plein(e) d'énerqie et vigoureux(se)	0	1	2	3	4	5
…je me suis réveillé(e) en me sentant frais(che) et dispos(e)	0	1	2	3	4	5
…ma vie quotidienne a été remplie de choses intéressantes	0	1	2	3	4	5

13. jour : ____________________

Corps cétoniques (mg/dl): ☐ 0 ☐ 5 ☐ 15 ☐ 40 ☐ 80 ☐ 160

- **Régime :** ☐ 800 kcal ☐ 1.200 kcal ☐ 1.800 kcal

  suppléments : ☐ non ☐ poisson
  ☐ autre : ____________________

- **Jour de repos digestif :** ☐ riz ☐ pommes de terre/légumes
  ☐ fruits ☐ autre : ____________________

- **Jeûne :**

	miel	☐ non	☐ oui
midi :	jus (¼l)	☐ non	☐ oui
	bouillon (¼l)	☐ non	☐ oui
soir :	bouillon (¼l)	☐ non	☐ oui

  suppléments: ☐ non ☐ Kousmine ☐ Kousmine vegan
  ☐ yoghourt ☐ décoction d'avoine
  ☐ décoction de riz ☐ jus de carotte
  ☐ thé vert ☐ thé noir
  ☐ autre : ____________________

- **Rupture du jeûne**

- **Jour de réalimentation :** ☐ 1 ☐ 2 ☐ 3 ☐ 4

Votre état de santé physique :

très mauvais	0	1	2	3	4	5	6	7	8	9	10	excellent

Votre état de santé émotionnelle :

très mauvais	0	1	2	3	4	5	6	7	8	9	10	excellent

Symptômes depuis l'arrivée à la clinique: ☐ non ☐ oui

- ☐ maux de tête
- ☐ trouble du sommeil
- ☐ haleine chargée (demandez à l'infirmière)
- ☐ fatigue
- ☐ mal de dos
- ☐ frilosité
- ☐ vertige
- ☐ faim
- ☐ éruption cutanée
- ☐ ballonnements
- ☐ douleurs musculaires
- ☐ brûlures d'estomac
- ☐ nausée
- ☐ bouche sèche
- ☐ crampe au mollet
- ☐ diarrhée
- ☐ jambes sans repos
- ☐ constipation
- ☐ palpitations
- ☐ troubles de la vue
- ☐ envie de manger
- ☐ autres: ____________________

**Aujourd'hui …**	jamais	de temps en temps	moins de la moitié du temps	plus de la moitié du temps	la plupart du temps	tout le temps
…je me suis senti(e) bien et de bonne humeur	0	1	2	3	4	5
…je me suis senti(e) calme et tranquille	0	1	2	3	4	5
…je me suis senti(e) plein(e) d'énerqie et vigoureux(se)	0	1	2	3	4	5
…je me suis réveillé(e) en me sentant frais(che) et dispos(e)	0	1	2	3	4	5
…ma vie quotidienne a été remplie de choses intéressantes	0	1	2	3	4	5

14. jour : ______________________________

Corps cétoniques (mg/dl): ☐ 0 ☐ 5 ☐ 15 ☐ 40 ☐ 80 ☐ 160

○ **Régime :** ☐ 800 kcal ☐ 1.200 kcal ☐ 1.800 kcal

suppléments : ☐ non ☐ poisson
☐ autre : ______________________________

○ **Jour de repos digestif :** ☐ riz ☐ pommes de terre/légumes
☐ fruits ☐ autre : ____________________

○ **Jeûne :**

	miel	☐ non	☐ oui
midi :	jus (¼l)	☐ non	☐ oui
	bouillon (¼l)	☐ non	☐ oui
soir :	bouillon (¼l)	☐ non	☐ oui

suppléments: ☐ non ☐ Kousmine ☐ Kousmine vegan
☐ yoghourt ☐ décoction d'avoine
☐ décoction de riz ☐ jus de carotte
☐ thé vert ☐ thé noir
☐ autre : ______________________________

○ **Rupture du jeûne**

○ **Jour de réalimentation :** ☐ 1 ☐ 2 ☐ 3 ☐ 4

Votre état de santé physique :

très mauvais	0	1	2	3	4	5	6	7	8	9	10	excellent

Votre état de santé émotionnelle :

très mauvais	0	1	2	3	4	5	6	7	8	9	10	excellent

Symptômes depuis l'arrivée à la clinique: ☐ non ☐ oui

- ☐ maux de tête
- ☐ trouble du sommeil
- ☐ haleine chargée (demandez à l'infirmière)
- ☐ fatigue
- ☐ mal de dos
- ☐ frilosité
- ☐ vertige
- ☐ faim
- ☐ éruption cutanée
- ☐ ballonnements
- ☐ douleurs musculaires
- ☐ brûlures d'estomac
- ☐ nausée
- ☐ bouche sèche
- ☐ crampe au mollet
- ☐ diarrhée
- ☐ jambes sans repos
- ☐ constipation
- ☐ palpitations
- ☐ troubles de la vue
- ☐ envie de manger
- ☐ autres: ____________________

**Aujourd'hui …**	jamais	de temps en temps	moins de la moitié du temps	plus de la moitié du temps	la plupart du temps	tout le temps
…je me suis senti(e) bien et de bonne humeur	0	1	2	3	4	5
…je me suis senti(e) calme et tranquille	0	1	2	3	4	5
…je me suis senti(e) plein(e) d'énergie et vigoureux(se)	0	1	2	3	4	5
…je me suis réveillé(e) en me sentant frais(che) et dispos(e)	0	1	2	3	4	5
…ma vie quotidienne a été remplie de choses intéressantes	0	1	2	3	4	5

15. jour : ____________________

Corps cétoniques (mg/dl): ☐ 0 ☐ 5 ☐ 15 ☐ 40 ☐ 80 ☐ 160

○ **Régime :** ☐ 800 kcal ☐ 1.200 kcal ☐ 1.800 kcal

suppléments : ☐ non ☐ poisson
☐ autre : ____________________

○ **Jour de repos digestif :** ☐ riz ☐ pommes de terre/légumes
☐ fruits ☐ autre : ____________________

○ **Jeûne :**

	miel	☐ non	☐ oui
midi :	jus (¼l)	☐ non	☐ oui
	bouillon (¼l)	☐ non	☐ oui
soir :	bouillon (¼l)	☐ non	☐ oui

suppléments: ☐ non ☐ Kousmine ☐ Kousmine vegan
☐ yoghourt ☐ décoction d'avoine
☐ décoction de riz ☐ jus de carotte
☐ thé vert ☐ thé noir
☐ autre : ____________________

○ **Rupture du jeûne**

○ **Jour de réalimentation :** ☐ 1 ☐ 2 ☐ 3 ☐ 4

Votre état de santé physique :

très mauvais	0	1	2	3	4	5	6	7	8	9	10	excellent

Votre état de santé émotionnelle :

très mauvais	0	1	2	3	4	5	6	7	8	9	10	excellent

Symptômes depuis l'arrivée à la clinique: ☐ non ☐ oui

- ☐ maux de tête
- ☐ trouble du sommeil
- ☐ haleine chargée (demandez à l'infirmière)
- ☐ fatigue
- ☐ mal de dos
- ☐ frilosité
- ☐ vertige
- ☐ faim
- ☐ éruption cutanée
- ☐ ballonnements
- ☐ douleurs musculaires
- ☐ brûlures d'estomac
- ☐ nausée
- ☐ bouche sèche
- ☐ crampe au mollet
- ☐ diarrhée
- ☐ jambes sans repos
- ☐ constipation
- ☐ palpitations
- ☐ troubles de la vue
- ☐ envie de manger
- ☐ autres: ____________________

**Aujourd'hui …**	jamais	de temps en temps	moins de la moitié du temps	plus de la moitié du temps	la plupart du temps	tout le temps
…je me suis senti(e) bien et de bonne humeur	0	1	2	3	4	5
…je me suis senti(e) calme et tranquille	0	1	2	3	4	5
…je me suis senti(e) plein(e) d'énergie et vigoureux(se)	0	1	2	3	4	5
…je me suis réveillé(e) en me sentant frais(che) et dispos(e)	0	1	2	3	4	5
…ma vie quotidienne a été remplie de choses intéressantes	0	1	2	3	4	5

16. jour : ____________________

Corps cétoniques (mg/dl): ☐ 0 ☐ 5 ☐ 15 ☐ 40 ☐ 80 ☐ 160

○ **Régime :** ☐ 800 kcal ☐ 1.200 kcal ☐ 1.800 kcal

suppléments : ☐ non ☐ poisson
☐ autre : ____________________

○ **Jour de repos digestif :** ☐ riz ☐ pommes de terre/légumes
☐ fruits ☐ autre : ____________________

○ **Jeûne :** miel ☐ non ☐ oui

midi :	jus (¼l)	☐ non	☐ oui
	bouillon (¼l)	☐ non	☐ oui
soir :	bouillon (¼l)	☐ non	☐ oui

suppléments: ☐ non ☐ Kousmine ☐ Kousmine vegan
☐ yoghourt ☐ décoction d'avoine
☐ décoction de riz ☐ jus de carotte
☐ thé vert ☐ thé noir
☐ autre : ____________________

○ **Rupture du jeûne**

○ **Jour de réalimentation :** ☐ 1 ☐ 2 ☐ 3 ☐ 4

Votre état de santé physique :

très mauvais	0	1	2	3	4	5	6	7	8	9	10	excellent

Votre état de santé émotionnelle :

très mauvais	0	1	2	3	4	5	6	7	8	9	10	excellent

Symptômes depuis l'arrivée à la clinique: ☐ non ☐ oui

☐ maux de tête
☐ trouble du sommeil
☐ haleine chargée
(demandez à l'infirmière)
☐ fatigue
☐ mal de dos
☐ frilosité
☐ vertige
☐ faim
☐ éruption cutanée
☐ ballonnements
☐ douleurs musculaires
☐ brûlures d'estomac
☐ nausée
☐ bouche sèche
☐ crampe au mollet
☐ diarrhée
☐ jambes sans repos
☐ constipation
☐ palpitations
☐ troubles de la vue
☐ envie de manger
☐ autres: ____________________

**Aujourd'hui …**	jamais	de temps en temps	moins de la moitié du temps	plus de la moitié du temps	la plupart du temps	tout le temps
…je me suis senti(e) bien et de bonne humeur	0	1	2	3	4	5
…je me suis senti(e) calme et tranquille	0	1	2	3	4	5
…je me suis senti(e) plein(e) d'énergie et vigoureux(se)	0	1	2	3	4	5
…je me suis réveillé(e) en me sentant frais(che) et dispos(e)	0	1	2	3	4	5
…ma vie quotidienne a été remplie de choses intéressantes	0	1	2	3	4	5

17. jour : ____________________

Corps cétoniques (mg/dl): ☐ 0 ☐ 5 ☐ 15 ☐ 40 ☐ 80 ☐ 160

- **Régime :** ☐ 800 kcal ☐ 1.200 kcal ☐ 1.800 kcal

  suppléments : ☐ non ☐ poisson
  ☐ autre : ____________________

- **Jour de repos digestif :** ☐ riz ☐ pommes de terre/légumes
  ☐ fruits ☐ autre : ____________________

- **Jeûne :**

	miel	☐ non	☐ oui
midi :	jus (¼l)	☐ non	☐ oui
	bouillon (¼l)	☐ non	☐ oui
soir :	bouillon (¼l)	☐ non	☐ oui

  suppléments: ☐ non ☐ Kousmine ☐ Kousmine vegan
  ☐ yoghourt ☐ décoction d'avoine
  ☐ décoction de riz ☐ jus de carotte
  ☐ thé vert ☐ thé noir
  ☐ autre : ____________________

- **Rupture du jeûne**

- **Jour de réalimentation :** ☐ 1 ☐ 2 ☐ 3 ☐ 4

Votre état de santé physique :

très mauvais	0	1	2	3	4	5	6	7	8	9	10	excellent

Votre état de santé émotionnelle :

très mauvais	0	1	2	3	4	5	6	7	8	9	10	excellent

Symptômes depuis l’arrivée à la clinique: ☐ non ☐ oui

☐ maux de tête
☐ trouble du sommeil
☐ haleine chargée
(demandez à l’infirmière)
☐ fatigue
☐ mal de dos
☐ frilosité
☐ vertige
☐ faim
☐ éruption cutanée
☐ ballonnements
☐ douleurs musculaires
☐ brûlures d’estomac
☐ nausée
☐ bouche sèche
☐ crampe au mollet
☐ diarrhée
☐ jambes sans repos
☐ constipation
☐ palpitations
☐ troubles de la vue
☐ envie de manger
☐ autres: ______________________

**Aujourd’hui …**	jamais	de temps en temps	moins de la moitié du temps	plus de la moitié du temps	la plupart du temps	tout le temps
…je me suis senti(e) bien et de bonne humeur	0	1	2	3	4	5
…je me suis senti(e) calme et tranquille	0	1	2	3	4	5
…je me suis senti(e) plein(e) d’énerqie et vigoureux(se)	0	1	2	3	4	5
…je me suis réveillé(e) en me sentant frais(che) et dispos(e)	0	1	2	3	4	5
…ma vie quotidienne a été remplie de choses intéressantes	0	1	2	3	4	5

18. jour : ______________________________

Corps cétoniques (mg/dl): ☐ 0 ☐ 5 ☐ 15 ☐ 40 ☐ 80 ☐ 160

○ **Régime :** ☐ 800 kcal ☐ 1.200 kcal ☐ 1.800 kcal

suppléments : ☐ non ☐ poisson
☐ autre : ______________________________

○ **Jour de repos digestif :** ☐ riz ☐ pommes de terre/légumes
☐ fruits ☐ autre : ____________________

○ **Jeûne :**

	miel	☐ non	☐ oui
midi :	jus (¼l)	☐ non	☐ oui
	bouillon (¼l)	☐ non	☐ oui
soir :	bouillon (¼l)	☐ non	☐ oui

suppléments: ☐ non ☐ Kousmine ☐ Kousmine vegan
☐ yoghourt ☐ décoction d'avoine
☐ décoction de riz ☐ jus de carotte
☐ thé vert ☐ thé noir
☐ autre : ______________________________

○ **Rupture du jeûne**

○ **Jour de réalimentation :** ☐ 1 ☐ 2 ☐ 3 ☐ 4

Votre état de santé physique :

très mauvais	0	1	2	3	4	5	6	7	8	9	10	excellent

Votre état de santé émotionnelle :

très mauvais	0	1	2	3	4	5	6	7	8	9	10	excellent

Symptômes depuis l'arrivée à la clinique: ☐ non ☐ oui

☐ maux de tête
☐ trouble du sommeil
☐ haleine chargée
(demandez à l'infirmière)
☐ fatigue
☐ mal de dos
☐ frilosité
☐ vertige
☐ faim
☐ éruption cutanée
☐ ballonnements
☐ douleurs musculaires
☐ brûlures d'estomac
☐ nausée
☐ bouche sèche
☐ crampe au mollet
☐ diarrhée
☐ jambes sans repos
☐ constipation
☐ palpitations
☐ troubles de la vue
☐ envie de manger
☐ autres: ________________

**Aujourd'hui …**	jamais	de temps en temps	moins de la moitié du temps	plus de la moitié du temps	la plupart du temps	tout le temps
…je me suis senti(e) bien et de bonne humeur	0	1	2	3	4	5
…je me suis senti(e) calme et tranquille	0	1	2	3	4	5
…je me suis senti(e) plein(e) d'énerqie et vigoureux(se)	0	1	2	3	4	5
…je me suis réveillé(e) en me sentant frais(che) et dispos(e)	0	1	2	3	4	5
…ma vie quotidienne a été remplie de choses intéressantes	0	1	2	3	4	5

19. jour : ______________________________

Corps cétoniques (mg/dl): ☐ 0 ☐ 5 ☐ 15 ☐ 40 ☐ 80 ☐ 160

○ **Régime :** ☐ 800 kcal ☐ 1.200 kcal ☐ 1.800 kcal

suppléments : ☐ non ☐ poisson
☐ autre : ______________________

○ **Jour de repos digestif :** ☐ riz ☐ pommes de terre/légumes
☐ fruits ☐ autre : ______________

○ **Jeûne :**

	miel	☐ non	☐ oui
midi :	jus (¼l)	☐ non	☐ oui
	bouillon (¼l)	☐ non	☐ oui
soir :	bouillon (¼l)	☐ non	☐ oui

suppléments: ☐ non ☐ Kousmine ☐ Kousmine vegan
☐ yoghourt ☐ décoction d'avoine
☐ décoction de riz ☐ jus de carotte
☐ thé vert ☐ thé noir
☐ autre : ______________________

○ **Rupture du jeûne**

○ **Jour de réalimentation :** ☐ 1 ☐ 2 ☐ 3 ☐ 4

Votre état de santé physique :

très mauvais	0	1	2	3	4	5	6	7	8	9	10	excellent

Votre état de santé émotionnelle :

très mauvais	0	1	2	3	4	5	6	7	8	9	10	excellent

Symptômes depuis l'arrivée à la clinique: ☐ non ☐ oui

☐ maux de tête
☐ trouble du sommeil
☐ haleine chargée
(demandez à l'infirmière)
☐ fatigue
☐ mal de dos
☐ frilosité
☐ vertige
☐ faim
☐ éruption cutanée
☐ ballonnements
☐ douleurs musculaires
☐ brûlures d'estomac
☐ nausée
☐ bouche sèche
☐ crampe au mollet
☐ diarrhée
☐ jambes sans repos
☐ constipation
☐ palpitations
☐ troubles de la vue
☐ envie de manger
☐ autres: ____________________

**Aujourd'hui …**	jamais	de temps en temps	moins de la moitié du temps	plus de la moitié du temps	la plupart du temps	tout le temps
…je me suis senti(e) bien et de bonne humeur	0	1	2	3	4	5
…je me suis senti(e) calme et tranquille	0	1	2	3	4	5
…je me suis senti(e) plein(e) d'énerqie et vigoureux(se)	0	1	2	3	4	5
…je me suis réveillé(e) en me sentant frais(che) et dispos(e)	0	1	2	3	4	5
…ma vie quotidienne a été remplie de choses intéressantes	0	1	2	3	4	5

20. jour : ____________________

Corps cétoniques (mg/dl): ☐ 0 ☐ 5 ☐ 15 ☐ 40 ☐ 80 ☐ 160

○ **Régime :** ☐ 800 kcal ☐ 1.200 kcal ☐ 1.800 kcal

suppléments : ☐ non ☐ poisson
☐ autre : ____________________

○ **Jour de repos digestif :** ☐ riz ☐ pommes de terre/légumes
☐ fruits ☐ autre : ____________________

○ **Jeûne :**

	miel	☐ non	☐ oui
midi :	jus (¼l)	☐ non	☐ oui
	bouillon (¼l)	☐ non	☐ oui
soir :	bouillon (¼l)	☐ non	☐ oui

suppléments: ☐ non ☐ Kousmine ☐ Kousmine vegan
☐ yoghourt ☐ décoction d'avoine
☐ décoction de riz ☐ jus de carotte
☐ thé vert ☐ thé noir
☐ autre : ____________________

○ **Rupture du jeûne**

○ **Jour de réalimentation :** ☐ 1 ☐ 2 ☐ 3 ☐ 4

Votre état de santé physique :

très mauvais	0	1	2	3	4	5	6	7	8	9	10	excellent

Votre état de santé émotionnelle :

très mauvais	0	1	2	3	4	5	6	7	8	9	10	excellent

Symptômes depuis l'arrivée à la clinique: ☐ non ☐ oui

- ☐ maux de tête
- ☐ trouble du sommeil
- ☐ haleine chargée (demandez à l'infirmière)
- ☐ fatigue
- ☐ mal de dos
- ☐ frilosité
- ☐ vertige
- ☐ faim
- ☐ éruption cutanée
- ☐ ballonnements
- ☐ douleurs musculaires
- ☐ brûlures d'estomac
- ☐ nausée
- ☐ bouche sèche
- ☐ crampe au mollet
- ☐ diarrhée
- ☐ jambes sans repos
- ☐ constipation
- ☐ palpitations
- ☐ troubles de la vue
- ☐ envie de manger
- ☐ autres: ______________________

**Aujourd'hui …**	jamais	de temps en temps	moins de la moitié du temps	plus de la moitié du temps	la plupart du temps	tout le temps
…je me suis senti(e) bien et de bonne humeur	0	1	2	3	4	5
…je me suis senti(e) calme et tranquille	0	1	2	3	4	5
…je me suis senti(e) plein(e) d'énergie et vigoureux(se)	0	1	2	3	4	5
…je me suis réveillé(e) en me sentant frais(che) et dispos(e)	0	1	2	3	4	5
…ma vie quotidienne a été remplie de choses intéressantes	0	1	2	3	4	5

21. jour : ____________________

Corps cétoniques (mg/dl): ☐ 0 ☐ 5 ☐ 15 ☐ 40 ☐ 80 ☐ 160

- **Régime :** ☐ 800 kcal ☐ 1.200 kcal ☐ 1.800 kcal

  suppléments : ☐ non ☐ poisson
  ☐ autre : ____________________

- **Jour de repos digestif :** ☐ riz ☐ pommes de terre/légumes
  ☐ fruits ☐ autre : ____________________

- **Jeûne :**

	miel	☐ non	☐ oui
midi :	jus (¼l)	☐ non	☐ oui
	bouillon (¼l)	☐ non	☐ oui
soir :	bouillon (¼l)	☐ non	☐ oui

  suppléments: ☐ non ☐ Kousmine ☐ Kousmine vegan
  ☐ yoghourt ☐ décoction d’avoine
  ☐ décoction de riz ☐ jus de carotte
  ☐ thé vert ☐ thé noir
  ☐ autre : ____________________

- **Rupture du jeûne**

- **Jour de réalimentation :** ☐ 1 ☐ 2 ☐ 3 ☐ 4

Votre état de santé physique :

très mauvais | 0 | 1 | 2 | 3 | 4 | 5 | 6 | 7 | 8 | 9 | 10 | excellent

Votre état de santé émotionnelle :

très mauvais | 0 | 1 | 2 | 3 | 4 | 5 | 6 | 7 | 8 | 9 | 10 | excellent

Symptômes depuis l'arrivée à la clinique: ☐ non ☐ oui

- ☐ maux de tête
- ☐ trouble du sommeil
- ☐ haleine chargée (demandez à l'infirmière)
- ☐ fatigue
- ☐ mal de dos
- ☐ frilosité
- ☐ vertige
- ☐ faim
- ☐ éruption cutanée
- ☐ ballonnements
- ☐ douleurs musculaires
- ☐ brûlures d'estomac
- ☐ nausée
- ☐ bouche sèche
- ☐ crampe au mollet
- ☐ diarrhée
- ☐ jambes sans repos
- ☐ constipation
- ☐ palpitations
- ☐ troubles de la vue
- ☐ envie de manger
- ☐ autres: ______________________

**Aujourd'hui …**	jamais	de temps en temps	moins de la moitié du temps	plus de la moitié du temps	la plupart du temps	tout le temps
…je me suis senti(e) bien et de bonne humeur	0	1	2	3	4	5
…je me suis senti(e) calme et tranquille	0	1	2	3	4	5
…je me suis senti(e) plein(e) d'énerqie et vigoureux(se)	0	1	2	3	4	5
…je me suis réveillé(e) en me sentant frais(che) et dispos(e)	0	1	2	3	4	5
…ma vie quotidienne a été remplie de choses intéressantes	0	1	2	3	4	5

22. jour : ______________________

Corps cétoniques (mg/dl): ☐ 0 ☐ 5 ☐ 15 ☐ 40 ☐ 80 ☐ 160

○ **Régime :** ☐ 800 kcal ☐ 1.200 kcal ☐ 1.800 kcal

suppléments : ☐ non ☐ poisson
☐ autre : ______________________

○ **Jour de repos digestif :** ☐ riz ☐ pommes de terre/légumes
☐ fruits ☐ autre : ______________

○ **Jeûne :**

	miel	☐ non	☐ oui
midi :	jus (¼l)	☐ non	☐ oui
	bouillon (¼l)	☐ non	☐ oui
soir :	bouillon (¼l)	☐ non	☐ oui

suppléments: ☐ non ☐ Kousmine ☐ Kousmine vegan
☐ yoghourt ☐ décoction d'avoine
☐ décoction de riz ☐ jus de carotte
☐ thé vert ☐ thé noir
☐ autre : ______________________

○ **Rupture du jeûne**

○ **Jour de réalimentation :** ☐ 1 ☐ 2 ☐ 3 ☐ 4

Votre état de santé physique :

très mauvais	0	1	2	3	4	5	6	7	8	9	10	excellent

Votre état de santé émotionnelle :

très mauvais	0	1	2	3	4	5	6	7	8	9	10	excellent

Symptômes depuis l’arrivée à la clinique: ☐ non ☐ oui

☐ maux de tête
☐ trouble du sommeil
☐ haleine chargée
(demandez à l’infirmière)
☐ fatigue
☐ mal de dos
☐ frilosité
☐ vertige
☐ faim
☐ éruption cutanée
☐ ballonnements
☐ douleurs musculaires
☐ brûlures d’estomac
☐ nausée
☐ bouche sèche
☐ crampe au mollet
☐ diarrhée
☐ jambes sans repos
☐ constipation
☐ palpitations
☐ troubles de la vue
☐ envie de manger
☐ autres: ____________________

**Aujourd’hui …**	jamais	de temps en temps	moins de la moitié du temps	plus de la moitié du temps	la plupart du temps	tout le temps
…je me suis senti(e) bien et de bonne humeur	0	1	2	3	4	5
…je me suis senti(e) calme et tranquille	0	1	2	3	4	5
…je me suis senti(e) plein(e) d’énerqie et vigoureux(se)	0	1	2	3	4	5
…je me suis réveillé(e) en me sentant frais(che) et dispos(e)	0	1	2	3	4	5
…ma vie quotidienne a été remplie de choses intéressantes	0	1	2	3	4	5

Questions à la fin du séjour

1) Présentiez-vous un problème de santé majeur au début de votre séjour ?
   ☐ non
   ☐ oui, lequel ? ____

   ____

   Comment ce problème de santé majeur a-t'il évolué au cours du séjour ?

grave péjoration	1	2	3	4	5	6	7	très grande amélioration

2) Comment avez-vous ressenti votre jeûne ou votre régime basse calorie pendant votre séjour:

un calvaire	1	2	3	4	5	6	7	une expérience magnifique

3) Comparez votre séjour ici à un autre type de vacances. Buchinger Wilhelmi est:

   ☐ moins relaxant ☐ également relaxant ☐ plus relaxant

4) La durée de votre séjour fut:

   ☐ trop courte ☐ adéquate ☐ trop longue

5) Au moment de l'interruption du jeûne, auriez-vous eu envie de continuer à jeûner ?

   ☐ oui ☐ non

Style de vie – pendant la durée de votre séjour

Niveau de stress/relaxation :

état de tension extrême	0	1	2	3	4	5	6	7	8	9	10	totalement relaxé

**Nicotine :**	☐ non	☐ cigarettes/jour:	
	☐ non	☐ cigares/jour:	
**Alcool :**	☐ non	☐ bière : verre (0,5 l)/semaine:	
	☐ non	☐ vin : verre (0,25 l)/semaine:	
	☐ non	☐ alcool fort : verre (2 cl)/semaine:	
**Café :**	☐ non	☐ tasse/jour:	
**Médias :**	☐ non	☐ smart-phone (h/semaine):	
	☐ non	☐ télévision (h/semaine):	
	☐ non	☐ ordinateur (h/semaine):	
**Exercice physique :**	☐ non	☐ oui : h/semaine:	,

**Au cours des dernières semaines …**	jamais	de temps en temps	moins de la moitié du temps	plus de la moitié du temps	la plupart du temps	tout le temps
…je me suis senti(e) bien et de bonne humeur	0	1	2	3	4	5
…je me suis senti(e) calme et tranquille	0	1	2	3	4	5
…je me suis senti(e) plein(e) d'énergie et vigoureux(se)	0	1	2	3	4	5
…je me suis réveillé(e) en me sentant frais(che) et dispos(e)	0	1	2	3	4	5
…ma vie quotidienne a été remplie de choses intéressantes	0	1	2	3	4	5

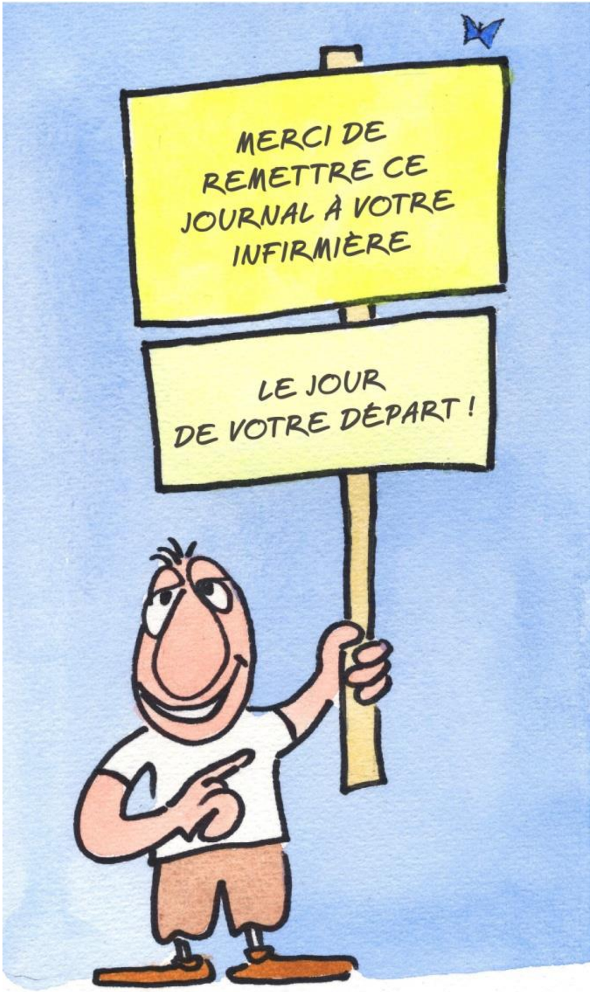
MERCI DE
REMETTRE CE
JOURNAL À VOTRE
INFIRMIÈRE
LE JOUR
DE VOTRE DÉPART !

Les réponses à ces questions ne sont pas indispensables pour notre documentation scientifique mais aident Buchinger Wilhelmi à améliorer ses prestations :

Comment avez-vous trouvé le pensum quotidien de remplir ce « journal d'observation personnelle » ?

Auriez-vous des propositions d'amélioration ?

Est-ce que le séjour à répondu à votre attente ?

Pensez-vous que votre séjour va influencer votre vie et dans quel(s) domaine(s) ?

Notre service « Feel well after Buchinger Wilhelmi » offre par Skype la possibilité de parler avec une nutritionniste si vous le désirez.
E-mail : nutrition@buchinger-wilhelmi.com

Merci beaucoup et à bientôt !

Nous vous écrirons par email dans deux semaines, trois et onze moins pour prendre de vos nouvelles.

Bon retour chez vous !
Follow up Buchinger Wilhelmi
